# Novel Non-Congeneric Derivatives of the Choline Kinase Alpha Inhibitor ICL-CCIC-0019

**DOI:** 10.3390/pharmaceutics13071078

**Published:** 2021-07-14

**Authors:** Ning Wang, Diana Brickute, Marta Braga, Chris Barnes, Haonan Lu, Louis Allott, Eric O. Aboagye

**Affiliations:** 1 Comprehensive Cancer Imaging Centre, Department of Surgery and Cancer, Faculty of Medicine, Imperial College London, Hammersmith Hospital, London W12 0NN, UK; n.wang16@imperial.ac.uk (N.W.); dianabrickute@yahoo.co.uk (D.B.); marta.costabraga@icr.ac.uk (M.B.); chris.barnes@imperial.ac.uk (C.B.); haonan.lu12@imperial.ac.uk (H.L.); 2 Positron Emission Tomography Research Centre, Faculty of Health Sciences, University of Hull, Kingston upon Hull HU6 7RX, UK

**Keywords:** choline kinase alpha (CHKA) inhibitor, ICL-CCIC-0019, prodrug, PSMA, targeted drug delivery, PIK4CB

## Abstract

Choline kinase alpha (CHKA) is a promising target for the development of cancer therapeutics. We have previously reported ICL-CCIC-0019, a potent CHKA inhibitor with high cellular activity but with some unfavorable pharmacological properties. In this work, we present an active analogue of ICL-CCIC-0019 bearing a piperazine handle (CK146) to facilitate further structural elaboration of the pharmacophore and thus improve the biological profile. Two different strategies were evaluated in this study: (1) a prodrug approach whereby selective CHKA inhibition could be achieved through modulating the activity of CK146, via the incorporation of an ε-(Ac) Lys motif, cleavable by elevated levels of histone deacetylase (HDAC) and cathepsin L (CTSL) in tumour cells; (2) a prostate-specific membrane antigen (PSMA) receptor targeted delivery strategy. Prodrug (CK145) and PSMA-targeted (CK147) derivatives were successfully synthesized and evaluated in vitro. While the exploitation of CK146 in those two strategies did not deliver the expected results, important and informative structure-activity relationships were observed and have been reported.

## 1. Introduction

Abnormal lipid metabolism is a common feature observed in many cancers [1]. Enhanced de novo synthesis of lipids in tumorigenesis supports the rapid proliferation of cancer cells, cell signaling and tumor survival [2]. The cholinic phenotype, characterized by the overexpression of choline kinase alpha isoform (CHKA) and increased phosphocholine (PCho) levels, is one of the aberrant lipid metabolism pathways revealed in cancer [3]. Choline kinase is a class of enzymes responsible for generating PCho which is involved in the biosynthesis of phosphatidylcholine, a major lipid component of cell membranes [4]. Choline kinase exists in at least three isoforms, CHKA1, CHKA2 and CHKB [4], of which the A, but not the B isoform, has been implicated in cancer development [3,5]. Overexpression of CHKA has been reported in a wide range of solid tumors [3,5,6,7,8,9,10,11,12], and correlates with advanced histological tumor grade, poor prognosis and reduced survival rates in breast and non-small-cell lung cancers [9,13]. In prostate cancer (PCa), CHKA is a co-chaperone for the androgen receptor (AR), thus maintains AR signaling [14]. Taken together, CHKA is a prognostic marker and a potential therapeutic target for cancer treatment.

**Figure 1:**
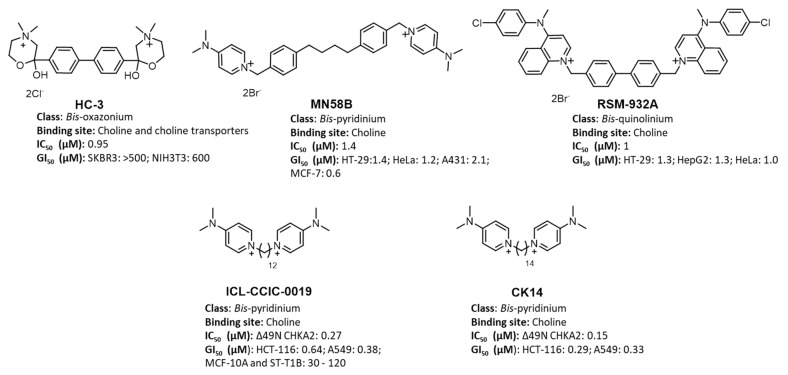
Chemical structures and pharmacological data of corresponding CHKA inhibitors: HC-3 [18,25], MN58B [20,21], RSM-932A [23], ICL-CCIC-0019 [26,27] and CK14 [26].

A number of small-molecule CHKA inhibitors have been developed as novel anti-cancer strategies for CHKA inhibition [15]. Hemicholinium-3 (HC-3) was the first CHKA inhibitor built around a bis-oxazonium pharmacophore (Figure 1) [16,17,18], but exhibited off-target effects on choline transporters, acetyltransferase and acetylcholinesterase [19]. MN58B was subsequently designed by structural modifications of HC-3 and demonstrated good antiproliferative potencies in vitro and in vivo (Figure 1) [20,21,22]. The replacement of pyridiniums in MN58B by quinoliniums as cationic head groups elicited the best results in vitro and in vivo, and gave rise to RSM-932A (Figure 1), the first CHKA inhibitor to enter Phase I clinical trials [23,24].

In recent years, the x-ray crystal structure of CHKA, together with computational in silico techniques have boosted the development of CHKA inhibitors (the crystal structure of human CHKA, pdb code: 2CKO) [18,20,28,29,30,31,32,33,34,35,36]. A series of symmetrical *N*,*N*-dimethylaminopyridine (DMAP) derivatives bearing alkyl linkers of varying lengths were designed by Trousil et al. as CHKA inhibitors (Figure 1), where DMAP mimicked cationic choline moieties [26]; from this study, ICL-CCIC-0019 was selected for further biological evaluations and displayed great potential in preclinical studies [27]. ICL-CCIC-0019 selectively inhibited CHKA activity with potent antiproliferative activities against a broad spectrum of human cancer cell lines (IC_50_: 0.27 ± 0.06 μM; mean GI_50_ of 8 cancer cell lines: 1.09 μM, range: 0.38–2.70 μM; GI_50_ range of 2 normal cell lines: 30–120 μM); ICL-CCIC-0019 caused rapid and sustained lipid synthesis inhibition by acting on CDP-choline pathway and also showed potent in vivo anti-tumor activity. The compound, however, induced mitochondrial changes and slight reduction in body weight (not below the standard 15–20%) observed in the rodent model bearing HCT116 xenografts; implying adverse pharmacological effects which precluded clinical translation [25]. Modulating the unfavorable properties of ICL-CCIC-0019 was likely to be challenging for two reasons: firstly, the intrinsic toxicity of quaternary ammonium CHKA inhibitors is difficult to overcome since their structures mimic choline and the inhibition of choline transporters was observed for these molecules [26]. Secondly, the limited structural diversity of ICL-CCIC-0019 makes direct modification of the molecule less feasible. While the quaternary ammonium moieties are essential to the activity and could not be modified, we hypothesized that controlling the release or delivery of potent CHKA inhibitors in target tissue may reduce off-target accumulation and toxicity. These strategies have been exemplified for a range of compounds and biological targets, using either a prodrug strategy, whereby compound potency is masked in off-target tissue and selectively released in target tissue, or a receptor-targeted delivery strategy where the drug concentration in target tissue is controlled based on receptor expression [37,38].

This study evaluated two strategies:

(1) Enzymatic prodrug strategy whereby an ε-(Ac)Lys peptide motif is cleaved stepwise by upregulated histone deacetylase (HDAC) and endogenous protease cathepsin L (CTSL) in malignant cells [39,40,41,42];
(2) Peptide-receptor targeted drug delivery strategy targeting prostate specific membrane antigen (PSMA), a transmembrane receptor overexpressed (100–1000 fold higher) in PCa [43,44].

To exploit these non-congeneric strategies for our purposes, a scaffold based around ICL-CCIC-0019 with comparable CHKA activity would be required to bear a reactive handle for further structural elaboration. We report the synthesis of a novel potent asymmetrical CHKA active analogue of ICL-CCIC-0019 for evaluation in the aforementioned strategies, and two novel drug conjugates evaluated in vitro.

## 2. Materials and Methods

### 2.1. Chemical Synthesis

The chemical synthesis procedures of ICL-CCIC-0019, CK14, CK145 (5), CK146 (4), CK147 (8) and CK148 (7) are described in the Supplementary Information. The radiosynthesis of [^18^F]D4-FCH has been reported elsewhere [45].

### 2.2. Cell Culture

HCT-116, A549, 22Rv1, C4-2B, LNCap, R1-AD1, R1-D567, PC3 and PNT1A were cultured with RPMI 1640 media (Sigma Life Science, Gillingham, UK). HepG2 and Caco-2 were cultured with DMEM media (BioWhittaker^®^, Lonza, Basel, Switzerland). All the media were supplemented with 10% FCS (Fetal Calf Serum, First Link UK Ltd., Wolverhampton, UK) and 10% L-glutamine (gibco^®^, Life Technologies, Paisley, UK; 200 mmol, 100 mL). Caco-2 cell line was maintained in DMEM media (BioWhittaker^®^, Lonza, Basel, Switzerland) containing 20% FCS (Fetal Calf Serum, First Link UK Ltd., Wolverhampton, UK) and 10% L-glutamine (gibco^®^, Life Technologies, Paisley, UK; 200 mmol, 100 mL). Cells were cultured at 37 °C in a humidified atmosphere containing 5% CO_2_. Cell lines were authenticated by provider (Human colorectal adenocarcinoma cell line, ATCC). No additional authentication of cells was performed.

### 2.3. Anti-Proliferative Assay (Sulforhodamine B Assay)

Half-maximal growth inhibitory concentrations (GI_50_) were determined using sulforhodamine B (SRB) assay as described elsewhere [46]. In brief, cells were seeded in 96-wells at respective cell densities determined by their cell growth curves. After 24 h, the cells were treated in six replicates with medium or different concentrations of CHKA inhibitors in medium and incubated for another 72 h. On completion of incubation, the cells were fixed with 10% trichloroacetic acid (TCA) and stained with 0.4% SRB in 1% acetic acid. The stained protein was solubilized with 10 mM Tris base and optical density values were measured at 540 nm using a Multiskan^®^ EX Micro plate photometer (Thermo Scientific, Loughborough, UK). Growth inhibition curves were plotted as percentage of the control groups and GI_50_ data were determined by least squares fitting method using GraphPad Prism 7.0. All the chemicals used in this experiment were obtained from Sigma-Aldrich (Gillingham, UK).

### 2.4. Lipid Kinase Screening

The lipid kinase screening for ICL-CCIC-0019, CK14, CK145 (5), CK146 (4) and CK147 (8) (testing concentration for all compounds: 10 μM) was performed by MRC PPU International Centre for Kinase Profiling (Dundee, UK). The lipid kinase screening panel includes 15 human recombinant lipid kinases and the tests used Promega ADP Glo^TM^ High Throughput Assay kit (Promega, Southampton, UK). For each assay, reference compounds were used as quality control. Finally, mean percentage activity inhibited by compounds and standard deviation for all the repeats were calculated.

### 2.5. In Vitro ‘Absorption, Distribution, Metabolism and Excretion’ (ADME) Study

The ADME study for ICL-CCIC-0019, CK14, CK145 (5), CK146 (4) and CK147 (8) was performed by Charles River Drug Discovery Services (Essex, UK).

### 2.6. Immunoblotting Analysis (General)

Cell pellets were prepared and lysed with radioimmunoprecipitation assay (RIPA) buffer (R0278, Sigma-Aldrich, Gillingham, UK) supplemented with protease and phosphatase inhibitors (Thermo Fisher, Loughborough, UK). Equal amounts of protein (10 μg) were resolved on 4–15% mini-protein TGX gels (456-1086 Bio-Rad) and transferred to PVDF membranes (Trans-Blot Turbo Transfer Packs, Bio-Rad, Watford, UK). Membranes were blocked with 5% milk in phosphate buffered saline (PBS) containing 0.1% *v*/*v* tween-20 and subsequently incubated with the following primary antibodies at 4 °C overnight: HDAC1 (10E2), 1:8000, #5356s, Cell Signaling Technology, London, UK; Calnexin, 1:5000, #ADI-SPA-860-D, ENZO life sciences, Exeter, UK; CHKA, 1:1000, #HPA024153, Sigma-Aldrich, Gillingham, UK; PSMA (D78E), 1:1000, #12815, Cell Signaling Technology, London, UK; GAPDH (D16H11), 1:10,000, #5174, Cell Signaling Technology, London, UK. Secondary HRP-conjugated mouse and rabbit antibodies (1:8000 and 1:5000; #7076P2 and #sc-2005; Cell Signaling Technology, London, UK and Santa Cruz Biotechnology, California, USA, respectively) were used for 1 h at room temperature. Signals were visualized by chemiluminescence detection using Amersham ECL Western Blotting Detection Reagent (GE Healthcare, Amersham, UK) and Amersham Hyperfilm (GE Healthcare, Amersham, UK).

### 2.7. Conjugate Stability Test of CK147 (8)

To assess stability of CK147 in medium, 5 mL of medium containing 10% FCS and 1% L-glutamine was added sterilely to a six-well plate. Next, 1.7 μL of 300 mM CK147 in DMSO or 1.7 μL DMSO was added into wells and the plate was gently shaken for 5 s before being incubated at 37 °C in a humidified atmosphere containing 5% CO_2_. After 0, 2, 4, 6, 24, 48 and 72 h, 200 μL aliquots of medium from each well were transferred into a 1.5-mL tube containing 198.4 μL acetonitrile and 1.6 μL of 2.5 mg/mL ICL-CCIC-0019 as an internal standard (IS) (20 μg/mL). After a brief vortex, the Eppendorf tube was placed at 4 °C for 30 min for protein precipitation. Fifty microliters of the resulting supernatant from centrifuge (4 °C, 15,000× *g*) was taken to supplement with 70 μL of water:acetonitrile:formic acid (95:5:0.2, *v*/*v*/*v*) and sonicated for 1 min. One hundred microliters of the solution were injected into the HPLC system described in Supplementary HPLC method D. Three repeats were performed for each sample.

### 2.8. Cellular Uptake and Metabolism Study of CK145 (5)

HCT-116 colorectal cancer cells were seeded into cell culture dishes (150 × 25 mm) at a density of 7 × 10^6^ per dish. The cells were allowed to settle and grow for another 48 h before being treated with or without ICL-CCIC-0019 or CK145 (50 μM of 5 mL RPMI medium with 10% FCS and 1% L-glutamine) for 1 h and 2 h in the incubator. Cells were scraped, washed with ice-cold PBS three times and collected by centrifugation (4 °C, 4000× *g*). The cell pellets were resuspended in 1 mL water: acetonitrile: formic acid (95:5:0.2, *v*/*v*/*v*) and disrupted by an ultrasonic cell disruptor (Soniprep 150, MSE Ltd., London, UK) for 1 min on ice. One hundred microliters of the filtered supernatant from centrifuge (4 °C, 15,000× *g*) were analyzed by HPLC. Three injections were performed for each sample. Intracellular drug was normalized to cellular protein of each plate using bicinchoninic acid (BCA) assay. The HPLC method used in this study is described in Supplementary HPLC method C.

### 2.9. Cellular Uptake and Metabolism Study of ICL-CCIC-0019, CK146 (4), CK147 (8) and CK148 (7) in Presence and Absence of Serum

Cell lysate sample preparation: C4-2B prostate cancer cells were seeded into cell culture dishes (150 × 25 mm) at a density of 7 × 10^6^ per dish. The cells were allowed to settle and grow for another 48 h. The cells were treated with ICL-CCIC-0019, CK146, CK147 and CK148 (50 μM of 7 mL RPMI medium with or without 10% FCS) for 4 h in the incubator. After 4-h incubation, the aliquots of medium were collected (see the medium sample preparation section). Cells were scraped, washed with ice-cold PBS three times and collected by centrifugation (4 °C, 4000× *g*). The cell pellets were resuspended in 500 μL of chilled acetonitrile:PBS (50:50, *v*/*v*) for cell disruption and homogenization. The cell disruption was performed by a Precellys^®^ 24 homogenizer (Bertin Technologies, Montigny-le-Bretonneux, France) at 4 °C. After acetonitrile evaporation at room temperature, 50 μL of cell lysis was taken for protein analysis. Another 100 μL of the supernatant obtained after centrifuge (4 °C, 15,000× *g*) was resuspended in 100 μL formic acid:water (0.2%, *v*/*v*). Next, 100 μL of the filtered solution was analyzed by HPLC system described in Supplementary HPLC method D. Three injections were performed for each sample. Intracellular drug was normalized to cellular protein of each plate using BCA assay. Medium sample preparation for HPLC analysis: 250-μL aliquots of medium (with or without 10% FCS) were transferred into 1.5-mL tube containing 250 μL acetonitrile. After a brief vortex, the tube was placed at 4 °C for 30 min for protein precipitation. One hundred microliters of the resulting supernatant from centrifuge (4 °C, 15,000× *g*) were resuspended in 100 μL formic acid: water (0.2%, *v*/*v*). One hundred microliters of the filtered sample were analyzed by HPLC system described in Supplementary HPLC method D. Three injections were performed for each sample.

### 2.10. [^18^F]D4-FCH Uptake In Vitro Study

HCT-116 and C4-2B cells were seeded at a density of 5 × 10^5^ cells per well into 6-well plates and incubated overnight. Cells were treated with 1 or 10 µM CHKA inhibitors (CK14, CK145, CK146 and CK147) for 1 h and pulsed with [^18^F]D4-FCH (740 kBq) in presence of inhibitors in 1 mL for an additional hour at 37 °C. Cells were washed with ice-cold PBS three times and lysed in 1 mL RIPA buffer. The radioactivity of 800 µL lysate from each well was counted on a gamma counter (Perkin Elmer, Beaconsfield, UK) and normalized to protein content as determined by BCA assay.

### 2.11. Statistical Analysis

Data were represented as mean values ± standard deviation (SD). Unpaired two-tailed *t*-tests from GraphPad Prism 7.0 were used to determine the significance in the experiments. Statistic differences were significant at 0.01 < *p* < 0.05, very significant at 0.001 < *p* < 0.01 and extremely significant at 0.0001 ≤ *p* < 0.001 and *p* < 0.0001.

## 3. Results

### 3.1. Synthesis of Novel CHKA Inhibitors

**Scheme 1:**
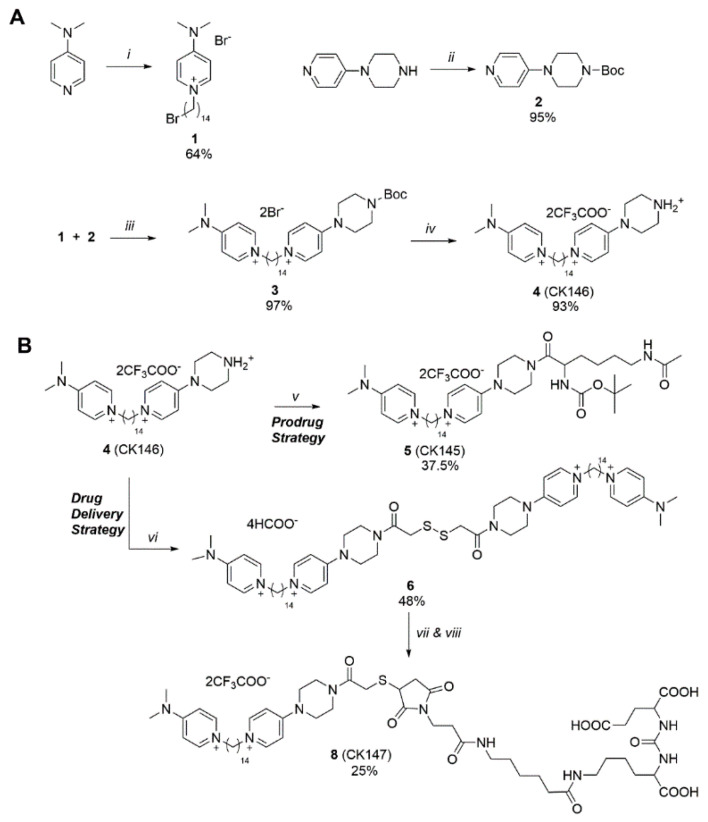
The modifications of CK146 via the prodrug strategy and drug delivery strategy. (**A**) Synthesis of CK146 (4); reaction conditions: (i) 1,14-bromotetradecane, 2-butanone, 110 °C, 3 h; (ii) Di-tert-butyl decarbonate, DMAP, DCM, RT, 16 h; (iii) MeCN, 110 °C, 72 h; (iv) TFA, MeCN, RT, 16 h; (**B**) Synthesis of CK145 (5) and CK147 (8); reaction conditions: (v) α-Boc-Lys (ε-Ac)-OH, EDC HCl, HOBt, DIEA, DMF, RT, 24 h; (vi) Dithiodiglycolic acid, EDC HCl, HOBt, DIPEA, DMF, RT, 72 h; (vii) TCEP, Tris-HCl, NaOH, PSMA-Maleimide (9), DMF, N_2_, RT, 24 h; (viii) TFA, DCM, RT, 16 h.

The synthesis of CK146 from building blocks 1 and 2 is described in Scheme 1A. The nonsymmetric tetradecyl linker (1) was accessed by reacting 1,14-bromotetradecane with DMAP to afford the product as a white precipitate [26]. Compound 2 was synthesized from 1-(pyridin-4-yl) piperazine by boc-protection of the secondary amine in an excellent yield (94%). Quaternization of the pyridinyl ring of 2 by coupling with 1 produced compound 3 in a near quantitative yield (97%) which was subsequently deprotected under acidic conditions to give CK146 (4) in a yield of 93%.

The synthesis of CK145 (5) was performed by contract synthesis (Creative Chemistry Ltd., Hampton, UK) following a literature procedure [47]. In brief, compound 4 was coupled with commercially available α-Boc-Lys (ε-Ac)-OH in the presence of EDC HCl, HOBt and DIEA in DMF at room temperature (Scheme 1B, prodrug strategy). CK145 was purified by semi-prep HPLC which produced pure product (>98%) in a combined yield of 37.5% ready for biological evaluation (see Supplementary, HPLC method B). The synthesis of CK148 (7) is shown in Scheme 1B (drug delivery strategy). Dimer 6 was synthesized by peptide coupling with dithiodiglycolic acid which was reduced to a monomer in the presence of TCEP hydrochloride in degassed Tris-HCl buffer (pH 7) followed by the dropwise addition of 9 ((OtBu)_3_ PSMA-maleimide, Creative Chemistry Ltd., Hampton, UK). This produced 7 in a moderate yield of 56% which was deprotected to produce the final compound CK147 (8) ready for biological evaluation without further purification (purity: 95% determined by HPLC; Figure S31). All compounds were characterized by NMR and Mass Spectrometry (spectra shown in the Supplementary, Figures S7–S30).

### 3.2. Conjugate Stability Test of CK147 (8)

**Figure 2:**
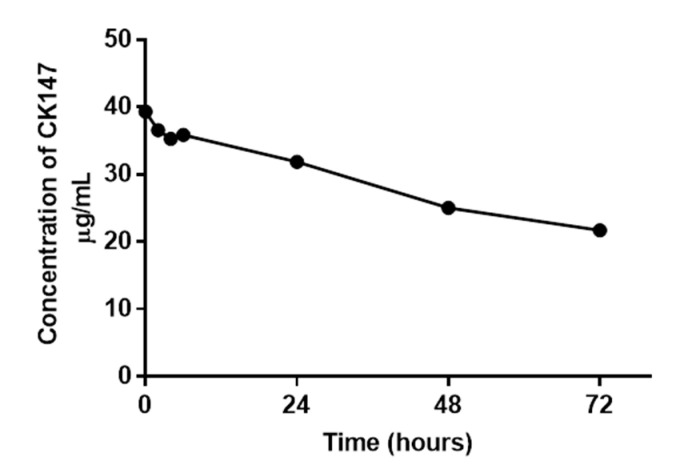
PSMA-targeted CHKA inhibitor (CK147) concentration trend (72 h) in medium supplemented with 10% FCS (60 ± 0.3% intact parent at 72 h); *n* = 3, mean ± SD.

The conjugate stability of CK147 was assessed by HPLC, where ICL-CCIC-0019 served as an internal standard. At the early time points, CK147 remained stable in medium at 37 °C (93.0 ± 0.8% intact parent at 2 h and 89.7 ± 0.3% intact parent at 4 h). After 72 h, the concentration of CK147 in medium reduced to 60.0 ± 0.3% (21.7 ± 0.2 μg/mL) (Figure 2). Chemical decomposition was not detected by HPLC/MS analysis. A hypothesis for this phenomenon is further elaborated in the Discussion.

### 3.3. Antiproliferative Activity Assays

**Figure 3:**
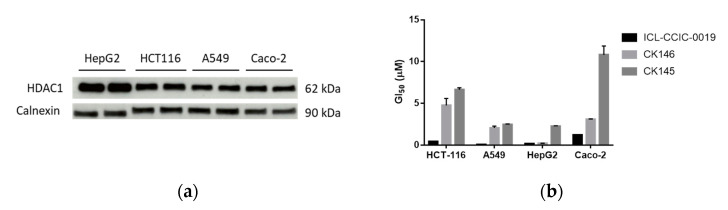
Antiproliferative activity characterization of ICL-CCIC-0019, CK146 and CK145. (**a**) Immunoblotting analysis of HDAC1 in HepG2, HCT-116, A549 and Caco-2 cell lines (*n* = 2); (**b**) Comparison of growth inhibitory activity among ICL-CCIC-0019, CK146 and CK145 in HCT-116, A549, HepG2 and Caco-2 cells (*n* = 6, mean ± SD).

Appropriate cell lines were selected on the basis of mRNA levels of HDAC I class and CTSL using Cancer Cell Line Encyclopedia RNA sequencing database (Table S1). The HDAC 1 subtype was found highly expressed in all four selected cell lines as determined by immunoblotting (Figure 3a). The antiproliferative activities (GI_50_) of ICL-CCIC-0019, CK146 and CK145 were evaluated in these four cell lines by SRB assay. It was shown that all the three compounds potently inhibited the proliferation of cancer cells at low sub-micromolar concentrations (Figure 3b, Table S2). ICL-CCIC-0019 exhibited the best antiproliferative potency (mean GI_50_ in four cancer cell lines: 0.5 ± 0.02 μM); CK146 still displayed potent antiproliferative activity (mean GI_50_ against four cancer cell lines: 2.5 ± 0.3 μM) with HepG2 being most sensitive to the treatment (0.21 ± 0.01 μM). The overall antiproliferative activity of CK145 was similar to CK146 (mean GI_50_ against four cancer cell lines: 5.0 ± 0.3 μM), but it resulted in less growth inhibition in HepG2 and Caco-2 cells. All three compounds displayed weaker antiproliferation in Caco-2 cells—a less tumorigenic colon cancer cell line—compared to HCT-116 cells.

**Figure 4:**
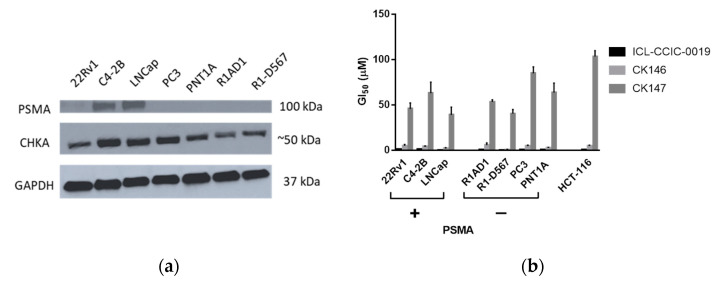
Antiproliferative activity characterization of ICL-CCIC-0019, CK146 and CK147. (**a**) Immunoblotting analysis of PSMA and CHKA in 22Rv1, C4-2B, LNCap, PC3, PNT1A, R1-AD1 and R1-D567 cell lines (*n* = 2); (**b**) Growth inhibitory activity comparison among ICL-CCIC-0019, CK146 and CK147 in 22Rv1, C4-2B, LNCap, PC3, PNT1A, R1-AD1, R1-D567 and HCT-116 cell lines (*n* = 6, mean ± SD).

In addition, the antiproliferative activity of CK147 was assessed in PSMA-positive (22Rv1, C4-2B, LNCap) and PSMA-negative cell lines (PC3, PNT1A, R1-AD1, R1-D567 and HCT-116). The expression of CHKA and PSMA in prostate cell lines was determined by immunoblotting (Figure 4a). ICL-CCIC-0019 and CK146 showed potent antiproliferative activities, with mean GI_50_ across eight cell lines of 0.6 ± 0.1 μM and 3.8 ± 0.9 μM respectively (Figure 4b). However, all the tested cell lines were not sensitive towards CK147 (mean GI_50_: 66.7 ± 7.2 μM) and the correlation of GI_50_ with PSMA expression was not observed.

### 3.4. Cellular Uptake and In Vitro Metabolism

**Figure 5:**
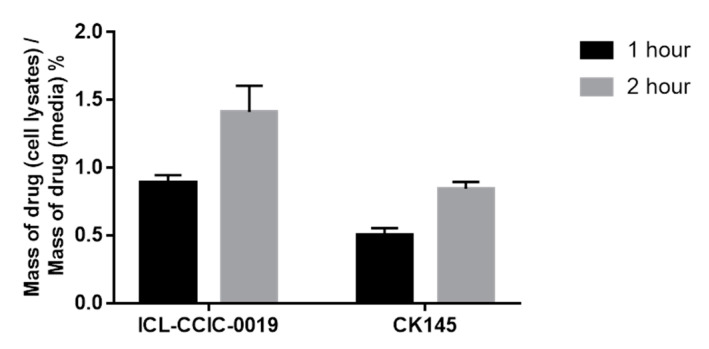
Percentage drug uptake in HCT-116 cell line determined by HPLC at selected time points (1-h and 2-h) (*n* = 3, mean ± SD). The data were calculated by the drug amount in cells compared to the initial drug amount in media %; the values represent the percentage of drugs taken into cells.

Uptake of ICL-CCIC-0019 and CK145 in HCT-116 cells increased with incubation time (Figure 5). The cellular uptake of ICL-CCIC-0019 was approximately two-fold higher than CK145 at 1 h and 2 h post-incubation. However, in vitro conversion of CK145 was not observed (Figures S32 and S33); CK146 or intermediate species were not detected in the media, which could arise from cellular active efflux (Figure S34). The cellular concentration of converted CK145 products was found to be lower than the limit of detection (LOD) of the HPLC method used (1.382 μg/L).

**Figure 6:**
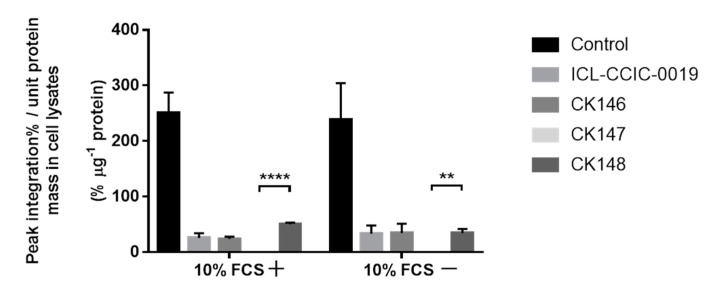
Drug uptake percentage following 4-h incubation with ICL-CCIC-0019, CK146, CK147 and CK148 in medium with or without FCS (*n* = 3, mean ± SD). The data were calculated from chromatographic peak integration normalized to protein amount in cell lysates. *p* Values via unpaired two-tailed *t*-test: **** *p* < 0.0001, ** *p* < 0.01.

An in vitro uptake study using C4-2B cell line was conducted to investigate the cellular uptake and metabolism of CK147. The study comprised two experimental conditions to evaluate the effects of serum on drug uptake: one group included medium supplemented with 10% FCS (FCS positive group) and the other was FCS-free medium (FCS negative group). ICL-CCIC-0019, CK146 and CK148 ((OtBu)_3_-protected CK147) were included in the experiment for comparison. In both experiment groups, ICL-CCIC-0019 (t_R_: 7.36 min: sec), CK146 (t_R_: 6.30 min: sec) and CK148 (t_R_: 11.30 min: sec) were detected in the cell lysates by HPLC following 4 h incubation, while uptake of CK147 was not seen (Figure S35). ICL-CCIC-0019 and CK146 had similar mean uptake of 24.9 ± 1.4% μg^−1^ protein and 34.1 ± 0.6% μg^−1^ protein for FCS positive and negative groups respectively (Figure 6). Significant increase in the cellular uptake in both experiment groups was seen in CK148 (mean ± SD of two experiment groups: 42.5 ± 11.5% μg^−1^ protein) compared to CK147 (Figure 6). Drug uptake was independent to the presence of FCS.

HPLC/MS analysis of cell lysates and the medium after 4 h incubation with CHKA inhibitors confirmed the stability of all the compounds in cells and medium. Decomposition or interconversion was not observed from the HPLC/MS (Figures S36–S45).

### 3.5. ADME Study

The predicted in vitro pharmacokinetic characteristics of compounds were evaluated by four ADME assays, namely kinetic solubility assay, metabolic stability assay using human liver microsome S9, permeability and efflux study using the Caco-2 cell line, and matrix stability assay using fasted state simulated gastric fluid (FaSSGF). All tested CHKA inhibitors were highly soluble (>150 μM) in 0.1 M PBS (2% DMSO) (Table 1). All CHKA inhibitors except for CK145 were stable in the metabolic study; ICL-CCIC-0019, CK14, CK145 and CK146 had long biological half-lives (>100 min) and therefore lower clearance values (<14 µL/min/mg) (Table 1). The in vivo clearance was calculated to predict the actual clearance in humans by scaling the clearance values, which takes into account liver weight, microsomes per liver and liver blood flow. By contrast, CK145 had a short half-life value (7.4 min) and a predicted clearance of 19 mL/min/kg, giving a value for the percentage of compound removed compared to liver blood flow (PCT_LBF%) up to 92%. In Caco-2 cell permeability assay, drug permeability was investigated and presented as apparent permeability coefficients (Papp) in A to B (A-B, apical to basolateral direction) and B to A (B-A, basolateral to apical direction). All the tested compounds exhibited low intestinal permeability classification (Papp A-B < 5 × 10^−6^ cm/s). Notably, CK14 was the most subject to efflux with an efflux ratio of 87; the permeability of CK147 was too poor to permit calculation of the efflux ratio.

### 3.6. Lipid Kinase Screening

**Figure 7:**
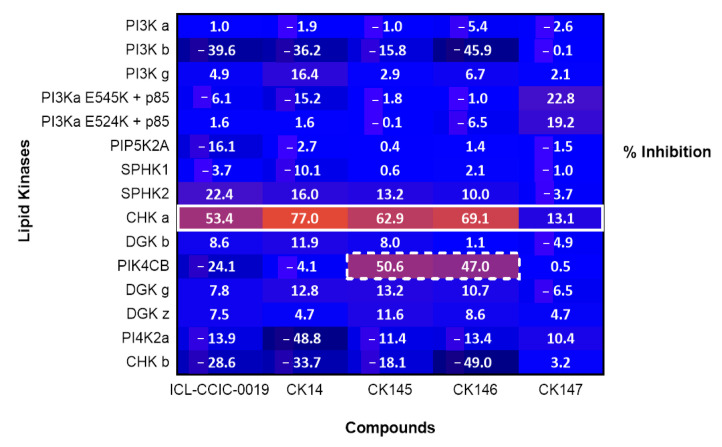
The heatmap of lipid kinase selectivity screening against 15 lipid kinases using ICL-CCIC-0019, CK14, CK145, CK146 and CK147. The inhibition % of CHKA and PIK4CB are boxed in white and dash-line rectangles respectively. The data in percentage represent the mean kinase activity inhibited by drug molecules. Tested concentration of the CHKA inhibitors in the assay: 10 μM.

The selectivity of ICL-CCIC-0019, CK14, CK145, CK146 and CK147 against 15 human lipid kinases was assessed using an ADP-Glo^TM^ Kinase Assay. This assay measured generated ADP as a function of the kinase activity via a luciferase reaction (Figure 7). Four of the CHKA inhibitors displayed good inhibitory activities against CHKA (53–77%) while not affecting CHKB at the tested concentration (10 μM), except for CK147. The tetradecyl linker exhibited superior CHKA affinity compared to the dodecyl linker, which supports our previous structure-activity relationship (SAR) data [26]. CK14 was the most active compound and inhibited 77% of CHKA activity. CK146 exhibited higher CHKA activity compared to ICL-CCIC-0019 (69% and 53%, respectively). Intriguingly, CK146 and CK145 displayed effective inhibition against phosphatidylinositol 4-kinase beta (PIK4CB) enzyme with 47% and 51% inhibition respectively. CK147 was inactive in the test panel.

### 3.7. [^18^F]D4-FCH Uptake In Vitro Study

**Figure 8:**
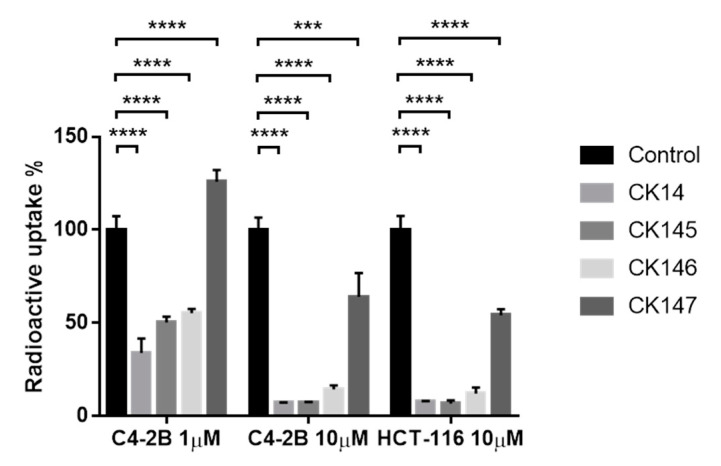
Uptake % of [^18^F]D4-FCH (0.74 MBq) in C4-2B and HCT-116 cells following 1-h incubation in the absence (Control; DMSO) or presence of CHKA inhibitors at 1 or 10 μM (*n* = 6; mean ± SD); *p* values via unpaired two-tailed *t*-test: *** *p* = 0.0001, **** *p* < 0.0001.

In C4-2B, a PSMA expressing cell line, CK14, CK145 and CK146 inhibited uptake of [^18^F]D4-FCH in a dose-dependent manner (Figure 8). At the dose of 1 μM, the compounds inhibited radio tracer uptake by approximately half (mean inhibition% by CK14, CK145 and CK146: 46 ± 1%). CK14 exhibited the best inhibitory potency in this study, inhibiting radiotracer uptake by 93 ± 5% when a higher dose was used (10 μM). In HCT-116 cells (PSMA negative), a similar inhibition trend by test compounds (10 μM) was observed (Figure 8). The average radiotracer uptake inhibition by 10 μM CK147 was 59 ± 7% across C4-2B and HCT-116 cell lines.

## 4. Discussion

**Figure 9:**
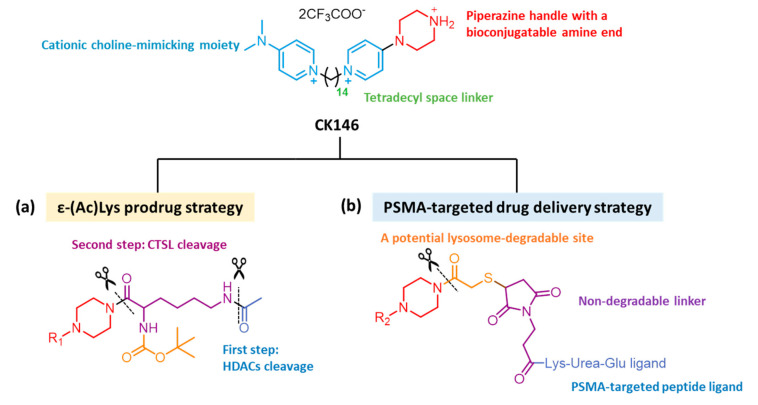
Design of two drug development strategies based on CK146. (**a**) ε-(Ac) Lys prodrug strategy; (**b**) PSMA-targeted drug delivery strategy.

Novel asymmetrical CHKA inhibitor CK146 was developed with the aim of maintaining the activity of ICL-CCIC-0019 pharmacophore but with the addition of a piperazine “reactive handle” (Figure 9), to allow further structural modifications. Previously described SAR of symmetrical CHKA inhibitors highlighted that the length of alkyl linker chain between the DMAP moieties impacted the potency of these molecules. A 14-carbon alkyl linker was thus incorporated into the structure of CK146 in an attempt to maintain the high potency of CK14 (IC_50_ against recombinant CHKA2: ICL-CCIC-0019: 270 nM; CK14: 150 nM) [26]. Compound CK146 was successfully synthesized in 4 steps in an overall yield of 54%. Despite the structural deviation of CK146 from ICL-CCIC-0019, good inhibitory activity against CHKA was maintained, essential for the successful development of a CHKA inhibitor with tuneable biological properties by further structural elaboration in prodrug and drug delivery strategies (Figure 9).

### 4.1. Prodrug Strategy

The upregulation of HDAC and CTSL enzymes in cancer has previously been exploited in a prodrug strategy whereby an ε-acetylated lysine linkage was selectively cleaved to release an active drug in a stepwise manner (Figure 9a) [47]. This strategy was adopted in this work and compound CK145, containing the ε-acetylated lysine linkage, was synthesized and evaluated as a prodrug of CK146. High expression of HDAC I class, including HDAC 1, 2, 3 and 8 subtypes, was thought to be necessary for converting CK145 into its active form. HDAC I subtype 1 may contribute the most to the first deacetylation step, activating the complete conversion of CK145 [47]. The enzymatic conversion of CK145 was analyzed by HPLC after incubation with HCT-116 cells. Despite HCT-116 cells showing high HDAC1 expression, CK146 and corresponding intermediates were not detected, indicating that the intracellular conversion of CK145 into CK146 was not initiated; this led to a difference in GI_50_ between the active scaffold (CK146) versus prodrug (CK145) in HepG2 and Caco-2 cells. HPLC analysis of cell lysates and SRB assay indicated that these compounds can be taken up into cells, likely via active internalization by choline transporters, despite their low-permeability revealed in the ADME studies. Rapid decomposition of CK145 was observed in the FaSSGF assay and metabolic stability assay due to Boc group deprotection at low pH (1.6) and peptide degradation mediated by liver microsomes.

To assess inhibitory activity against CHKA, compounds were screened against a panel of 15 human lipid kinases, where CK14 displayed the best CHKA activity (CK14: 77% inhibition of CHKA); CK146 displayed a better CHKA activity than ICL-CCIC-0019 (ICL-CCIC-0019: 53% inhibition of CHKA; CK146: 69% inhibition of CHKA). These results indicated the advantage of using a 14-carbon alkyl linker to achieve effective potency against CHKA, which is in agreement with the SAR of linker length documented by Trousil et al. (IC_50_ against recombinant CHKA2: ICL-CCIC-0019: 270 nM; CK14: 150 nM) [26]. CK145 offered similar CHKA inhibition to CK146 (63% and 69%, respectively), and this suggested that the prodrug strategy, whereby a bulky ε-(Ac)Lys structural modification had been made, was not able to mask the intrinsic activity of CK146. Another interesting observation in this study was the additional kinase activity towards PIK4CB (IC_50_: ~10 μM), a potential antimalarial and antiviral target, elicited by the modification of our CHKA inhibitors with a piperazine moiety [48,49]. In this regard, several CHKA inhibitors have been reported as capable of inhibiting the growth of parasite species *P. falciparum* (*P.f.*) through multiple mechanisms, including the inhibition of *P.f.* choline kinase and *P.f.* ethanolamine kinase [15,50]. CK146 was less selective to choline kinase. Kinase promiscuity can lower compound activity towards the desired target and induce adverse effects, both of which are undesirable characteristics for cancer therapeutics; however, it is noteworthy that the structure (CK146) presented here may have potential to be further modified into anti-parasite or antiviral drugs with a dual target-hitting PIK4CB and CHKA alike. This may warrant further investigation by researchers in the field, perhaps further supported by in silico docking studies against *P. falciparum* CHKA (pdb code: 6YXS) [51,52].

A ^18^F-radiolabeled choline analogue [^18^F]D4-FCH was utilized in this work to predict the in vitro choline uptake inhibition by compounds of interest. Parallel to the lipid kinase screening results, CK145 inhibited the uptake of [^18^F]D4-FCH in a similar trend to CK146, which suggested that the intact CK145 harbored CHKA activity.

### 4.2. Drug Delivery Strategy

A small-molecule peptide (Glu-Urea-Lys) with high affinity towards PSMA was conjugated to CK146 via a non-degradable linker, which gave rise to CK147, a proposed ligand-drug conjugate (LDC) for targeted PCa treatment (Figure 9b) [53]. CK147 was expected to bind to the PSMA receptor and internalize, followed by intracellular lysosomal degradation to release CK146; therefore, several PSMA-expressing prostate cell lines and PSMA-negative prostate cell lines were used in the following evaluation of the strategy [54]. The conjugate stability of CK147 in serum-containing medium was firstly investigated by HPLC over a period of 72 h which is in line with the incubation time used in the SRB proliferation assay (60% intact parent at 72 h). The antiproliferative activity (GI_50_) of CK147, in 7 prostate cell lines with differential PSMA expression and the HCT-116 colon cell line, was unrelated to PSMA expression; furthermore, all the tested cell lines were not sensitive to CK147. The observed poor antiproliferative activity of CK147 relative to CK146 implied that the two mechanisms driving the cellular uptake of CK147, (1) PSMA-driven internalization and (2) choline transporter-driven internalization, were not as efficient as expected. This led to subsequent experiments to investigate cellular uptake efficiency and in vitro metabolism by HPLC analysis of cell lysates and [^18^F]D4-FCH competitive study in the whole cells.

**Figure 10:**
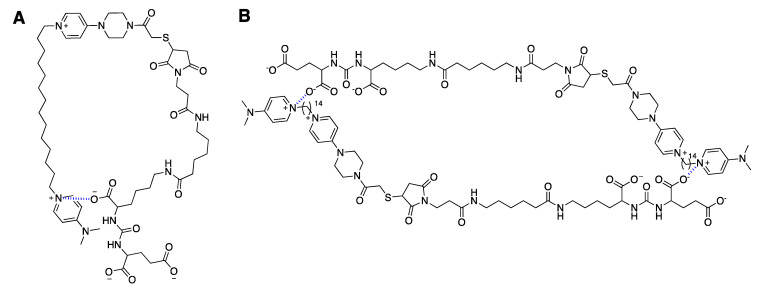
Proposed (**A**) intramolecular and (**B**) intermolecular electrostatic interactions (blue dashed line) for CK147 which inhibit activity towards the PSMA receptor and CHKA.

At physiological pH, CK147 contains two positive charges from the quaternary ammonium moieties, and three negative charges from the carboxylates of the Glu-Urea-Lys peptide. Although not zwitterionic due to the charge imbalance, we propose a putative mechanism where the positive quaternary ammonium charges may be masked by inter- or intramolecular electrostatic interactions which neutralize the charges and form unfavorable structural configurations capable of inhibiting both PSMA-binding and CHKA activity (Figure 10) [55]. The inactivity of CK147 observed in kinase screening was hypothesized to be related to the electrostatic neutralization; likely due to ionization of three carboxylate anions and two quaternary ammonium moieties at physiological pH. This hypothesis can also be supported by the significant increase in cellular uptake observed in the in vitro uptake study with CK148, the (OtBu)_3_-protected form of CK147. Electrostatic interactions may also explain the concentration reduction of CK147 observed in the stability study whilst no chemical decomposition was detected. The compound may have formed complexes with serum albumin over the time and then have been removed in the next protein precipitation step before MS analysis.

## 5. Conclusions

A novel derivative of ICL-CCIC-0019 bearing a piperazine handle (CK146) was successfully synthesized and retained biological activity towards CHKA; our work opens up the possibility for further structural elaboration of CK146, to explore the chemical space around this moiety and build up SAR to modulate biological and pharmacokinetic properties. Interestingly, the introduction of the piperazine structure to the scaffold seems to offer CHKA inhibitors additional activities towards PIK4CB, a potential antimalarial and antiviral target. In this work, we presented the successful transformation of CK146 into two drug development programs to produce targeted cancer therapies via prodrug strategy (CK145) and drug delivery strategy (CK147), although conversion to active components or targeted delivery was not achieved. Regardless, we have demonstrated the potential for modulating CHKA activity via piperazine functionalization and work in our laboratory will continue, including molecular modelling studies to understand the interactions between these molecules and the binding site of interest, to develop a suitable candidate for clinical translation.

## Figures and Tables

**Table 1:** Pharmacokinetic parameters of ICL-CCIC-0019, CK14, CK145, CK146 and CK147 from four ADME assays: kinetic solubility, human microsomal metabolic stability, permeability and efflux, and matrix stability.

Parameters		ICL-CCIC-0019	CK14	CK146	CK145	CK147
Kinetic Solubility	Solubility (μM)	161	154	158	178	186
Human Microsomal Metabolic Stability ^1^	Half-life (min)	>100	>100	>100	7.4	>100
	Clint (µL/min/mg)	<14	<14	<14	188	<14
	Predicted in vivo clearance (mL/min/kg)	<10	<10	<10	19	<10
	PCT_LBF %	<45	<45	<45	92	<45
	Remaining %	92	101	127	2	105
Permeability and Efflux	Papp A-B (×10^−6^ cm/s)	<0.1	<0.1	<0.1	<0.1	<0.1
	Papp B-A (×10^−6^ cm/s)	0.9	8.7	0.3	0.3	<0.1
	Efflux ratio (Papp B-A/Papp A-B)	>8.6	>87	>2.6	>2.8	No data ^1^
Matrix Stability	FaSSGF stability Half-life (h)	>10	>10	>10	9.2	>10
	Remaining %	117	95	92	74	97

^1^ Poorly permeable compound which prevented the calculation of an efflux ratio.

## Data Availability

Data available in a publicly accessible repository. The mRNA expression data presented in this study are openly available at Cancer Cell Line Encyclopedia (CCLE) Consortium, and Genomics of Drug Sensitivity in Cancer Consortium. 2015. Pharmacogenomic Agreement between Two Cancer Cell Line Data Sets. Nature 528 (7580):84–87. https://doi.org/10.1038/nature15736. The RNA-sequencing data utilized by CCLE are openly available on https://depmap.org/portal/download/.

## Supplementary Materials

The following are available online at https://www.mdpi.com/article/10.3390/pharmaceutics13071078/s1, Figures S1–S30: NMR and MS data of ICL-CCIC-0019, CK14 and compound 1–8, Figure S31: The purity check of CK147 by HPLC, Figure S32: The HPLC chromatograms of HCT-116 cell extracts after 1 h and 2 h incubation with/without CK145, Figure S33: MS characterization of the HCT-116 cell extracts, Figure S34: The HPLC chromatograms of CK146 detection in medium after treatment, Figure S35: Cellular uptake study of ICL-CCIC-0019, CK146, CK147 and CK148 in C4-2B cells using the medium with or without FCS, Figure S36–S45: MS characterization of the medium after treatment with ICL-CCIC-0019, CK146, CK147 and CK148 in C4-2B cells using the medium with or without FCS, Table S1: mRNA expression of HCT-116, HepG2, A549 and Caco-2, extracted from Cancer Cell Line Encyclopedia RNA sequencing database, Table S2: Inhibitory activity against HCT-116, A549, HepG2 and Caco-2 cancer cell lines (GI_50_) with ICL-CCIC-0019, CK146 and CK145, Table S3: Inhibitory activity against human 22Rv1, C4-2B, LNCap, R1AD1, R1-D567, PC3, PNT1A and HCT-116 cell lines (GI_50_) with ICL-CCIC-0019, CK146 and CK147.
