# Supplementary material for: Novel Non-Congeneric Derivatives of the Choline Kinase Alpha Inhibitor ICL-CCIC-0019
Source: Pharmaceutics. 2021 Jul 14;13(7):1078. doi: 10.3390/pharmaceutics13071078 (PMC8309005; doi:10.3390/pharmaceutics13071078)
Supplement: Supplementary file 1 [file pharmaceutics-13-01078-s001.zip › pharmaceutics-1289521-supplementary.pdf]

pharmaceutics

Supplementary materials: Novel Non-Congeneric Derivatives of the Choline Kinase Alpha Inhibitor ICL-CCIC-0019

Ning Wang, Diana Brickute, Marta Braga, Chris Barnes, Haonan Lu, Louis Allott and Eric O. Aboagye

Contents

1.0 Materials and methods 2

2.0 HPLC methods 2

3.0 Synthetic procedures 4

4.0 ^{1}H NMR, ^{13}C NMR, MS spectra and HPLC chromatograms 8

5.0 Antiproliferative assay 23

6.0 Cellular uptake and *in vitro* metabolism study 24

1.0. Materials and methods

The 1,14-bromotetradecane linker used in this project was purchased from Apollo Science Limited (Cheshire, UK). The small-molecule peptide ligand targeting PSMA attached to a maleimide group (($(OtBu)_3$ PSMA-maleimide)) **9** was provided by Creative Chemistry (Hampton, UK). All the other reagents and solvents were purchased from Sigma-Aldrich Chemical Co. (Gillingham, UK) and VWR Chemicals Co. (Lutterworth, UK), and used without further purification. Silica gel (230 - 400 mesh) for column chromatography was from Aldrich Chemical Co. (Dorset, UK). TLC was performed using silica gel on Al foils (254 nm) from Sigma-Aldrich (Gillingham, UK). All TLC plates were visualized by UV lamp (UVP, Upland, CA 91786, USA). Rotary evaporation was performed by BUCHI V-850 vacuum controllers (Suffolk, UK). BioChromato® spiral plug smart evaporator (Fujisawa, Japan) was used for compound drying and to remove DMF. NMR spectra were obtained using a Brucker Avance-400 spectrometer (Coventry, UK) and all the NMR data were analysed by MestReNova software. ^{1}H NMR were recorded at 400 MHz, and ^{13}C NMR were recorded at 101 MHz. MS (ES-ToF) were all obtained in the mass spectrometry centre in the chemistry department of Imperial College London (London, UK).

2.0. HPLC methods

The analytical and purification work of compound **5**, **6** and **7** was conducted at room temperature by a Waters™ 600 pump (Hertfordshire, UK) equipped with a DG 604 vacuum degasser (Alexandria, Minnesota, USA) and a Waters™ 2487 dual λ absorbance detector (Hertfordshire, UK). Data were analysed by the Empower software.

Method A

Instrument: Waters™ 600 pump (Hertfordshire, UK) equipped with a DG 604 vacuum degasser (Alexandria, Minnesota, USA) and a Waters™ 2487 dual λ absorbance detector (Hertfordshire, UK);

Stationary phase: Phenomenex® Luna 5μ C18 (2) 100Å 250 × 4.6 mm;

Gradient: mobile phase: A) water (0.2% formic acid), B) methanol; gradient: B (v/v): 5% to 40% in 20 min, 40% to 80% in 25 min, 80% to 95% in 15 min, 95% to 5% in 2 min;

Wavelengths: 254 nm and 294 nm;

Flow rate: 5 mL/min;

Injection volume: 2 mL;

Temperature: room temperature;

Method B

Instrument: Waters™ 600 pump (Hertfordshire, UK) equipped with a DG 604 vacuum degasser (Alexandria, Minnesota, USA) and a Waters™ 2487 dual λ absorbance detector (Hertfordshire, UK);

Stationary phase: Phenomenex® Luna 5μ C18 (2) 100Å 250 × 4.6 mm;

Gradient: mobile phase: A) water (0.2% formic acid), B) methanol; gradient: B (v/v): 5% to 40% in 5 min, 40% to 60% in 15 min, 60% to 80% in 10 min, 80% to 95% in 5 min, 95% to 5% in 2 min;

Wavelengths: 254 nm and 294 nm;

Flow rate: 4 mL/min;

Injection volume: 1 mL;

Temperature: room temperature;

Method C
Instrument: Waters™ 600 pump (Hertfordshire, UK) equipped with a DG 604 vacuum degasser (Alexandria, Minnesota, USA) and a Waters™ 2487 dual λ absorbance detector (Hertfordshire, UK);
Stationary phase: Phenomenex® Gemini 5μ C18 150 × 4.6 mm;
Gradient: mobile phase: A) water (0.2% formic acid), B) acetonitrile (0.2% formic acid); gradient: B (v/v): 5% to 95% in 16 min; 95% to 5% in 1 min; 5% for 3 min;
Wavelengths: 254 nm and 294 nm;
Flow rate: 1 mL/min;
Injection volume: 100 μL;
Temperature: room temperature.

To investigate the cellular uptake of ICL-CCIC-00019 and CK145 by HPLC analysis, external calibration curves were developed using five concentrations of ICL-CCIC-0019and CK145 (0.05, 0.1, 1, 5 and 7 μM) dissolved in HPLC mobile phase (water:acetonitrile:formic acid, 95:5:0.2 v/v/v). The corresponding peak was integrated to determine the area under the curve (AUC), which was plotted against the sample concentration. Linear regression was applied to the data ($R^2 = 0.999$), and the equation of the line was used to calculate unknown concentrations of ICL-CCIC-0019 and CK145. Using this procedure, the limit of detection (LOD) of the active form of the prodrug (CK146) was 1.382 μg/L which is calculated by this formula:

$$\text{Limit of Detection (LOD)} = \text{Limit of Detection (LOD)} = \frac{3 \times \text{Baseline noise} \times \text{Standard amount}}{\text{Sample signal height}}$$

Method D
Instrument: Agilent 1100 series (G1312A Pump, G1322A Degasser, G1314A Variable Wavelength Detector) (Stockport, UK);
Stationary phase: Phenomenex® Gemini 5μ C18 150 × 4.6 mm;
Gradient: mobile phase: A) was water (0.2% formic acid), B) acetonitrile; gradient: B (v/v): 5% to 95% in 15 min; 95% to 5% in 1 min; 5% for 3 min;
Wavelengths: 254 nm;
Flow rate: 1 mL/min;
Injection volume: 100 μL;
Temperature: room temperature.

To quantify unknown concentrations of CK147 in the *in vitro* studies by HPLC analysis, internal calibration curves were established, where ICL-CCIC-0019 was exploited as an internal standard (IS). Chromatograms were analysed using Laura Software (LabLogic, Sheffield, UK) to integrate peaks to determine the AUC. The internal standard (IS) ratio was calculated for each sample by dividing the peak area of CK147 by that of the IS, and the concentration of CK147 was determined by comparing the IS ratio to a concomitantly performed standard curve. Standard curves, containing CK147 concentrations of 7.5, 15, 30, 60 and 120 μg/mL and a fixed concentration of ICL-CCIC-0019 (20 μg/mL) in water:acetonitrile:formic acid (95:5:0.2, v/v/v), were constructed by plotting the IS ratio versus the known concentration of CK147 in each sample. Linear regression was applied to the data ($R^2 = 0.9999$), and the equation of the line was used to calculate unknown concentrations of CK147.

3.0 Synthetic procedures:

1,1'-(dodecane-1,12-diyl)bis(4-(dimethylamino)pyridin-1-ium) bromide (ICL-CCIC-0019)

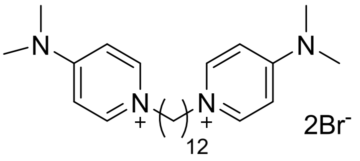

To a round bottom flask was added 4-dimethylaminopyridine (1 mmol, 122 mg) and 1,12-dibromododecane (0.5 mmol, 164 mg) in 2-butanone (10 mL). The reaction was stirred at 110 °C for 3 h and a white precipitate formed. The precipitate was filtered, washed with 2-butanone (3 × 10 mL), diethyl ether (2 × 10 mL) and subsequently dried *in vacuo*. The title compound was purified by recrystallisation with ethanol and 2-butanone to give a white solid (189 mg, yield = 84%). ^{1}H NMR (400 MHz, DMSO-d_6) δ 1.14 - 1.29 (16H, m), 1.69 - 1.78 (4H, m), 3.18 (12H, s), 4.14 (4H, t, *J* = 8.0 Hz), 7.03 (4H, d, *J* = 8.0 Hz), 8.30 (4H, d, *J* = 8.0 Hz); ^{13}C NMR (101 MHz, $CDCl_3$) δ 26.20, 28.90, 29.20, 31.40, 31.50, 40.90, 58.70, 108.90, 143.10, 156.70; HRMS (ES-ToF) m/z ($[M\text{-}Br^-]^{2+}$): calcd: 206.1778, found: 206.1767.

1,1'-(tetradecane-1,14-diyl)bis(4-(dimethylamino)pyridin-1-ium) bromide (CK14)

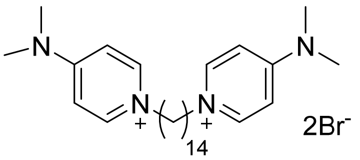

To a round bottom flask was added 4-dimethylaminopyridine (2 mmol, 244 mg) and 1,14-bromotetradecane (1 mmol, 356 mg) in 2-butanone (20 mL). The reaction was stirred at 110 °C for 3 h and a white precipitate formed. The precipitate was filtered, washed with 2-butanone (3 × 20 mL) and diethyl ether (2 × 20 mL) and subsequently dried *in vacuo*. The title compound was purified by recrystallisation with ethanol and 2-butanone to give a white solid (381 mg, yield = 80%).^{1}H NMR (400 MHz, DMSO- d_6) δ 1.14 - 1.31 (20H, m), 1.69 - 1.79 (4H, m), 3.19 (12H, s), 4.14 (4H, t, *J* = 4.0), 7.03 (4H, d, *J* = 8.0 Hz), 8.30 (4H, d, *J* = 8.0 Hz); ^{13}C NMR (101 MHz, $CDCl_3$) δ 26.30, 28.10, 28.80, 28.90, 29.50, 31.10, 40.90, 58.80, 108.90, 143.00, 156.70; MS (ES-ToF) m/z ($[M\text{-}Br^-]^{2+}$): calcd: 220.2, found: 220.2.

tert-butyl 4-(pyridin-4-yl)piperazine-1-carboxylate (1)

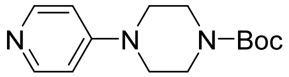

To a round bottom flask was added 4-piperazinopyridine (1 mmol, 163 mg), di-tert-butyl dicarbonate (1.5 mmol, 327 mg) and a catalytical amount of DMAP in anhydrous DMF (10 mL) and TEA (107 μL) before being purged with nitrogen. The reaction was stirred at room temperature for 16 h before concentration *in vacuo*. The title compound was purified by flash chromatography (silica; ethyl acetate (0.3% TEA, v/v); RF value: 0.18) to give a white solid (247 mg, yield = 94%). ^{1}H NMR (400 MHz, $CDCl_3$) δ 1.39 (9H, s), 3.20 (4H, t, *J* = 4.8 Hz), 3.46 (4H, t, *J* = 4.8 Hz), 6.55 (2H, d, *J* = 6.8 Hz), 8.18 (2H, d, *J* = 6.4 Hz); ^{13}C NMR (101 MHz, $CDCl_3$) δ 28.30, 45.60, 54.50, 79.99, 108.38, 150.15, 154.40, 154.57; MS (ES-ToF) m/z ($[M+H]^+$): calcd: 264.1707, found 264.1725.

1-(bromomethyl)-4-(dimethylamino)pyridin-1-ium bromide (2)

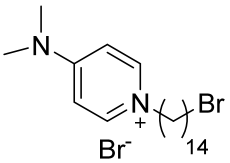

To a round bottom flask was added 4-dimethylaminopyridine (0.5 mmol, 61 mg) and 1,14-bromotetradecane (1 mmol, 356 mg) in 2-butanone (10 mL). The reaction was stirred at 110 °C for 3 h and a white precipitate formed. The reaction mixture was poured into diethyl ether (200 mL) and the precipitate was filtered and washed with 2-butanone (3 × 10 mL), diethyl ether (2 × 10 mL) and subsequently dried *in vacuo* to give the title compound as a white solid (152 mg, yield = 64%). ^{1}H NMR (400 MHz, $CDCl_3$) δ 1.17 - 1.50 (20H, m), 1.81 - 1.95 (4H, m), 3.28 (6H, s), 3.40 (2H, t, *J* = 8.0 Hz), 4.35 (2H, t, *J* = 8.0 Hz), 7.03 (2H, d, *J* = 8.0 Hz), 8.48 (2H, d, *J* = 8.0 Hz); ^{13}C NMR (101 MHz, $CDCl_3$) δ 26.20, 28.30, 28.90, 29.20, 29.51, 29.56, 29.60, 29.64, 29.70, 31.30, 32.90, 34.10, 40.60, 58.70, 108.50, 142.50, 156.40; MS (ES-ToF) m/z([M-Br$^-$]$^+$): calcd: 399.2192, found: 399.2208.

4-(4-(tert-butoxycarbonyl)piperazin-1-yl)-1-(14-(4-(dimethylamino)pyridin-1-ium-1-yl)tetradecyl)pyridin-1-ium bromide (3)

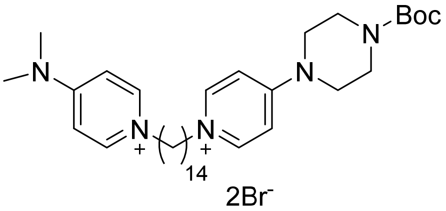

To a sealed tube **1** (0.25 mmol, 66 mg) and **2** (0.25 mmol, 120 mg) were added in dry acetonitrile (6 mL). The reaction was stirred at 110 °C for 72 h. The reaction mixture was cooled down to the ambient temperature and concentrated *in vacuo* to give product as a pale yellow solid (180 mg, yield = 97%). ^{1}H NMR (400 MHz, MeOD-d$_4$) δ 1.13 - 1.33 (20H, m), 1.37 - 1.44 (9H, s), 1.72 - 1.84 (4H, m), 3.17 (6H, s), 3.50 - 3.60 (4H, m), 3.63 - 3.70 (4H, m), 4 - 4.15 (4H, m), 6.91 (2H, d, *J* = 7.6 Hz), 7.08 (2H, d, *J* = 7.6 Hz), 8.07 (2H, d, *J* = 7.6 Hz), 8.13 (2H, d, *J* = 7.6 Hz), 8.46 (2H, s); ^{13}C NMR (101 MHz, MeOD-d$_4$) δ 27.20, 28.59, 30.20, 30.60, 30.68, 30.76, 31.93, 40.25, 46.92, 58.94, 59.10, 81.90, 108.90, 109.48, 143.03, 143.72, 156.15, 157.54, 157.90; MS (ES-ToF) m/z ([M-Br$^-$]$^{2+}$): calcd: 290.7329, found 290.7342.

4-(dimethylamino)-1-(14-(4-(piperazin-1-yl)pyridin-1-ium-1-yl)tetradecyl)pyridin-1-ium hydrogen 2,2,2-trifluoro-acetate (4) (CK146)

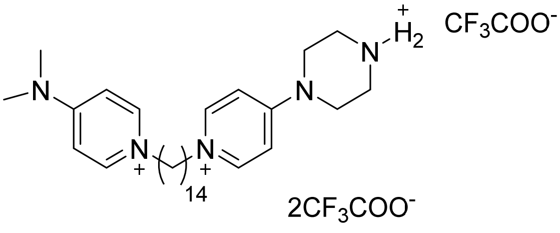

To a dry round bottom flask was added **3** (0.34 mmol, 253 mg) in dry MeCN (3 mL) with addition of TFA (3 mL). The reaction was stirred at room temperature for 16 h before being concentrated to dryness *in vacuo* (265 mg, yield = 93%). ^{1}H NMR (400 MHz, MeOD-d$_4$) δ 1.16 - 1.32 (20H, m), 1.73 - 1.85 (4H, m), 3.17 (6H, s), 3.50 - 3.60 (4H, m), 3.26 - 3.35 (4H, m), 3.83 - 3.92 (4H, m), 4.04 - 4.18 (4H, m), 6.91 (2H, d, *J* = 7.6 Hz), 7.19 (2H, d, *J* = 7.6 Hz), 8.07 (2H, d, *J* = 7.6 Hz), 8.00 - 8.15 (2H, s), 8.23 (2H, d, *J* = 7.6 Hz); ^{13}C NMR (101 MHz, MeOD-d$_4$) δ 27.28, 30.28, 30.67, 30.75, 30.83, 32.01, 40.34, 44.33, 45.26, 59.05, 59.43, 108.99, 110.03, 143.11, 144.20, 157.76, 157.99. MS (ES-ToF) m/z ([M-CF_3COO^-]$^{2+}$): calcd: 240.7067, found 240.7062.

4-(4-(N⁶-acetyl-N²-(tert-butoxycarbonyl)lysyl)piperazin-1-yl)-1-(14-(4-(dimethylamino)pyridin-1-ium-1-yl)tetradecyl)pyridin-1-ium formate (5) (CK145)

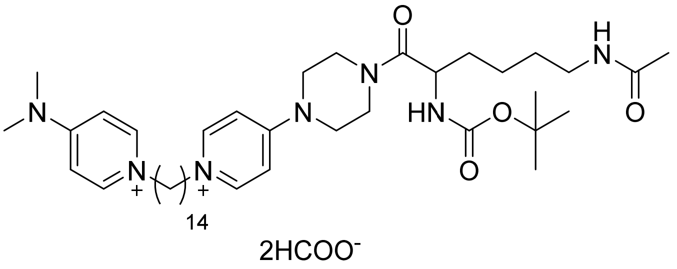

The crude of **5** was provided by Creative Chemistry (Hampton, UK). The compound was synthesized from the starting materials of α-Boc-Lys(Ɛ-Ac)-OH and EDC, HOBt·H_2O, DIEA, DMF. The crude was then analyzed by HPLC and MS to confirm the identity of the product. The title compound was purified by semi-preparative HPLC from the crude (HPLC method A, 307 mg, Yield = 37.5%). ^{1}H NMR (400 MHz, MeOD-d_4) δ 1.2 - 1.41 (24H, m), 1.44 (9H, s), 1.49 - 1.57 (2H, m), 1.59 - 1.76 (2H, m), 1.78 - 1.90 (4H, m), 1.93 (3H, s), 3.05 - 3.22 (2H, m), 3.25 (6H, s), 3.64 - 4.05 (4H, m), 4.12 - 4.26 (4H, m), 4.45 - 4.56 (1H, m), 6.99 (2H, d, *J* = 7.6 Hz), 7.18 (2H, d, *J* = 7.2 Hz), 8.16 (2H, d, *J* = 7.6 Hz), 8.24 (2H, d, *J* = 7.2 Hz), 8.56 (2H, s); ^{13}C NMR (101 MHz, MeOD-d_4) δ 22.61, 23.97, 27.18, 28.71, 30.00, 30.18, 30.58, 30.66, 30.73, 31.91, 32.44, 40.00, 40.26, 42.24, 45.09, 46.51, 46.92, 51.76, 58.93, 59.16, 80.63, 108.89, 109.49, 143.01, 143.73, 157.53, 157.87, 170.17, 173.21, 173.69; MS (ES-ToF) m/z ([M-HCOO$^-$]$^{2+}$): calcd: 375.7857, found 375.7766.

4,4'-((2,2'-disulfanediylbis(acetyl))bis(piperazine-4,1-diyl))bis(1-(14-(4-(dimethylamino)pyridin-1-ium-1-yl)tetradecyl)pyridin-1-ium) formate (6)

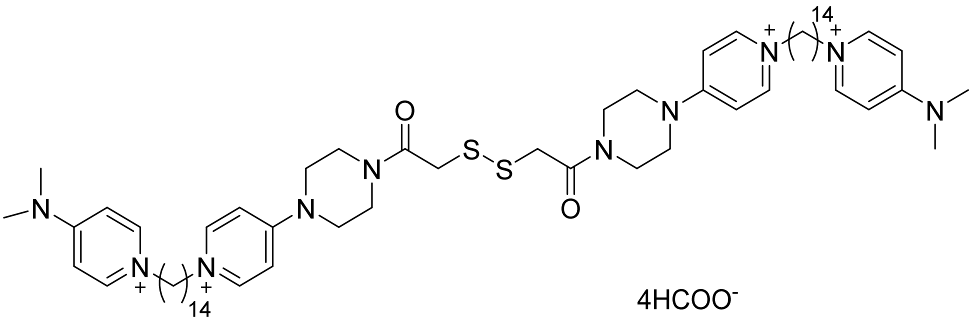

To a round bottom flask was added compound **4** (hydrochloride salt form, 0.27 mmol, 180 mg), dithiodiglycolic acid (0.133 mmol, 24 mg), EDC•HCl (0.27 mmol, 51 mg), HOBt (0.27 mmol, 36 mg), and DIPEA (0.27 mmol, 47 μL) in anhydrous DMF (6 mL) before being purged with nitrogen. The reaction was stirred at room temperature for 72 h before being dried by smart evaporator. The title compound was purified by semi-preparative HPLC purification (See HPLC method B) to give a clear beige solid (70 mg, yield = 48%).^{1}H NMR (400 MHz, MeOD-d_4) δ 1.07 - 1.35 (40H, m), 1.7 - 1.87 (8H, m), 3.16 (12H, s), 3.41 - 3.99 (19H, m), 4.03 - 4.22 (9H, m), 6.91 (4H, d, *J* = 7 Hz), 7.11 (4H, s), 8.08 (4H, d, *J* = 7.1 Hz), 8.17 (4H, s); ^{13}C NMR (101 MHz, MeOD-d_4) δ 27.16, 30.15, 30.55, 30.63, 30.70, 31.89, 31.91, 42.29, 45.84, 46.41, 46.97, 58.93, 59.15, 108.88, 109.52, 142.99, 143.74, 157.49, 157.84, 166.74, 169.55. MS (ES-ToF) m/z ([M-HCOO$^-$]$^{4+}$): calcd: 277.1941, found 277.1960.

4-(4-(2-((1-(7,11-bis(tert-butoxycarbonyl)-2,2-dimethyl-4,9,17,24-tetraoxo-3-oxa-8,10,16,23-tetraazahexacosan-26-yl)-2,5-dioxopyrrolidin-3-yl)thio)acetyl)piperazin-1-yl)-1-(14-(4-(dimethylamino)pyridin-1-ium-1-yl)tetradecyl)pyridin-1-ium formate (7) (CK148)

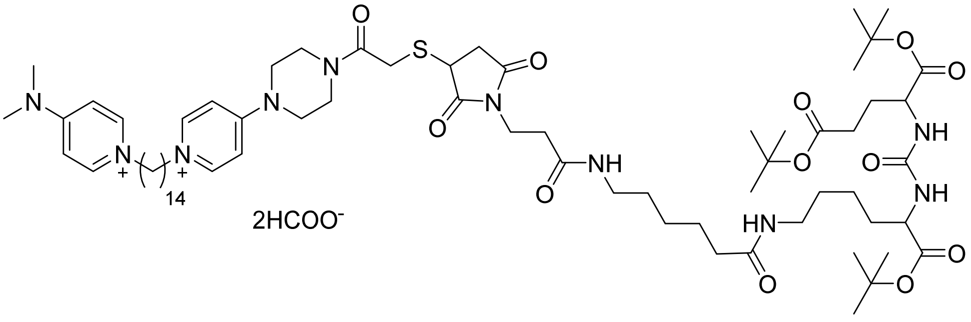

To a round bottom flask was added compound **6** (0.054 mmol, 70 mg) and tris(2-carboxyethyl)phosphine (TCEP) hydrochloride (0.27 mmol, 78 mg) in 4 mL of degassed Tris-HCl buffer (pH 7.3) before being purged with nitrogen. The pH of the reaction was adjusted to 7 using 1 M NaOH and stirred at room temperature for 2 h. Then PSMA-Maleimide (Creative Chemistry, Hampton, UK) (0.14 mmol, 102 mg) was predissolved in DMF and Tris-HCl buffer solution (4 mL) was dropwise added into the reaction mixture. The reaction was stirred at room temperature under nitrogen atmosphere for 16 h before being dried by smart evaporator. The title compound was purified by semi-preparative HPLC purification (See HPLC method B) to give a clear yellow solid (85 mg, yield = 56%). ^{1}H NMR (400 MHz, MeOD-d$_4$) δ 1.16 - 1.31 (20H, m), 1.31 - 1.37 (3H, m), 1.37 - 1.41 (27H, m), 1.43 - 2.06 (14H, m), 2.08 - 2.15 (2H, t, *J* = 7.4 Hz), 2.16 - 2.46 (4H, m), 2.56 - 2.67 (2H, m), 2.95 - 3.18 (4H, m), 3.19 (6H, s), 3.60 - 3.90 (12H, m), 3.93 - 4.21 (7H, m), 6.94 (2H, d, *J* = 7.4 Hz), 7.14 (2H, d, *J* = 6.5 Hz), 8.10 (2H, d, *J* = 7.4 Hz), 8.19 (2H, d, *J* = 6.6 Hz). ^{13}C NMR (101 MHz, MeOD-d$_4$) δ 23.99, 26.67, 27.18, 27.56, 28.99, 29.94, 29.99, 30.18, 30.58, 30.66, 30.73, 31.92, 32.48, 33.00, 33.14, 34.78, 36.05, 36.79, 36.99, 40.06, 40.27, 40.31, 41.05, 42.25, 45.79, 46.36, 46.83, 54.18, 54.90, 58.93, 59.15, 81.71, 82.52, 82.76, 108.89, 109.52, 143.01, 143.76, 157.52, 157.86, 159.92, 169.80, 172.70, 173.43, 173.70, 173.89, 175.95, 176.37, 177.76. MS (ES-ToF) m/z ([M-HCOO$^-$]$^{2+}$): calcd: 653.4169, found 653.4164.

4-(dimethylamino)-1-(14-(4-(4-(2-((2,5-dioxo-1-(1,3,7-tricarboxy-5,13,20-trioxo-4,6,12,19-tetraazadocosan-22-yl)pyrrolidin-3-yl)thio)acetyl)piperazin-1-yl)pyridin-1-ium-1-yl)tetradecyl)pyridin-1-ium 2,2,2-trifluoroacetate (8) (CK147)

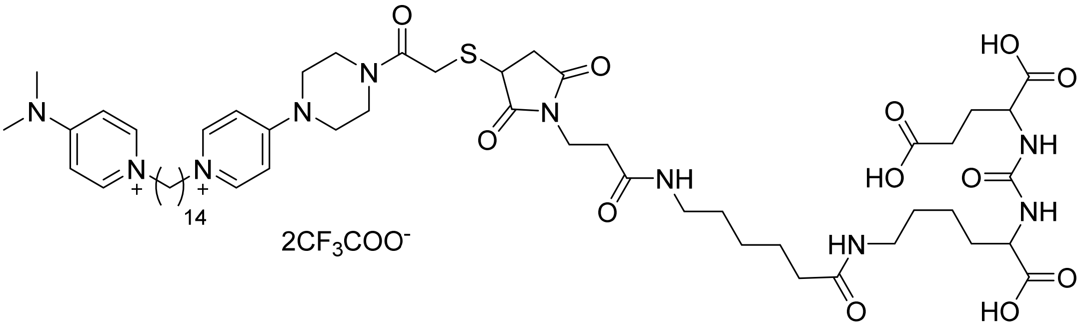

To a round bottom flask was added compound **7** (0.0138 mmol, 18 mg) and TFA (1.32 mmol, 100 μL), in anhydrous DCM (500 μL) before being purged with nitrogen. The reaction was stirred at room temperature for 16 h before being dried *in vacuo*. The title compound was used without further purification as a clear pale yellow solid (16 mg, yield = 25%). ^{1}H NMR (400 MHz, MeOD-d$_4$) δ 1.13 - 1.25 (20H, m), 1.27 - 1.84 (17H, m), 191 - 2.21 (4H, m), 2.24 - 2.39 (4H, m), 2.5 - 2.67 (2H, m), 2.95 - 3.10 (4H, m), 3.12 (6H, s), 3.55 - 3.82 (12H, m), 3.93 - 4.23 (7H, m), 6.86 (2H, d, *J* = 7.9 Hz), 7.06 (2H, d, *J* = 7.8 Hz), 8.02 (2H, d, *J* = 7.8 Hz), 8.11 (2H, d, *J* = 7.7 Hz).; ^{13}C NMR (101 MHz, MeOD-d$_4$) δ 23.97, 26.66, 27.21, 27.50, 28.88, 29.93, 30.21, 30.61, 30.69, 30.77, 31.95, 32.97, 33.16, 34.82, 35.99, 36.81, 36.96, 40.07, 40.25, 40.33, 41.06, 42.27, 45.79, 46.35, 46.82, 53.52, 54.00, 58.95, 59.17, 108.88, 109.51, 143.01, 143.75, 157.53, 157.88, 160.12, 169.86, 172.82, 175.80, 176.08, 176.36, 176.43, 177.79. MS (ES-ToF) m/z ([M-CF$_3$COO$^-$]$^{2+}$): calcd: 569.3230, found 569.3251.

4.0 ^{1}H NMR, ^{13}C NMR, MS spectra and HPLC chromatograms

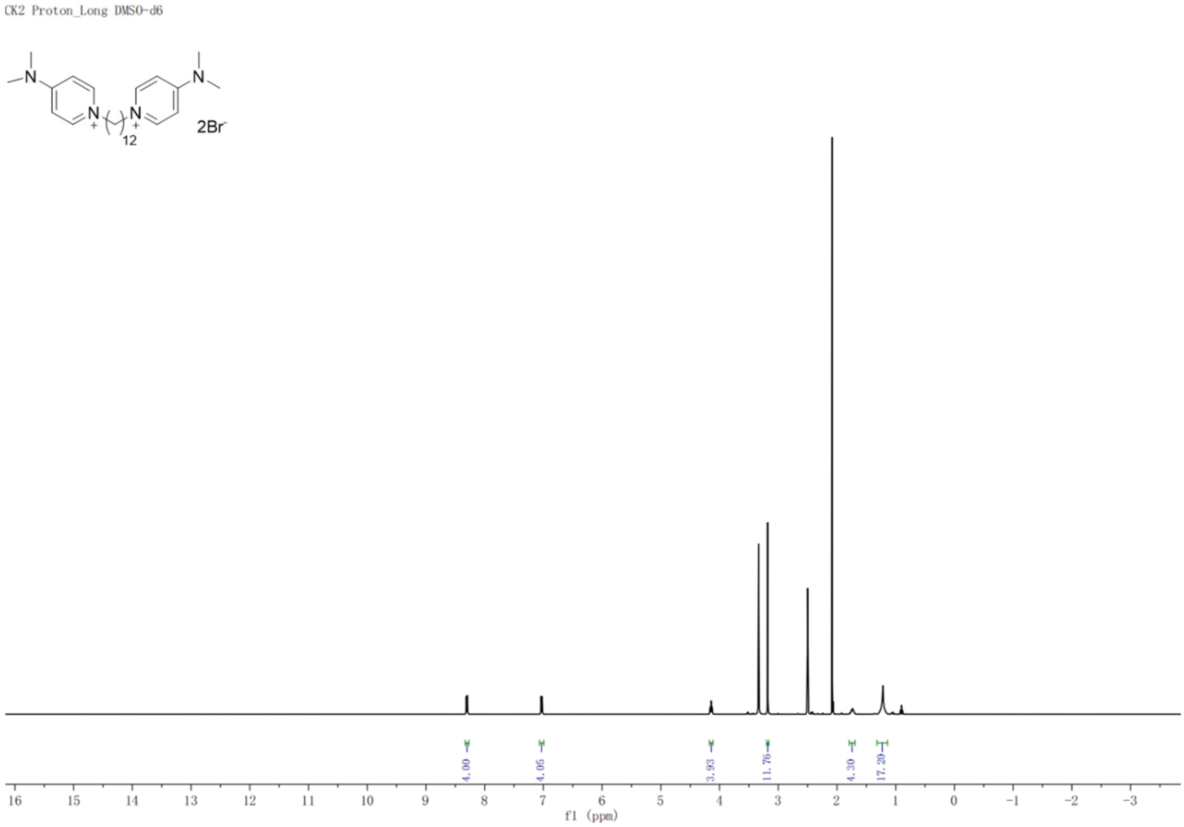

Figure S1. ^{1}H NMR of ICL-CCIC-0019.

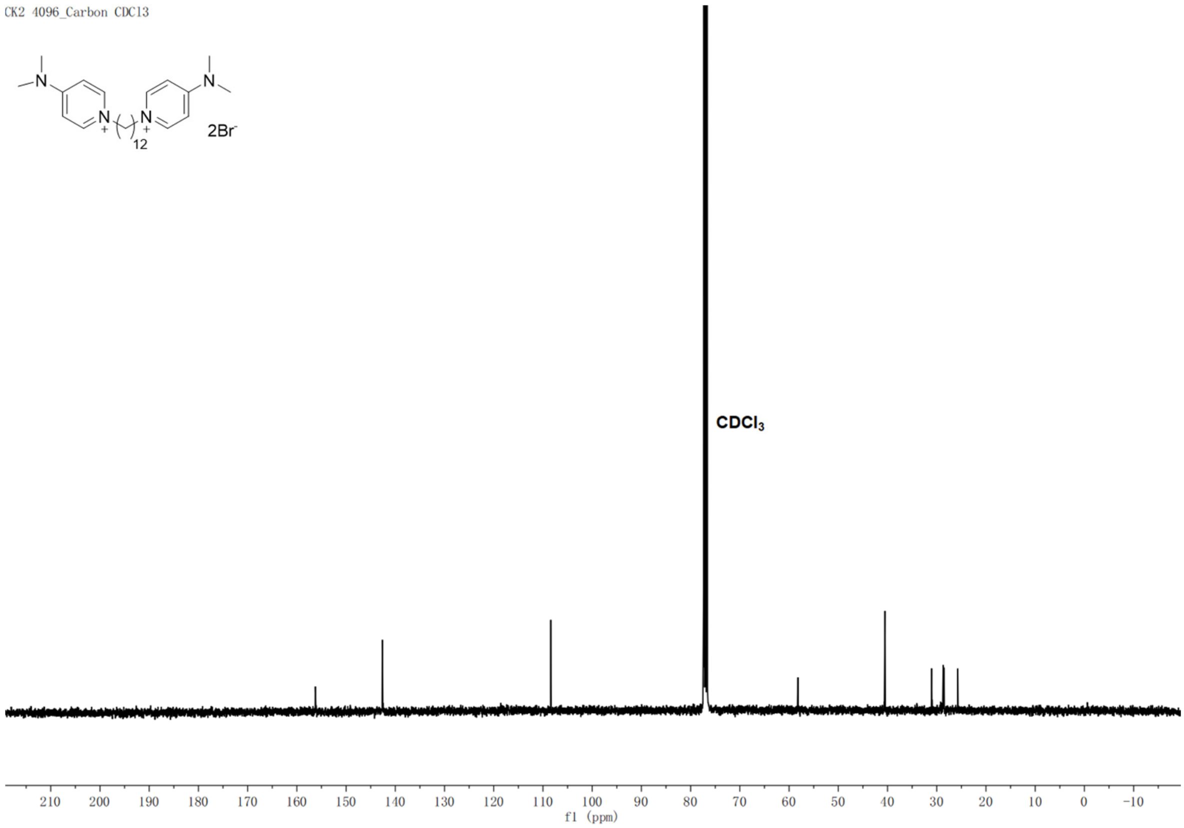

Figure S2. ^{13}C NMR of ICL-CCIC-0019.

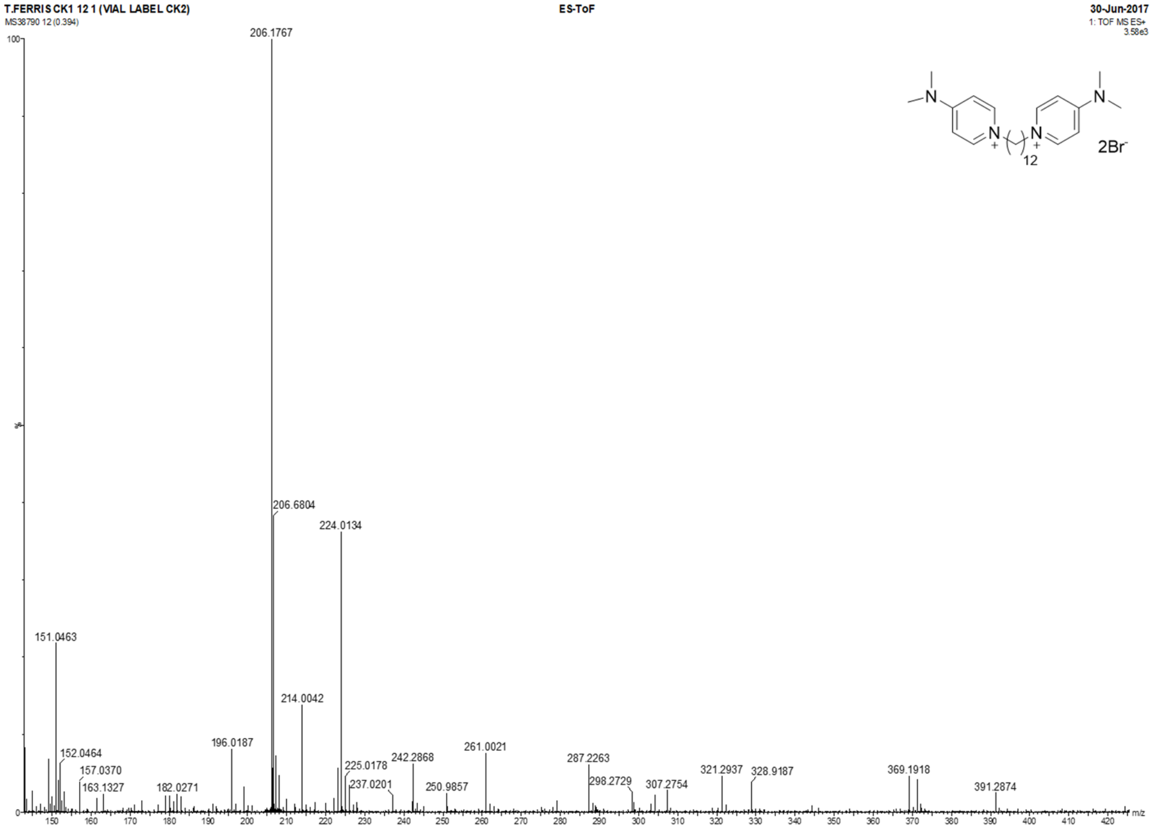

Figure S3. MS of ICL-CCIC-0019.

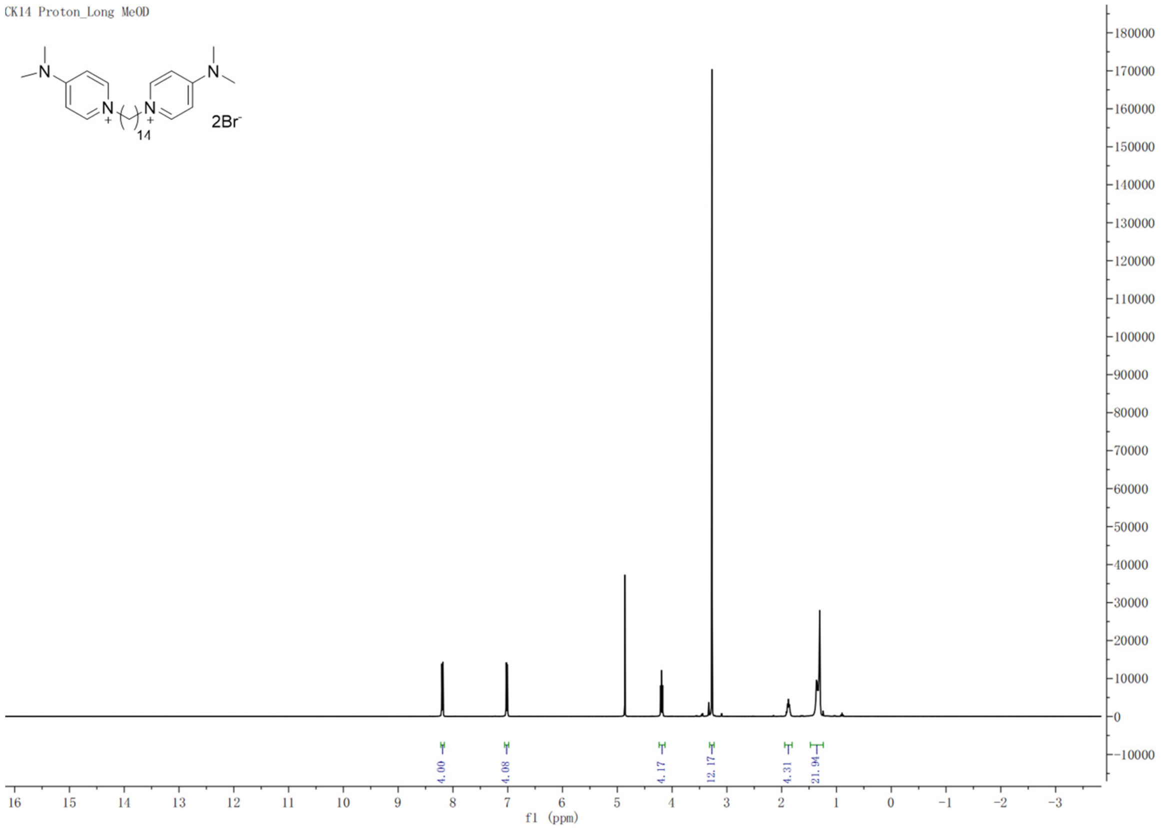

Figure S4. ^{1}H NMR of CK14.

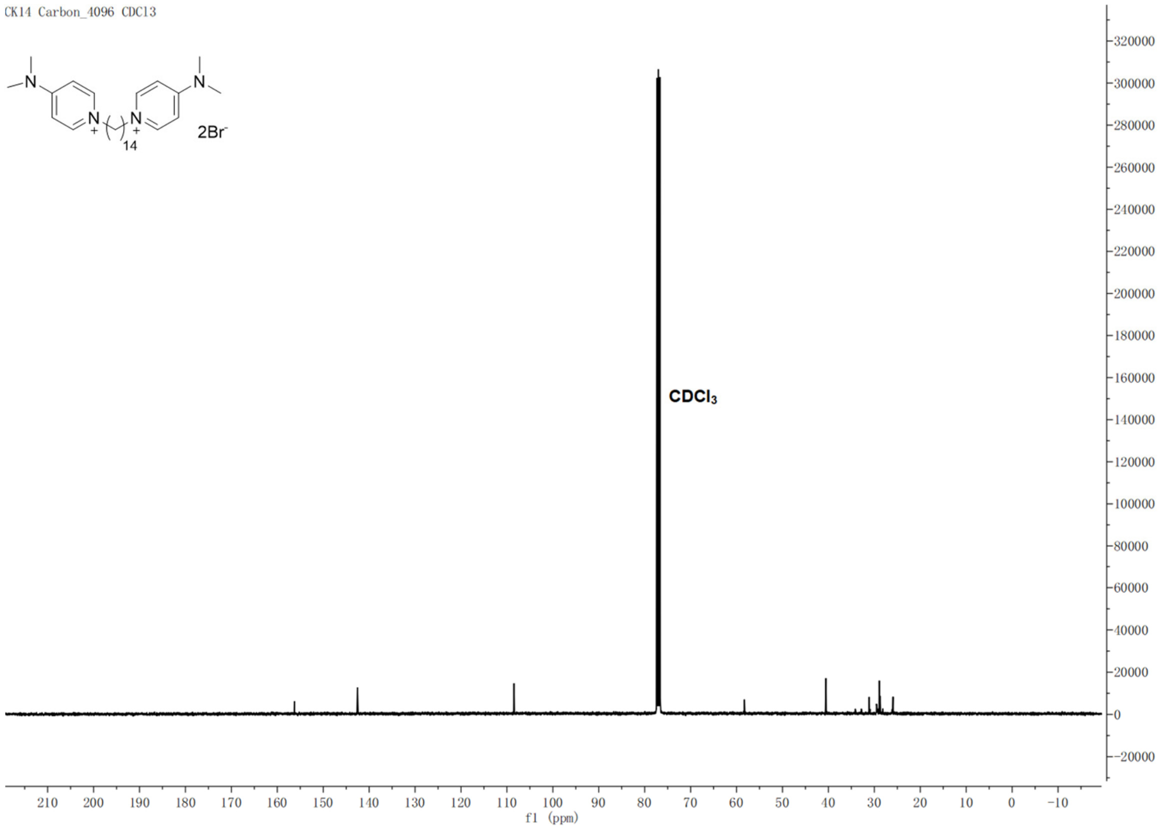

Figure S5. ^{13}C NMR of CK14.

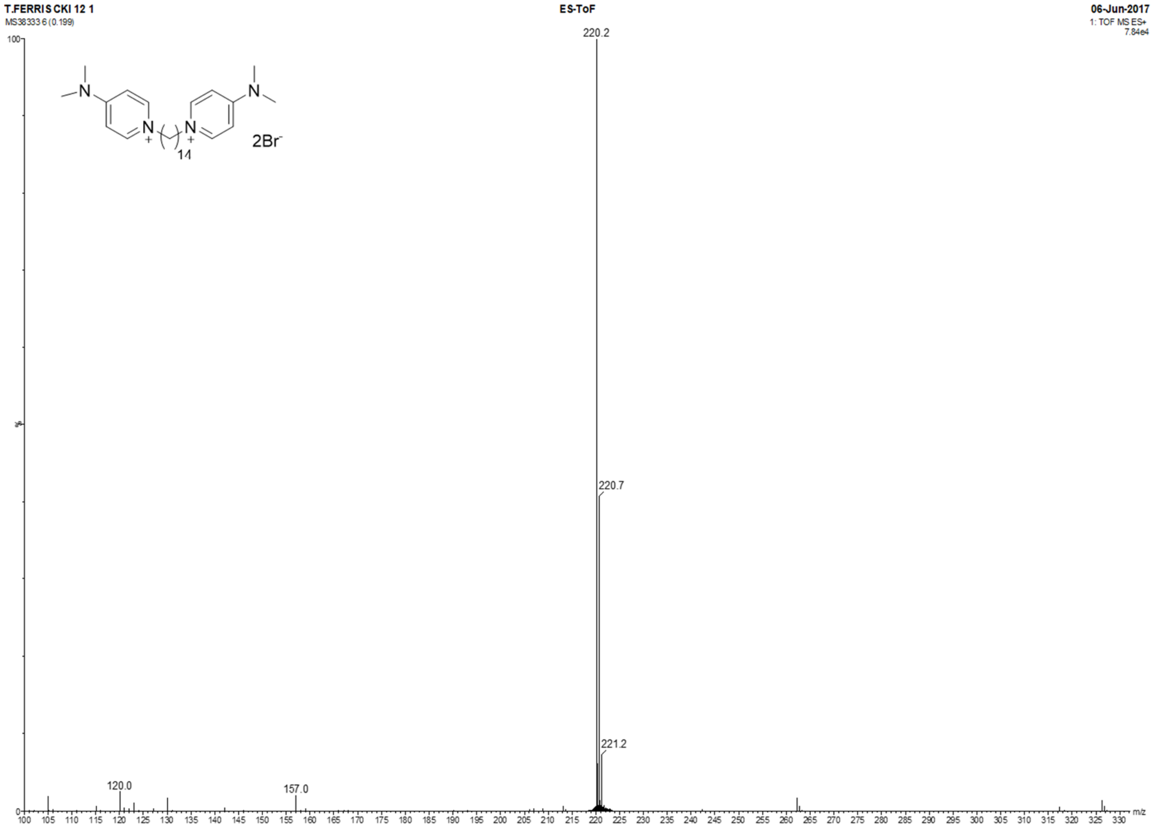

Figure S6. MS of CK14.

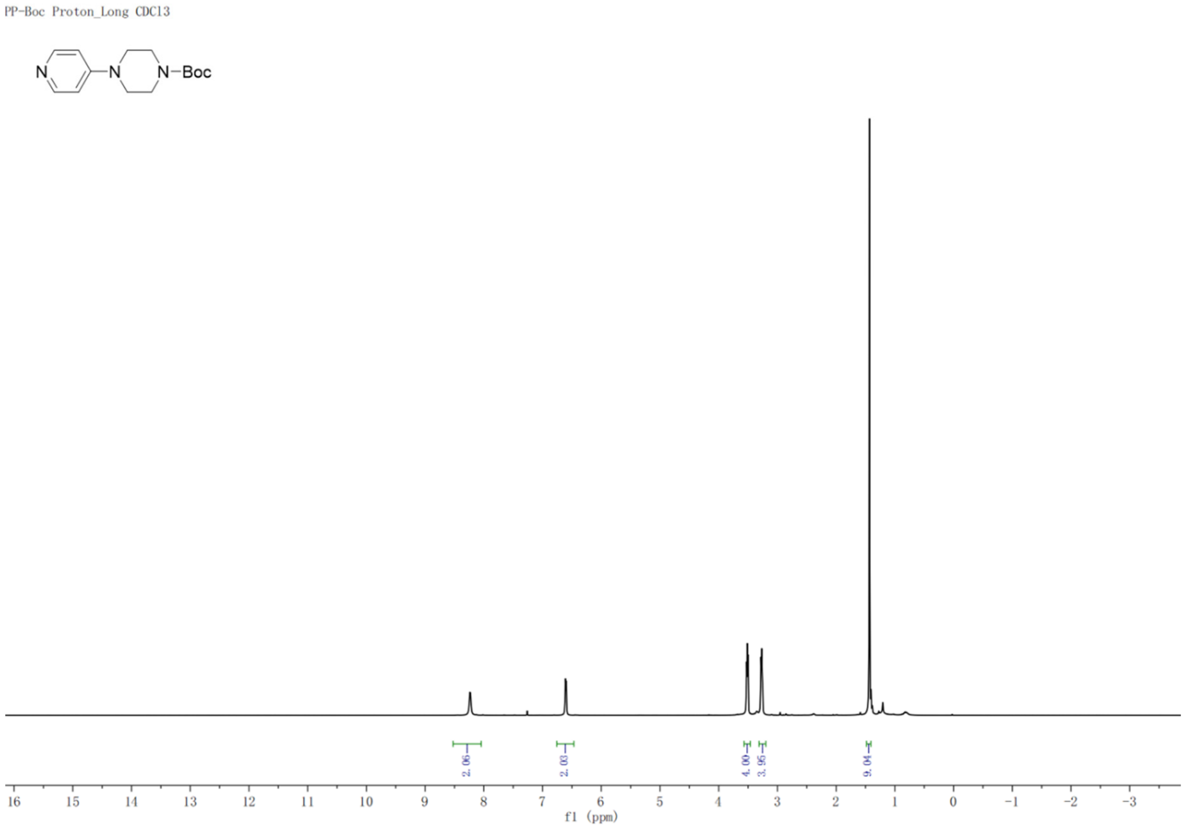

Figure S7. ^{1}H NMR of **1**.

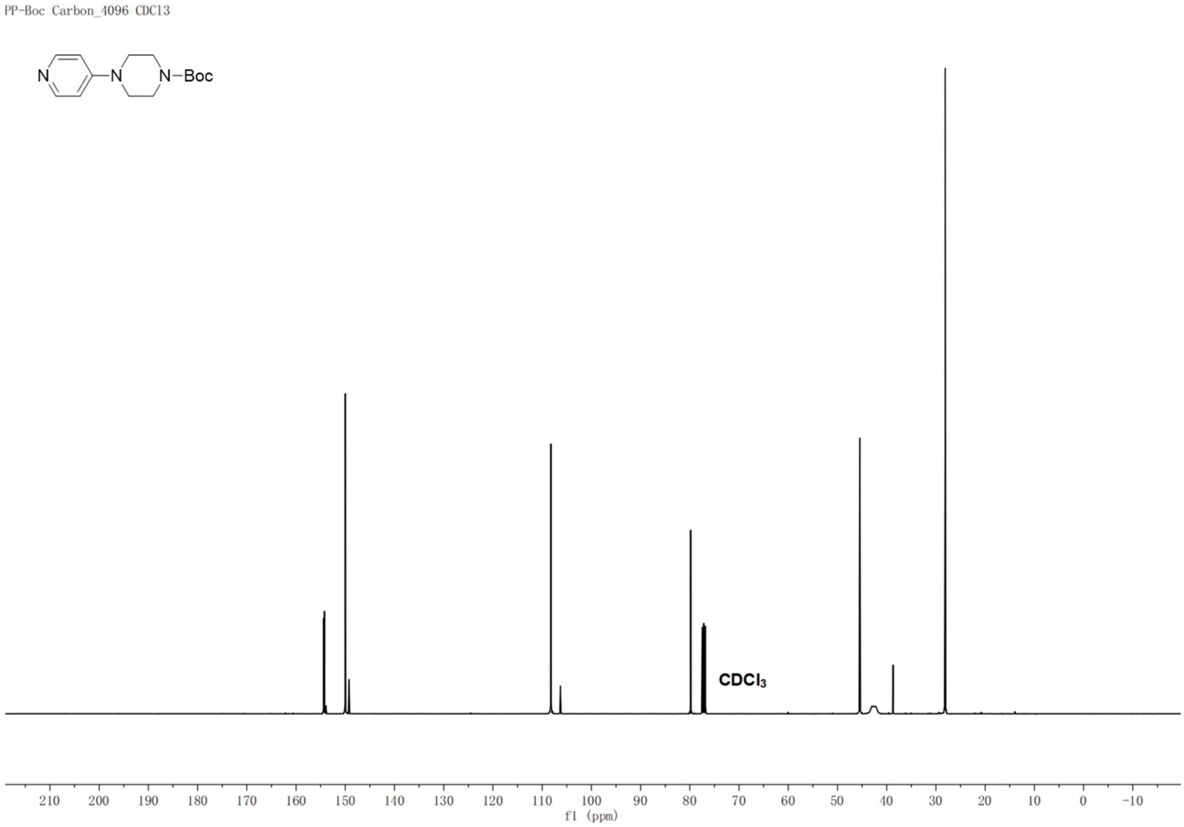

Figure S8. ^{13}C NMR of **1**.

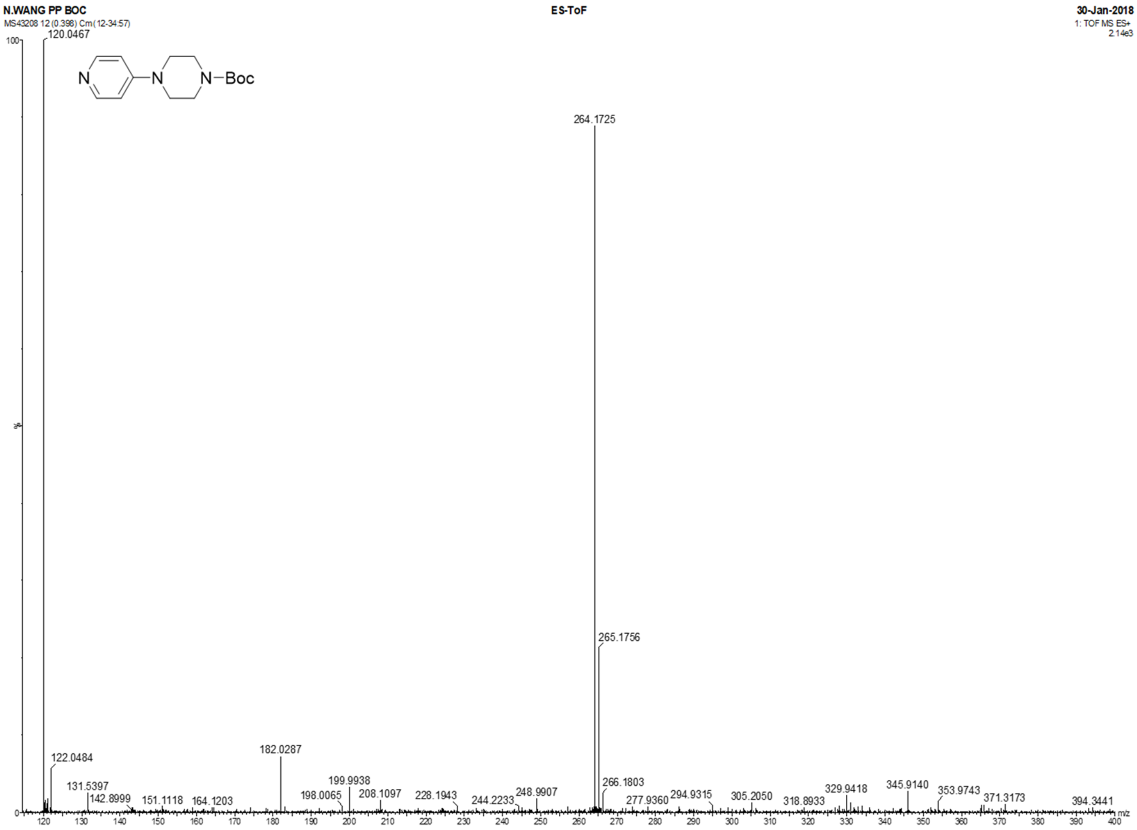

Figure S9. MS of **1**.

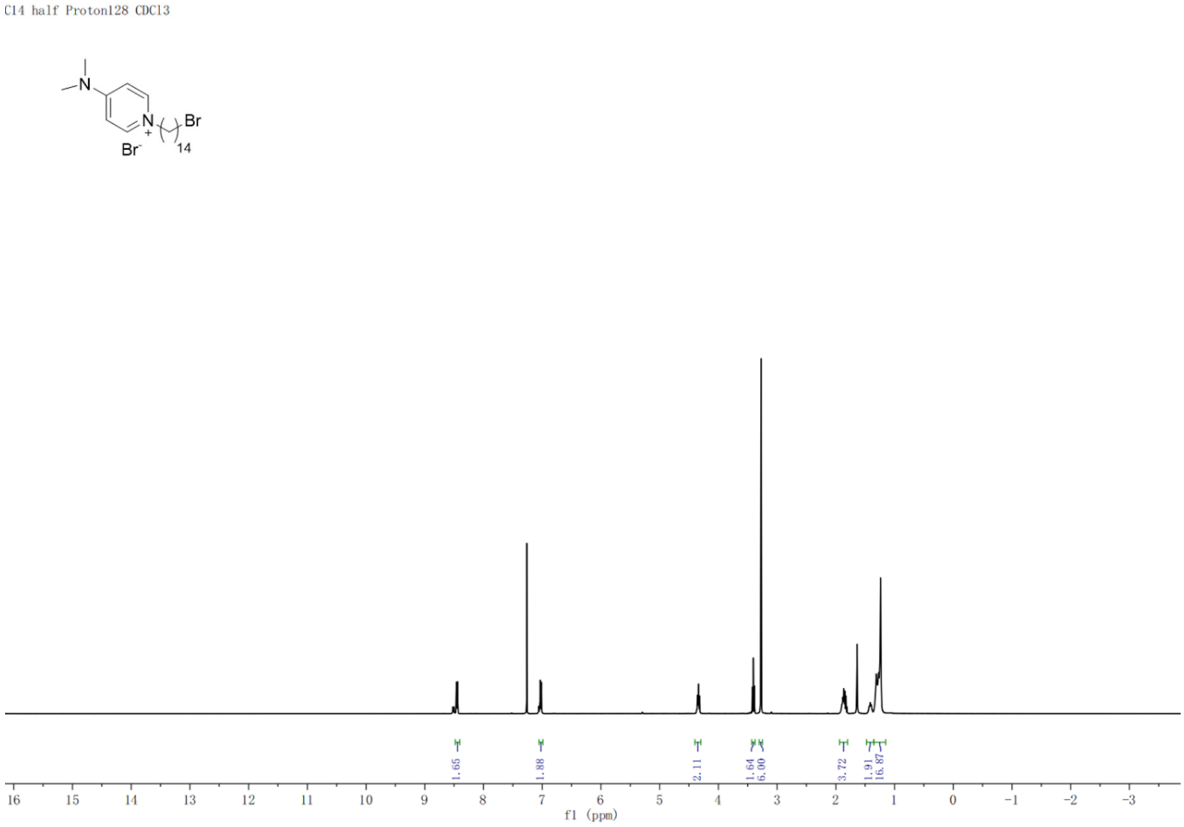

Figure S10. ^{1}H NMR of **2**.

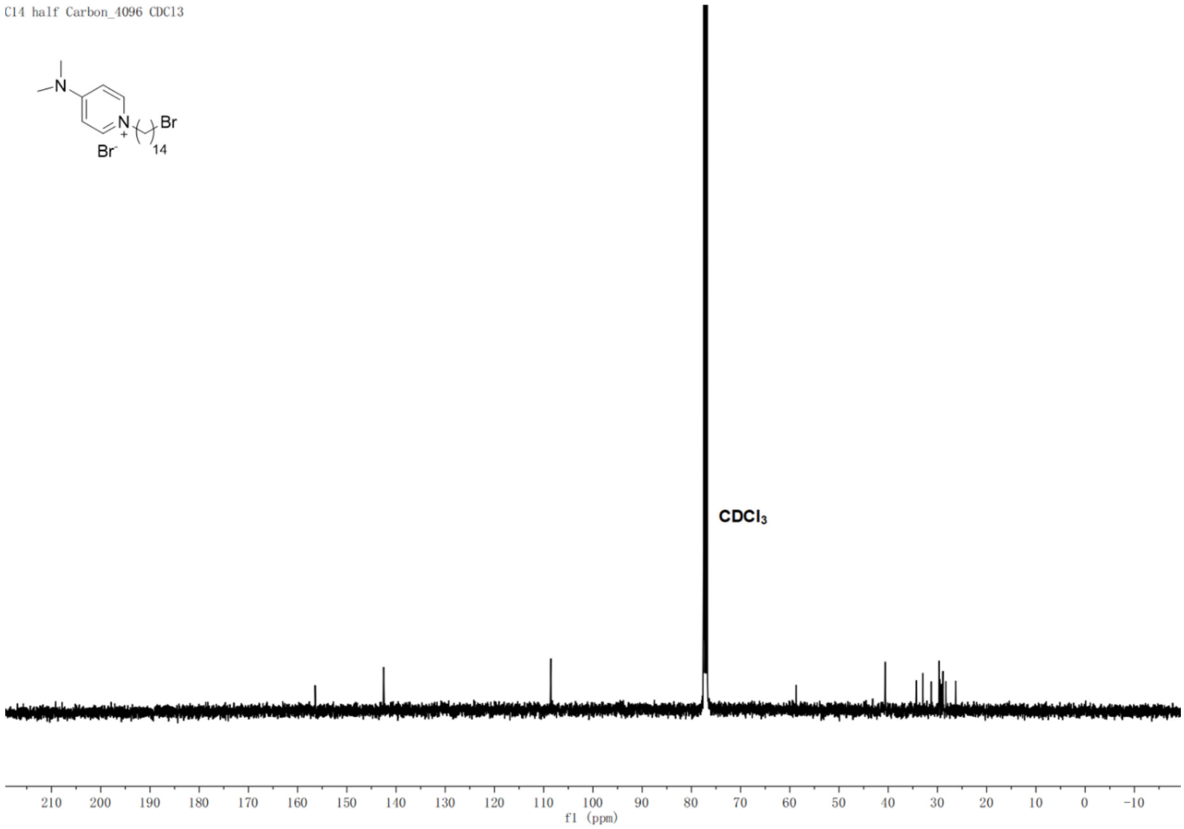

Figure S11. ^{13}C NMR of **2**.

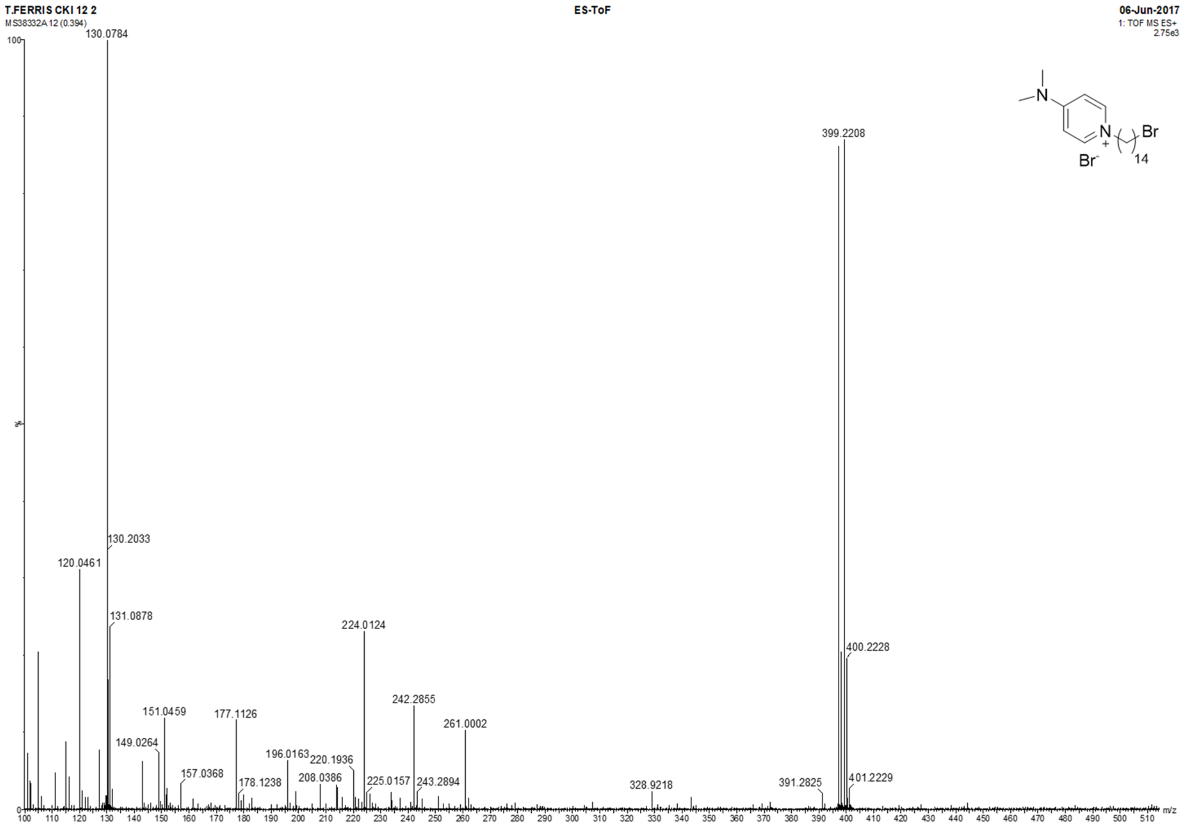

Figure S12. MS of **2**.

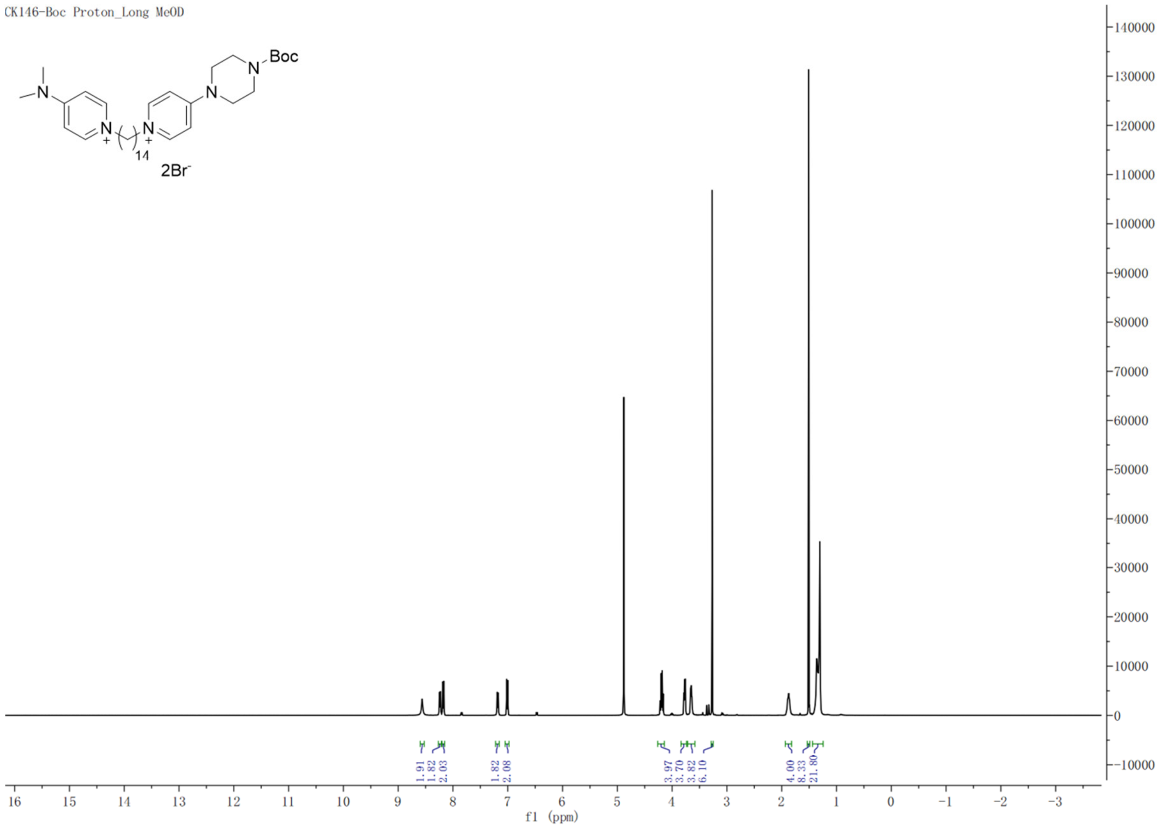

Figure S13. ^{1}H NMR of **3**.

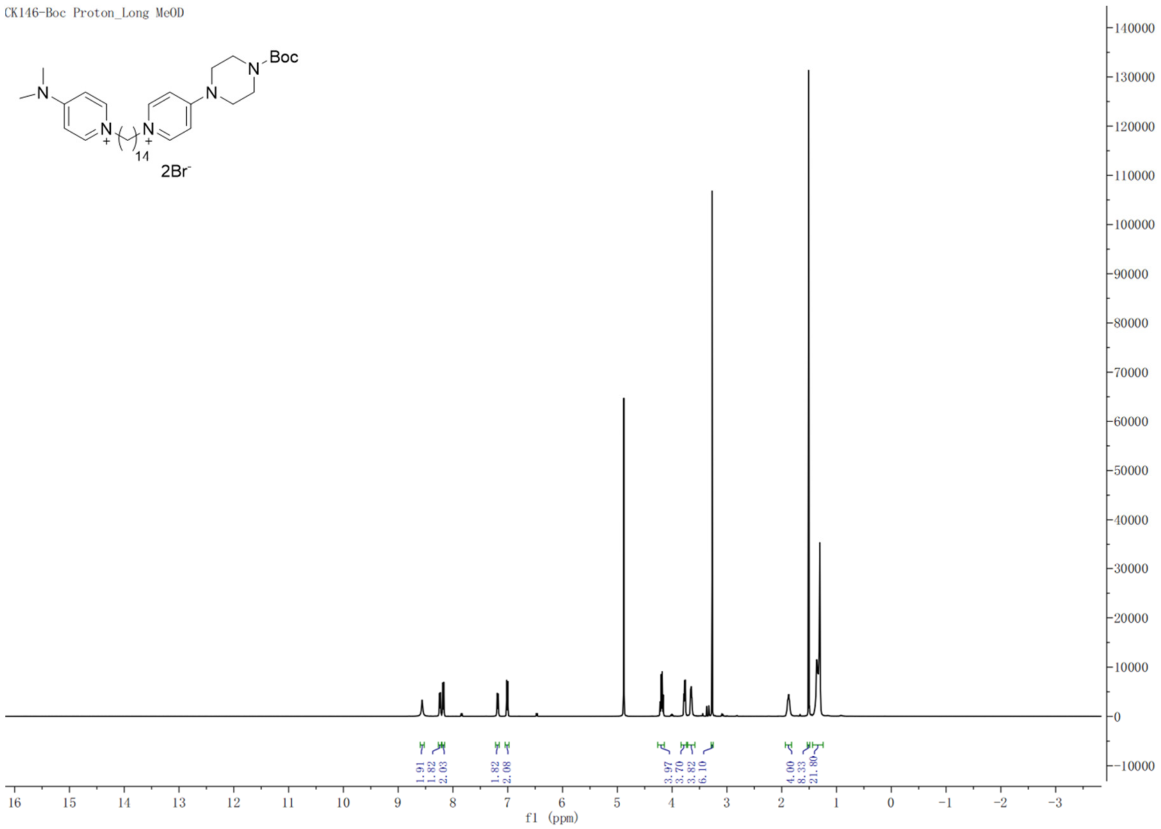

Figure S14. ^{13}C NMR of **3**.

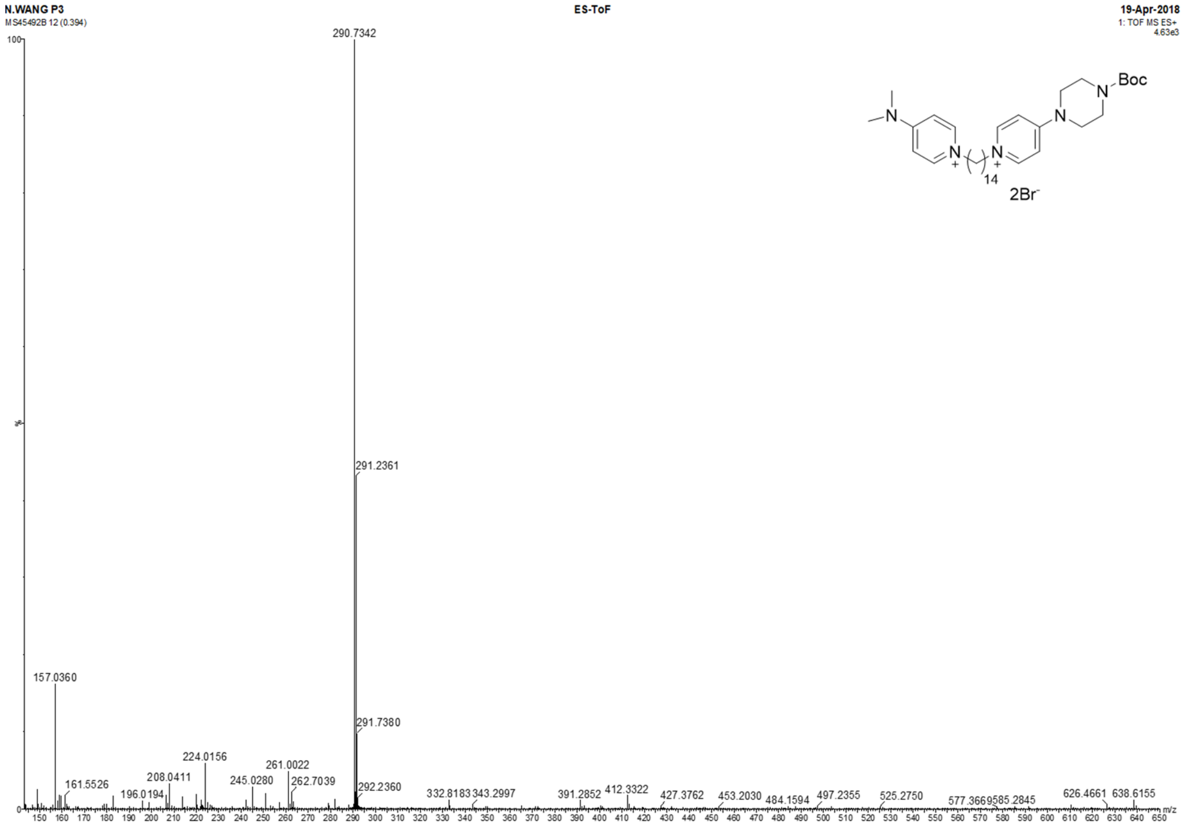

Figure S15. MS of **3**.

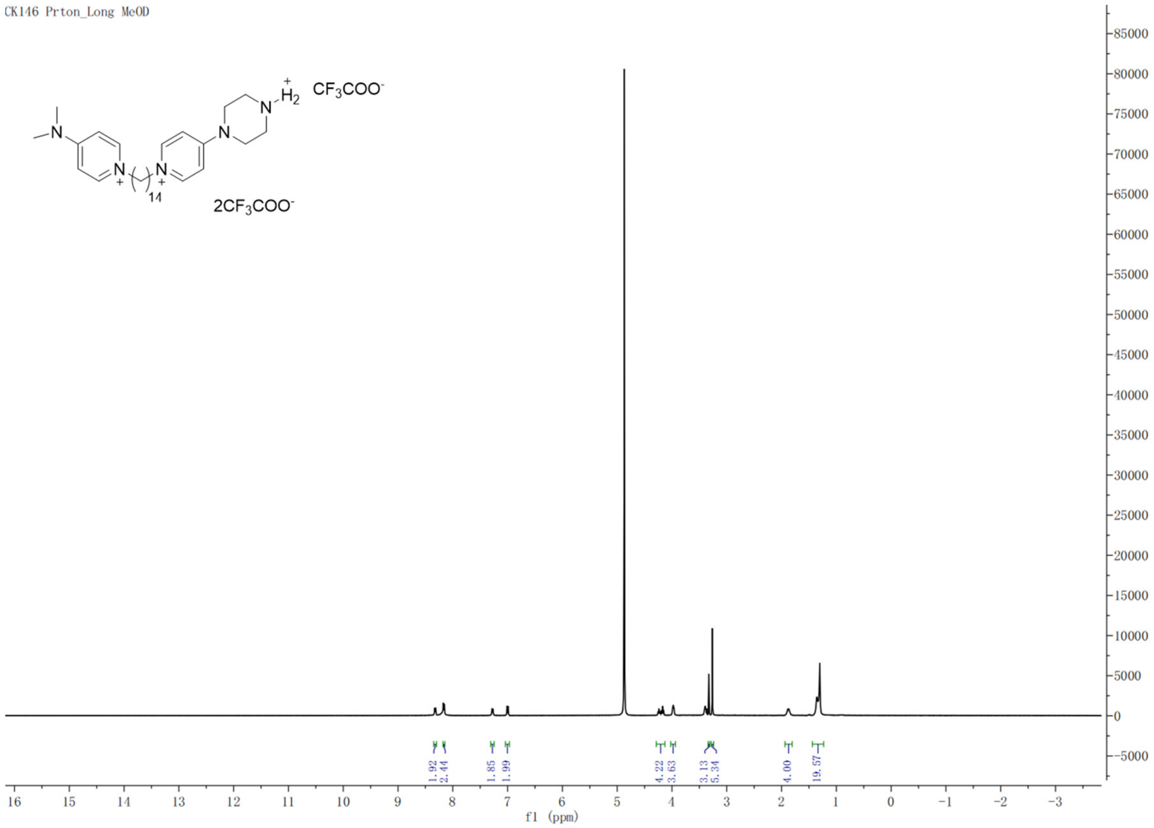

Figure S16. ^{1}H NMR of **4**.

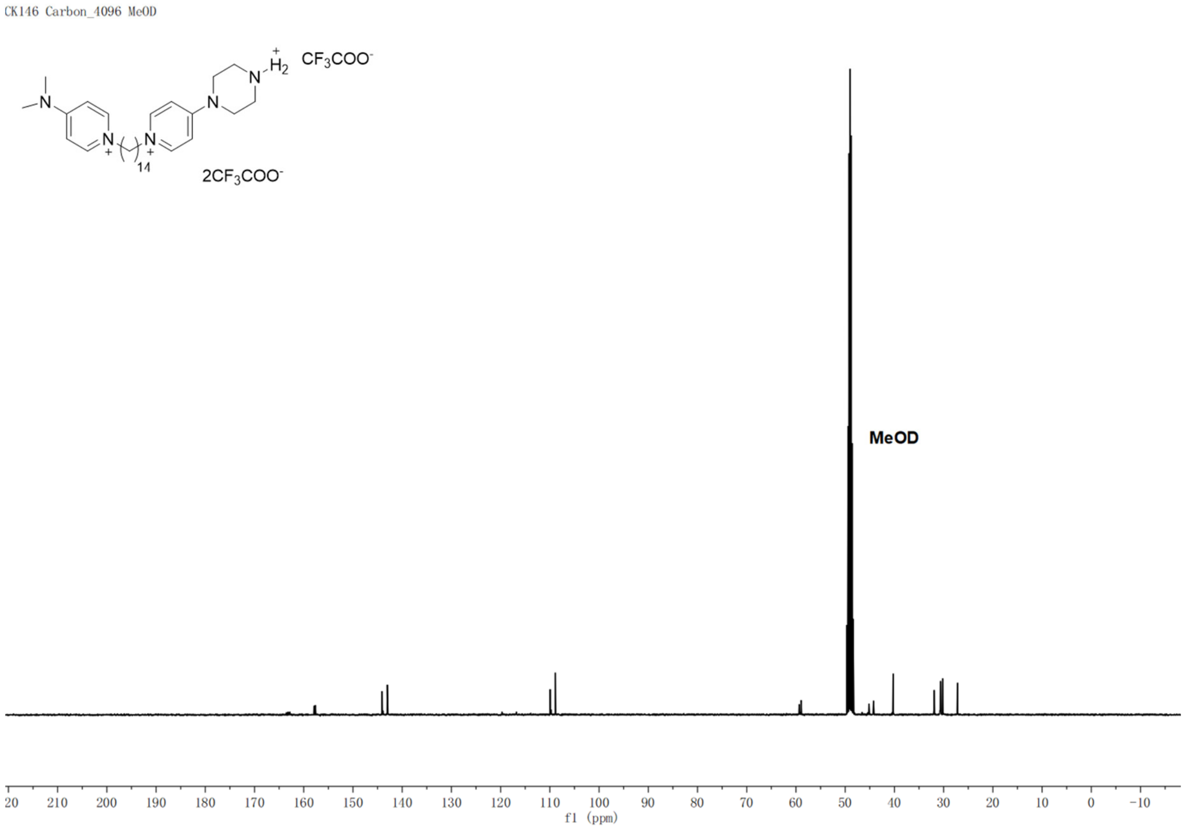

Figure S17. ^{13}C NMR of **4**.

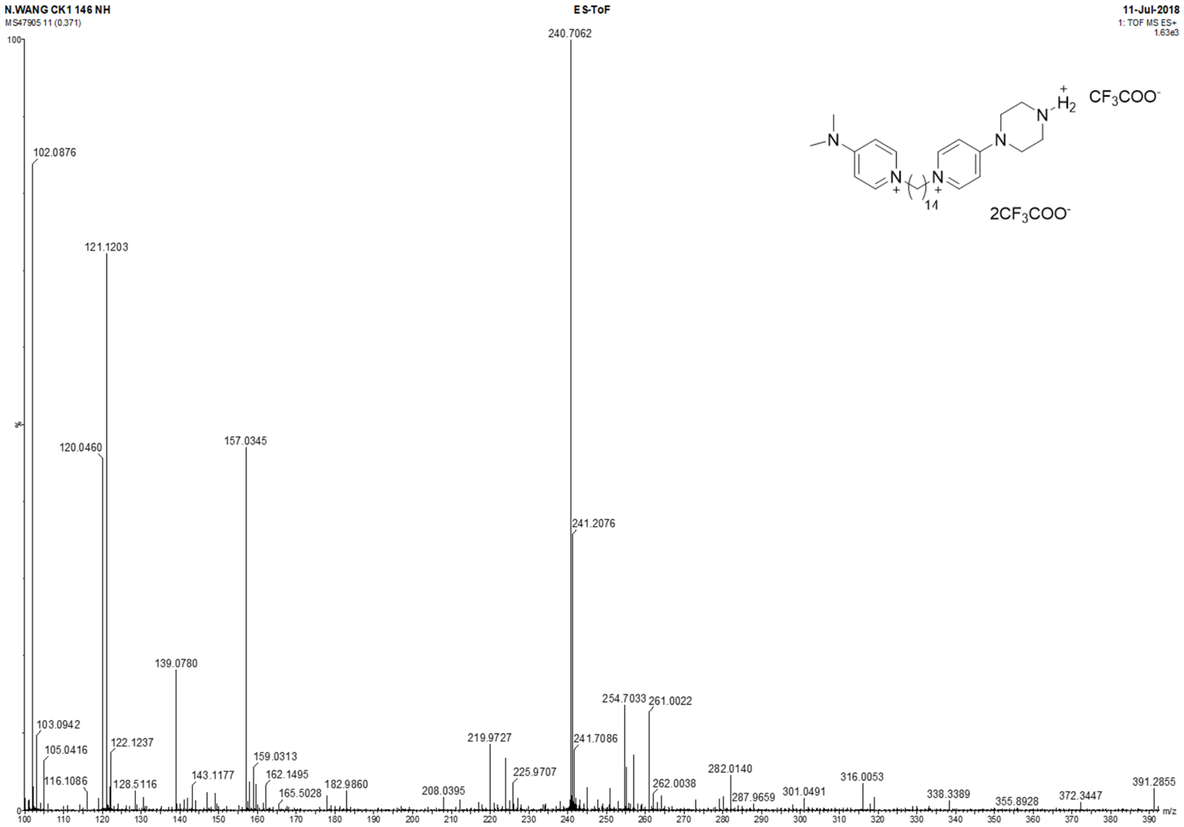

Figure S18. MS of **4**.

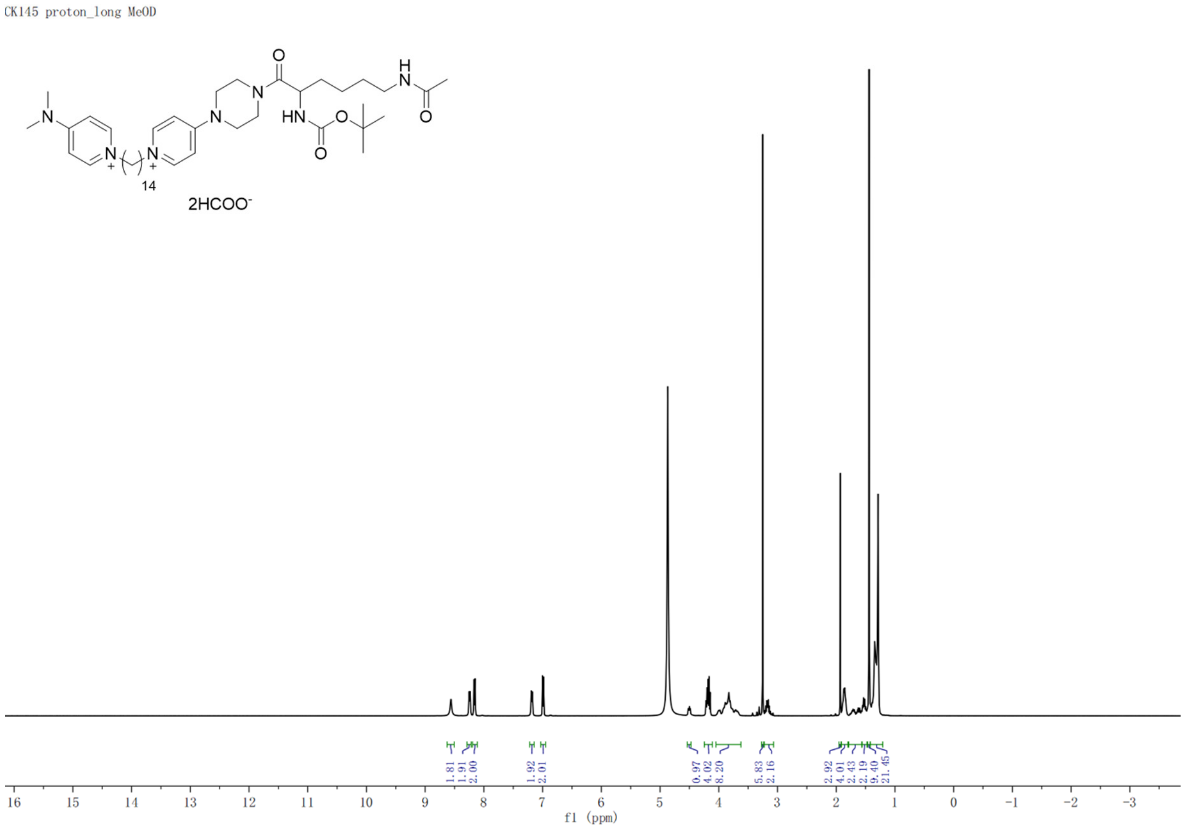

Figure S19. ^{1}H NMR of **5**.

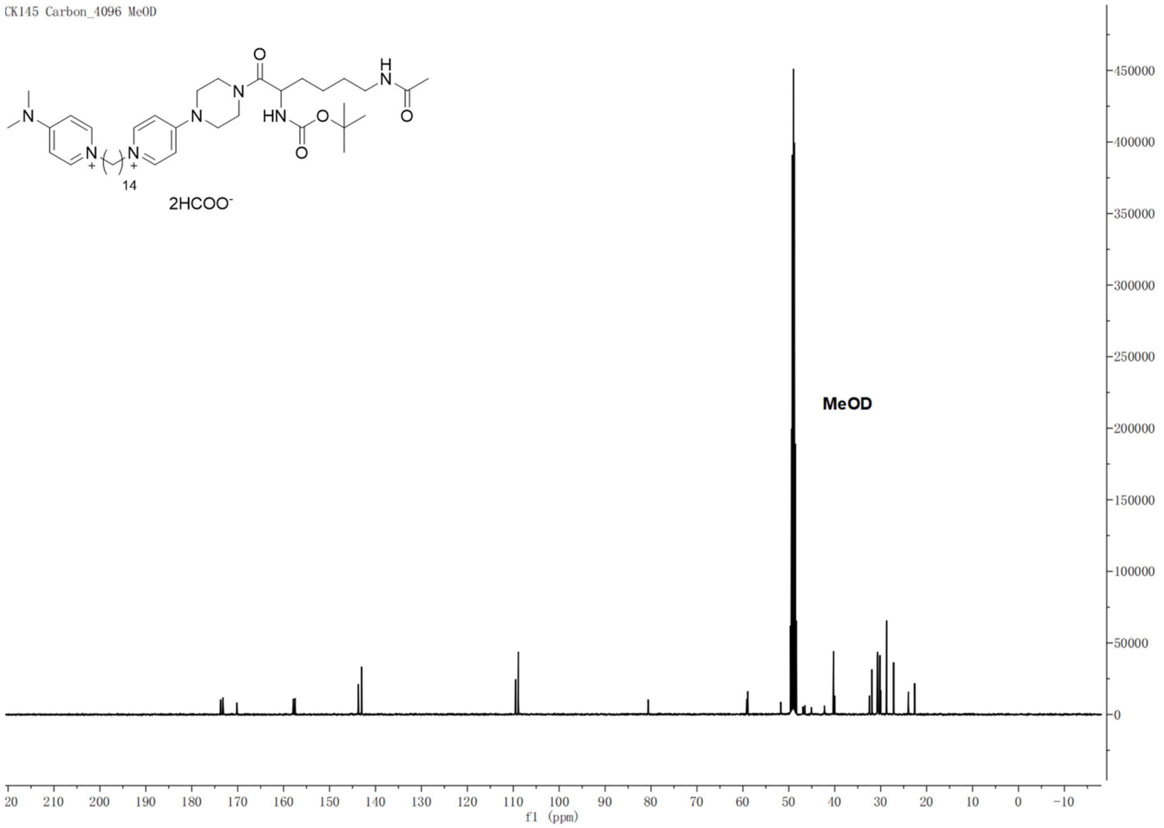

Figure S20. ^{13}C NMR of **5**.

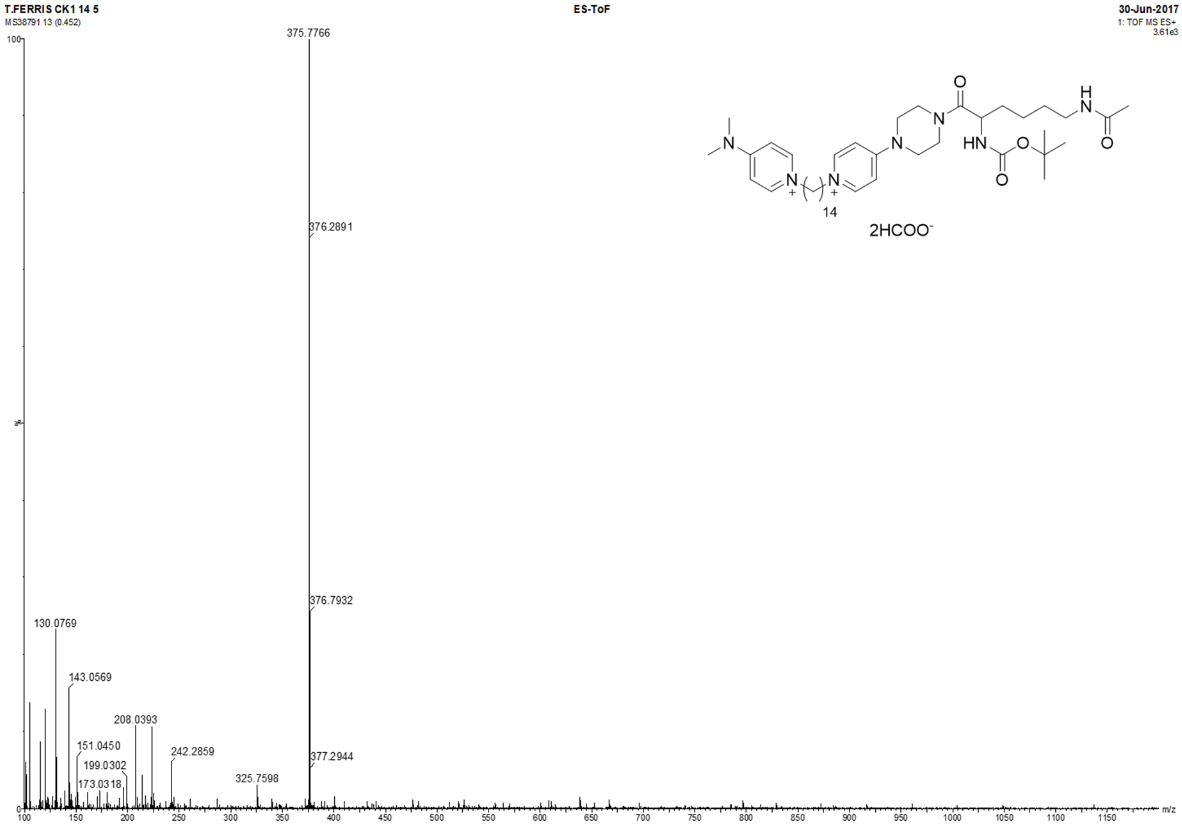

Figure S21. MS of **5**.

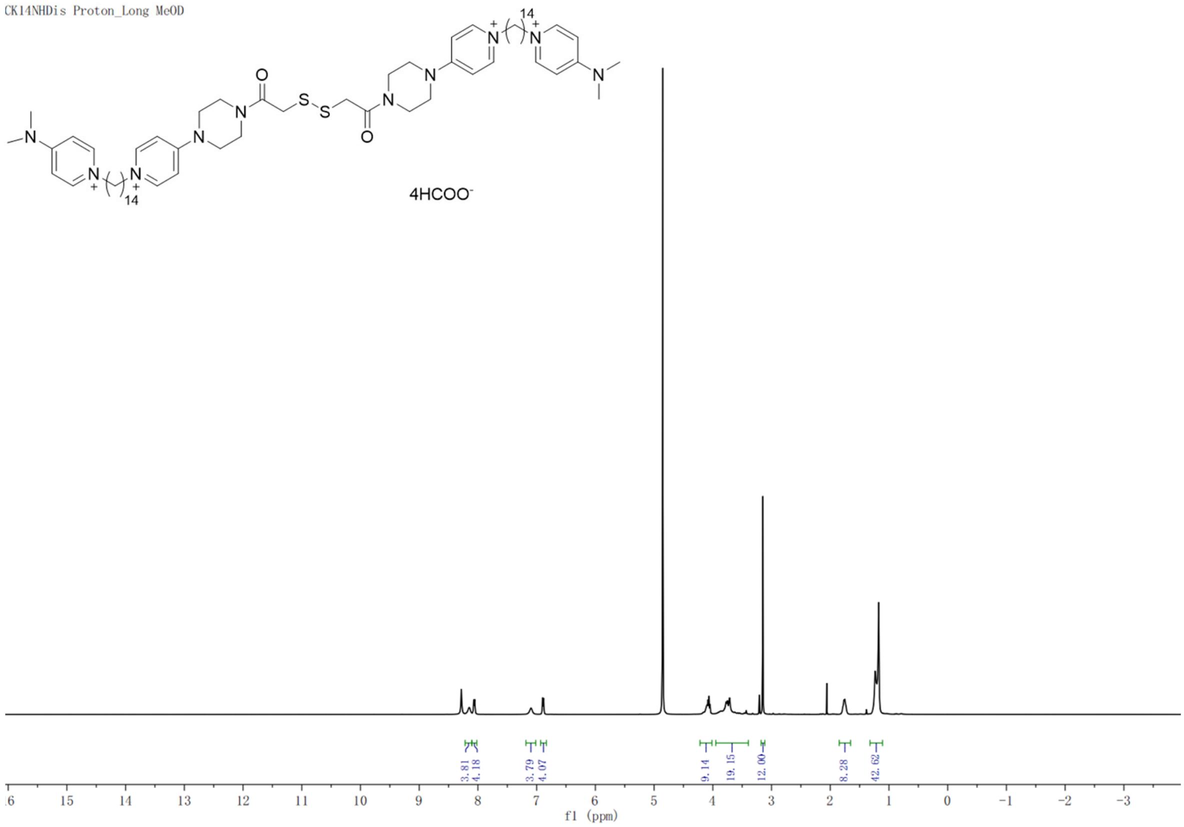

Figure S22. ^{1}H NMR of **6**.

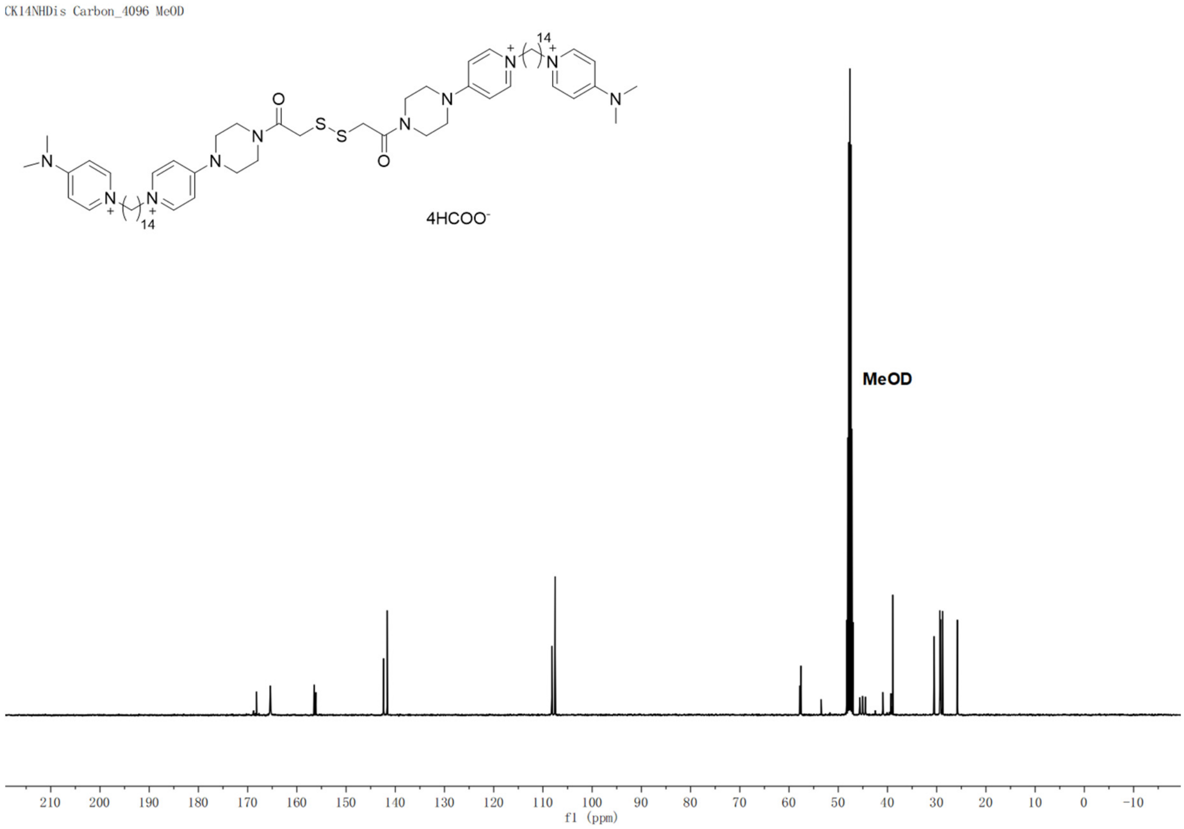

Figure S23. ^{13}C NMR of **6**.

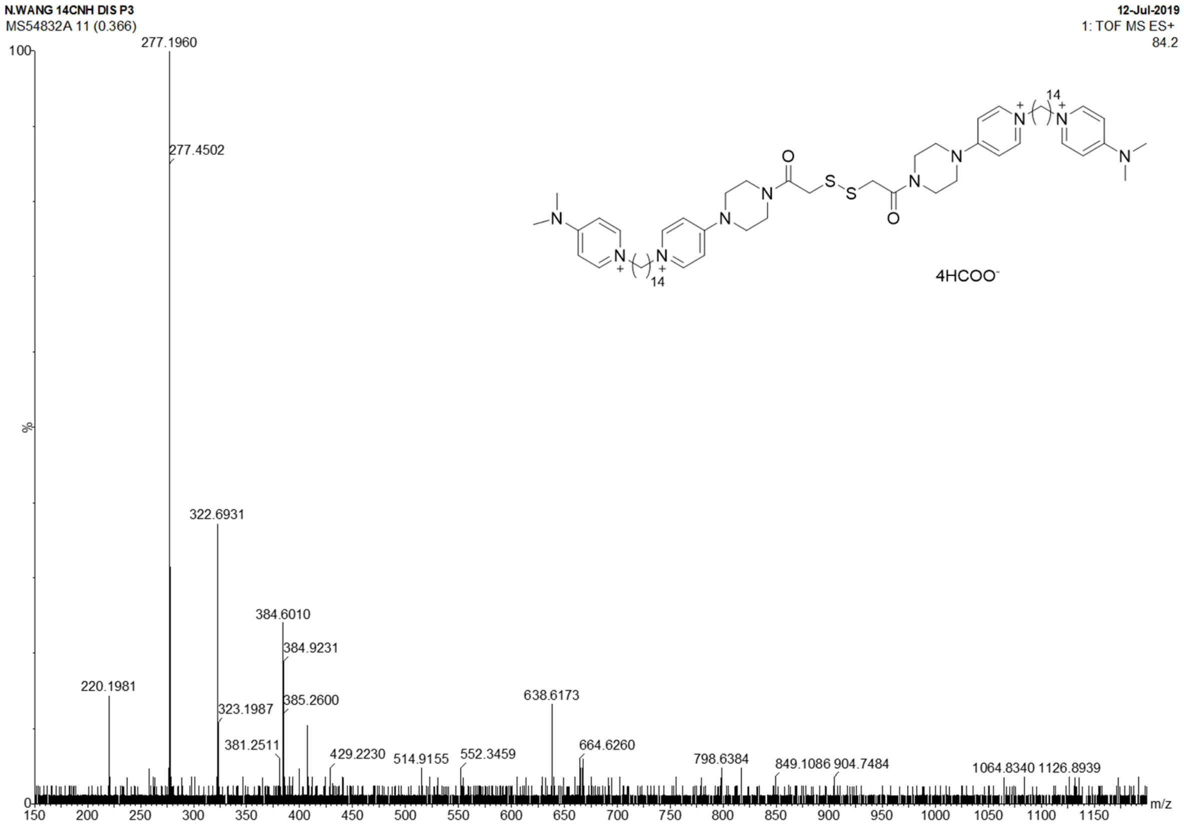

Figure S24. MS of **6**.

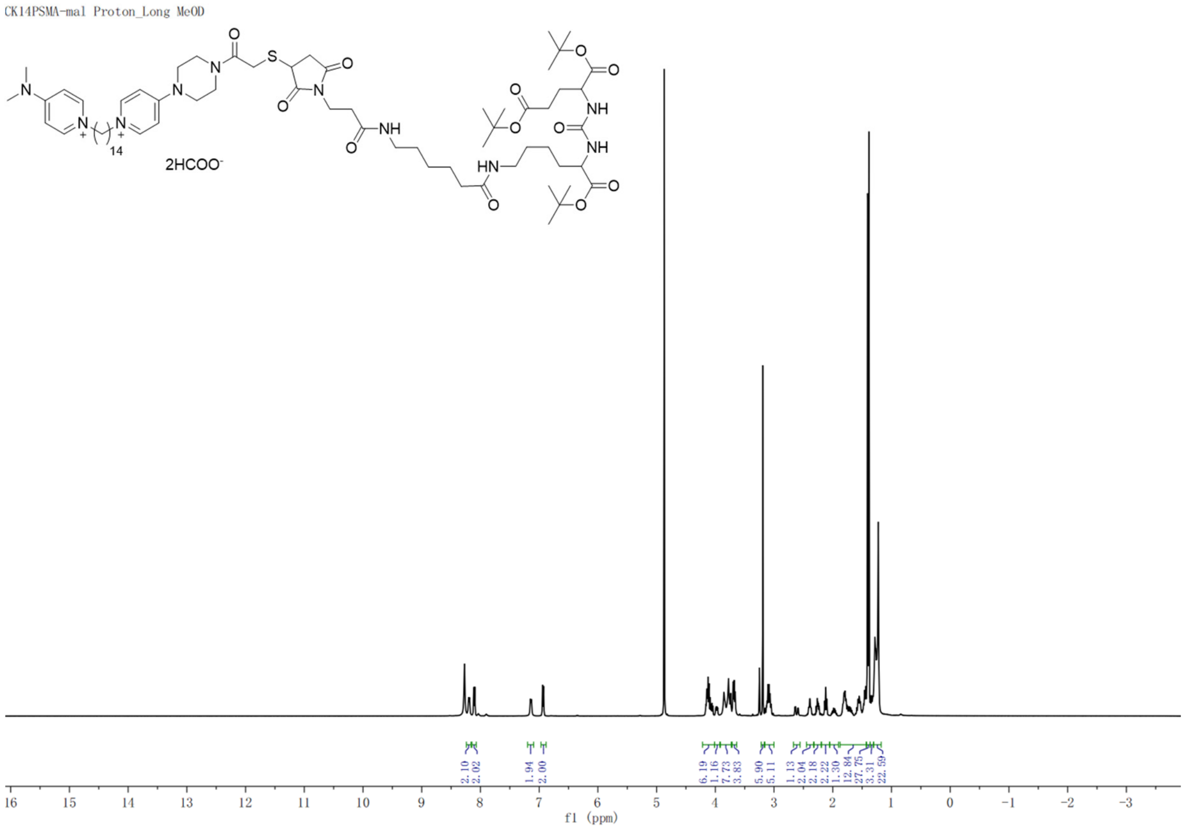

Figure S25. ^{1}H NMR of **7**.

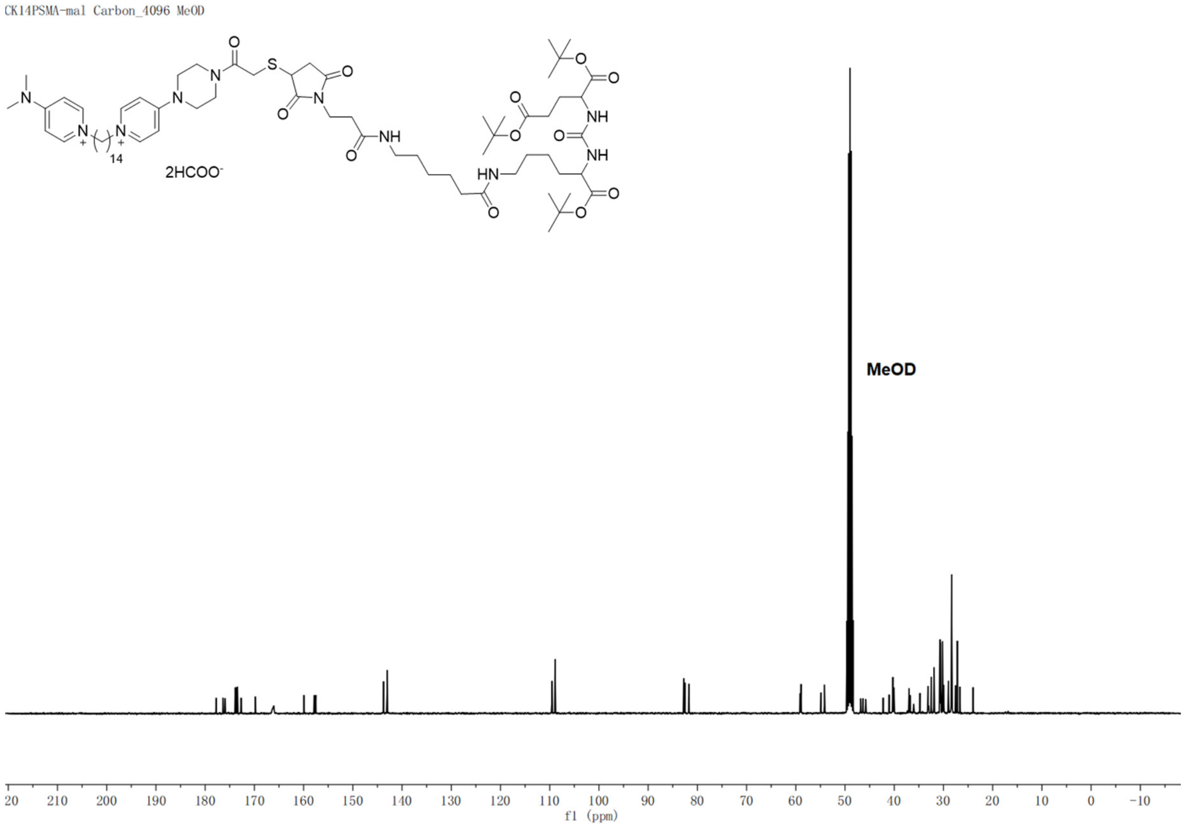

Figure S26. ^{13}C NMR of **7**.

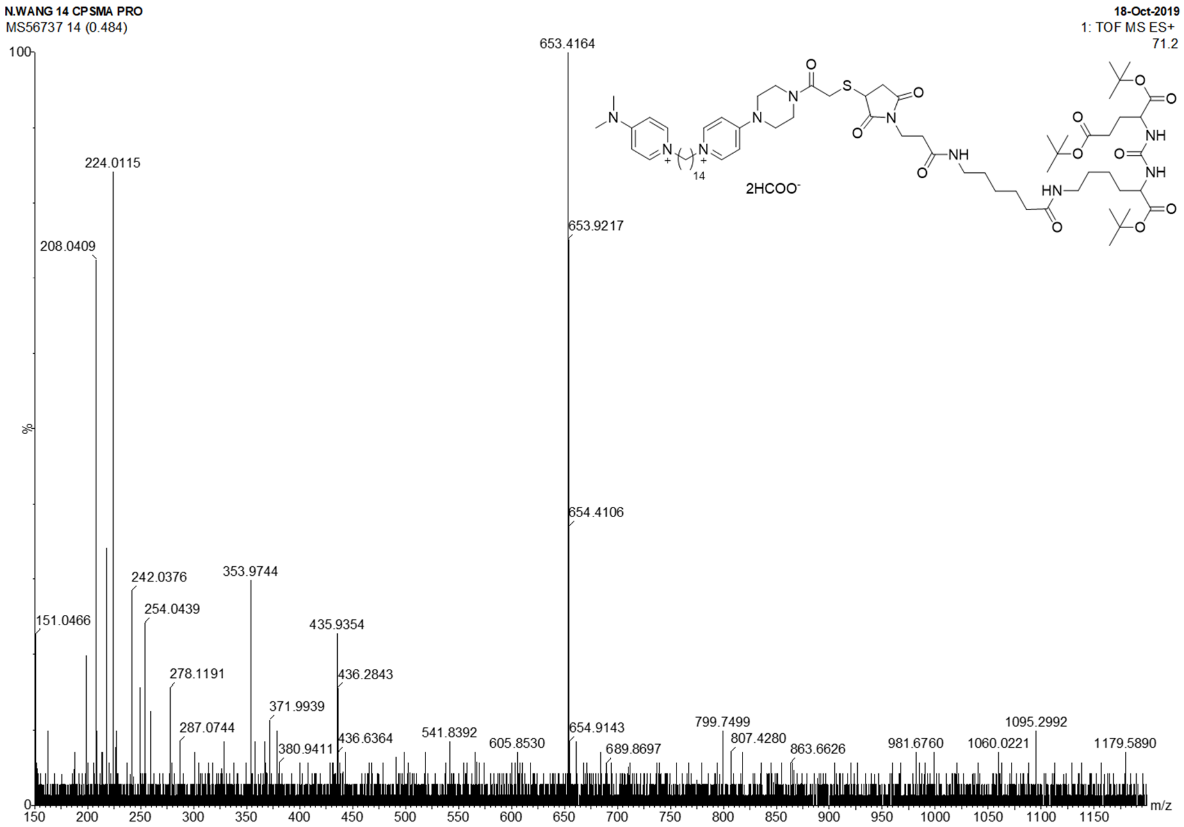

Figure S27. MS of **7**.

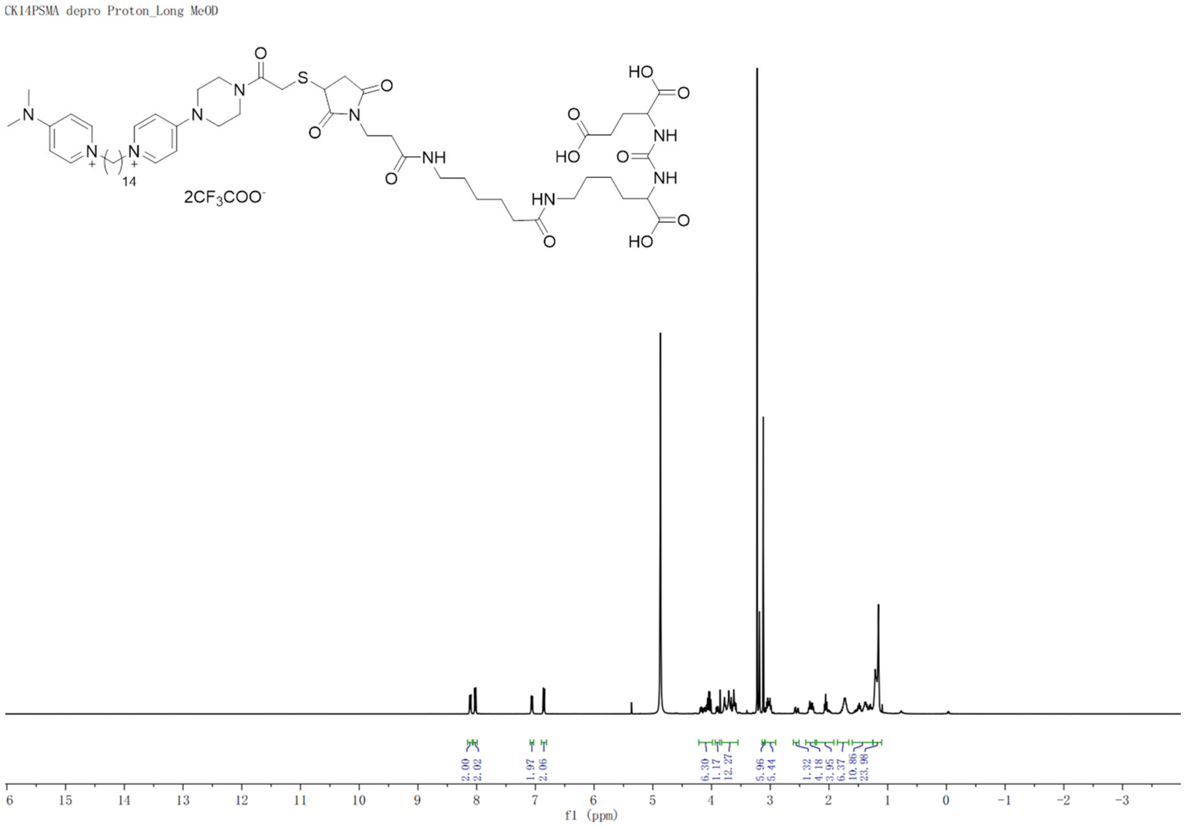

Figure S28. ^{1}H NMR of **8**.

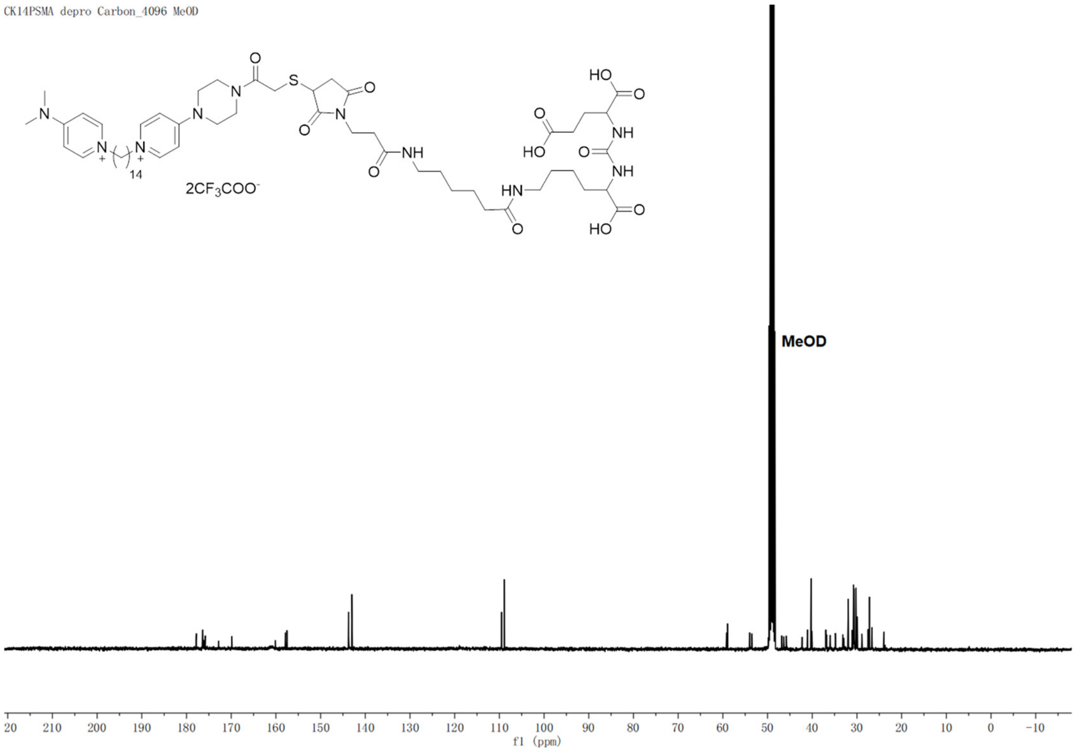

Figure S29. ^{13}C NMR of **8**.

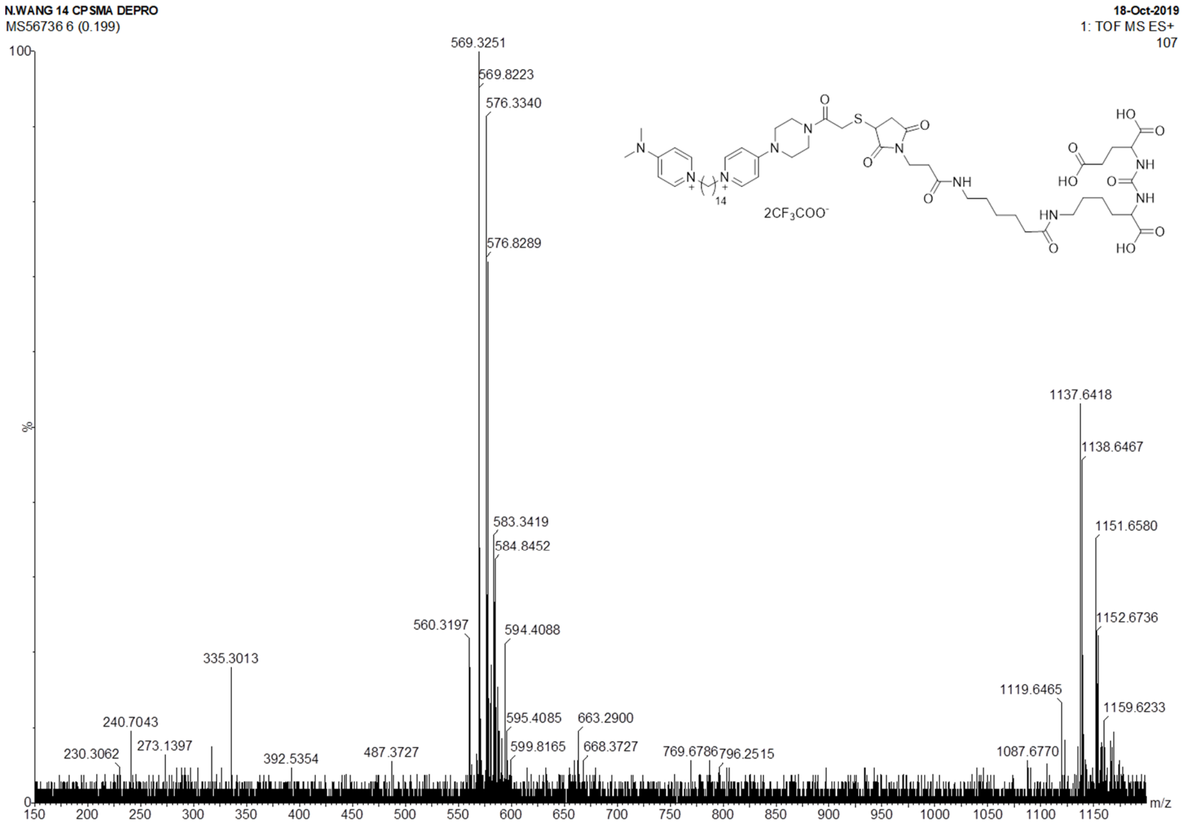

Figure S30. MS of **8**.

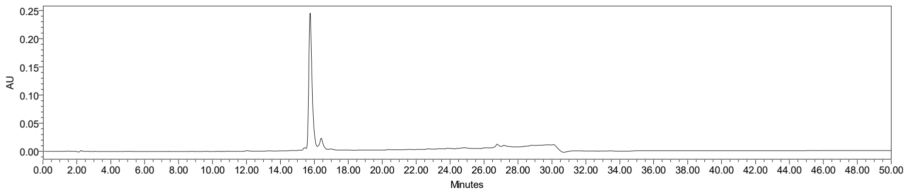

Figure S31. The purity check of **7** after complete deprotection by HPLC analysis (purity: 95%, HPLC method C).

5.0 Antiproliferative assay

Table S1. mRNA expression of four cell lines (HCT-116, HepG2, A549 and Caco-2) used in the CK145 studies, extracted from Cancer Cell Line Encyclopedia RNA sequencing database. Here HCT-116 cell line shows the highest mRNA expression level of HDAC I class (coloured in red).

Cell name / Type	HDAC1	HDAC2	HDAC3	HDAC8	CTSL
HCT-116_LARGE_INTESTINE	134.1379	23.4397	53.52749	3.12038	26.88584
HEPG2_LIVER	58.27163	12.09713	31.14372	2.59897	27.7715
A549_LUNG	49.61435	13.36945	27.13287	4.64499	167.203
CACO2_LARGE_INTESTINE	54.92482	21.73755	30.81502	2.93636	42.09802

Table S2. Inhibitory activity against HCT-116, A549, HepG2 and Caco-2 cancer cell lines (GI_{50}) with ICL-CCIC-0019, CK146 and CK145.

		GI_{50} (μM) ± SD	
**Cell line**	**ICL-CCIC-0019**	**CK146**	**CK145**
HCT-116	0.42 ± 0.03	4.76 ± 0.8	6.63 ± 0.2
A549	0.05 ± 0.005	2.07 ± 0.2	2.47 ± 0.06
HepG2	0.18 ± 0.01	0.21 ± 0.01	2.25 ± 0.06
Caco-2	1.19 ± 0.04	3.09 ± 0.05	10.8 ± 1

Table S3. Inhibitory activity against human 22Rv1, C4-2B, LNCap, R1AD1, R1-D567, PC3, PNT1A and HCT-116 cell lines (GI_{50}) with ICL-CCIC-0019, CK146 and CK147.

			GI_{50} (μM) ± SD	
**PSMA**	**Cell line**	**ICL-CCIC-0019**	**CK146**	**CK145**
+	22Rv1	0.91 ± 0.13	4.98 ± 1.50	45.64 ± 6.40
+	C4-2B	1.20 ± 0.31	4.22 ± 0.43	63.00 ± 12.12
+	LNCap	0.30 ± 0.06	1.92 ± 0.99	39.03 ± 8.56
–	R1AD1	0.40 ± 0.01	5.67± 2.33	53.40 ± 2.27
–	R1-D567	0.32 ± 0.00	0.87 ± 0.03	40.43 ± 4.56
–	PC3	0.79 ± 0.03	4.73 ± 0.81	84.92 ± 7.03
–	PNT1A	0.47 ± 0.08	3.00 ± 0.26	63.64 ±10.38
–	HCT-116	0.42 ± 0.03	4.76 ± 0.8	103.49 ± 6.27

6.0 Cellular uptake and *in vitro* metabolism study

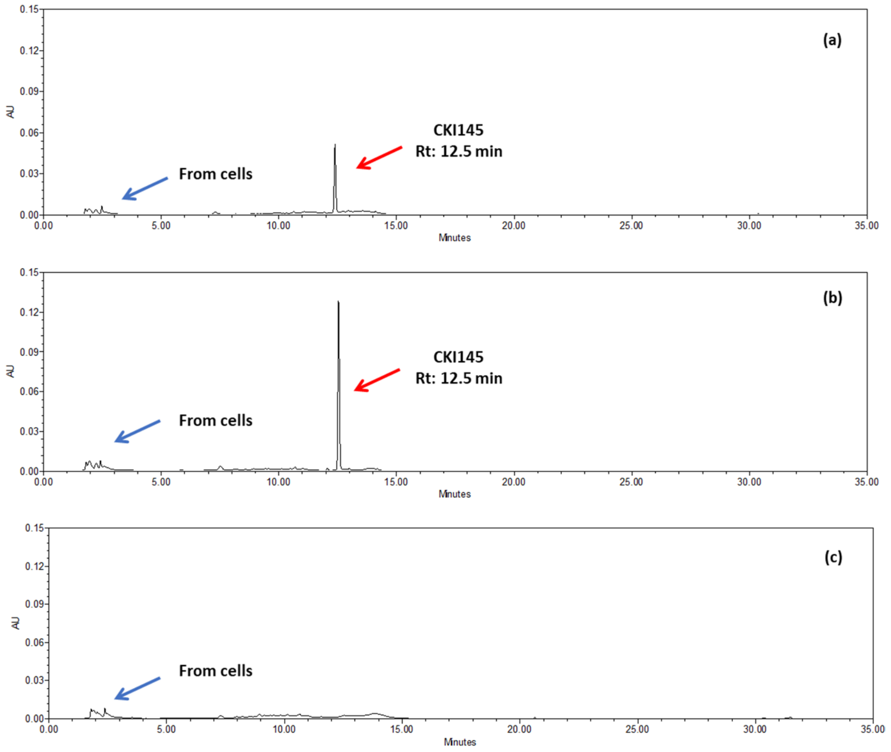

Figure S32. HPLC chromatograms of HCT-116 cell extracts after 1 h and 2 h incubation with/without CK145 (50 µM of 5 mL RPMI medium). The peaks from cells and the peak of CK145 were labelled with blue and red arrows respectively. (a) The HPLC chromatogram of HCT-116 extracts after 1h incubation with CK145; (b) The HPLC chromatogram of HCT-116 extracts after 2h incubation with CK145; (c) The HPLC chromatogram of HCT-116 extracts after 2h incubation without CK145 (control group). Similar results were observed in three independent experiments.

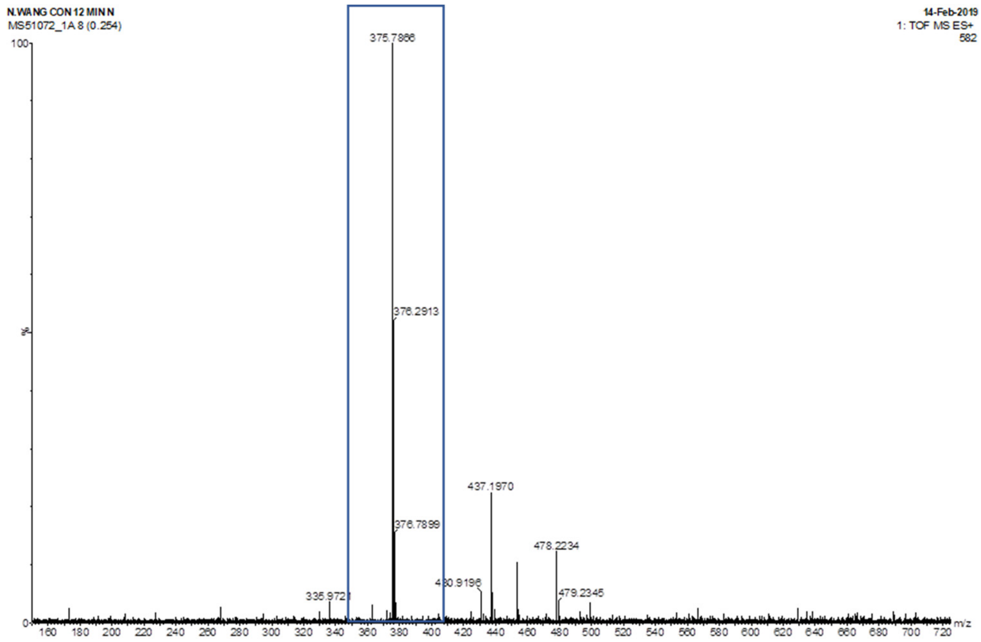

Figure S33. MS of the compound collected with a retention time of 12.5 min:sec on HPLC (HPLC method C). The compound detected by HPLC was confirmed to be CK145 (highlighted).

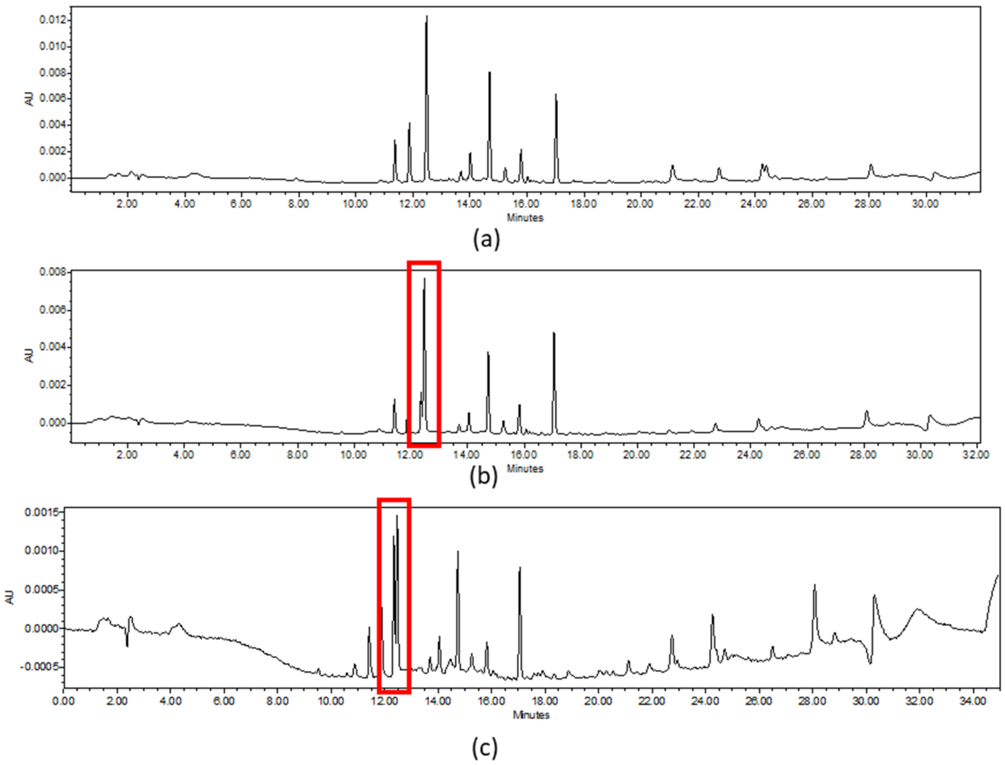

Figure S34. HPLC chromatograms of CK146 detection in medium after treatment. HPLC method C in Supplementary; wavelength: 294 nm. (a) control group; (b) 1 h incubation; (c) 2 h incubation. The peaks of CK145 highlighted overlapped with the peaks from medium. CK146 was supposed to have a retention time between 9 and 10 min, which was not detected in medium (LOD value: 1.382 μg/L).

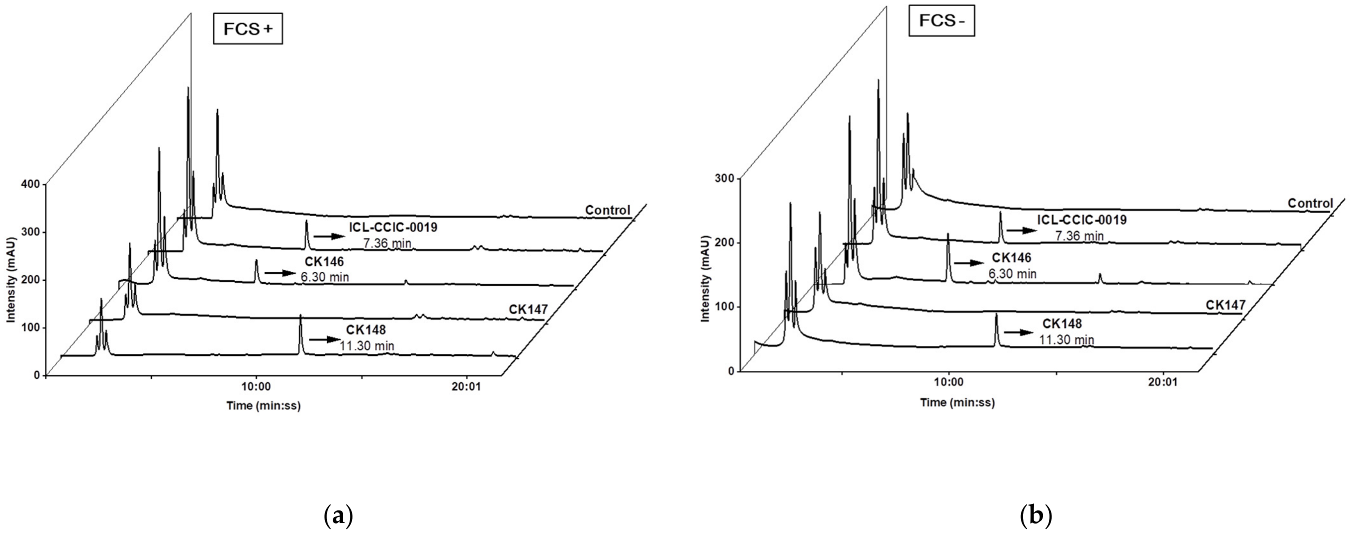

Figure S35. Cellular uptake study of ICL-CCIC-0019, CK146, CK147 and CK148 in C4-2B cells using medium with or without FCS. (a) HPLC chromatograms of compound uptake analysis using medium supplemented with 10% FCS; (b) HPLC chromatograms of compound uptake analysis using FCS-free medium. The detected compounds were arrowed with their retention times (min:sec).

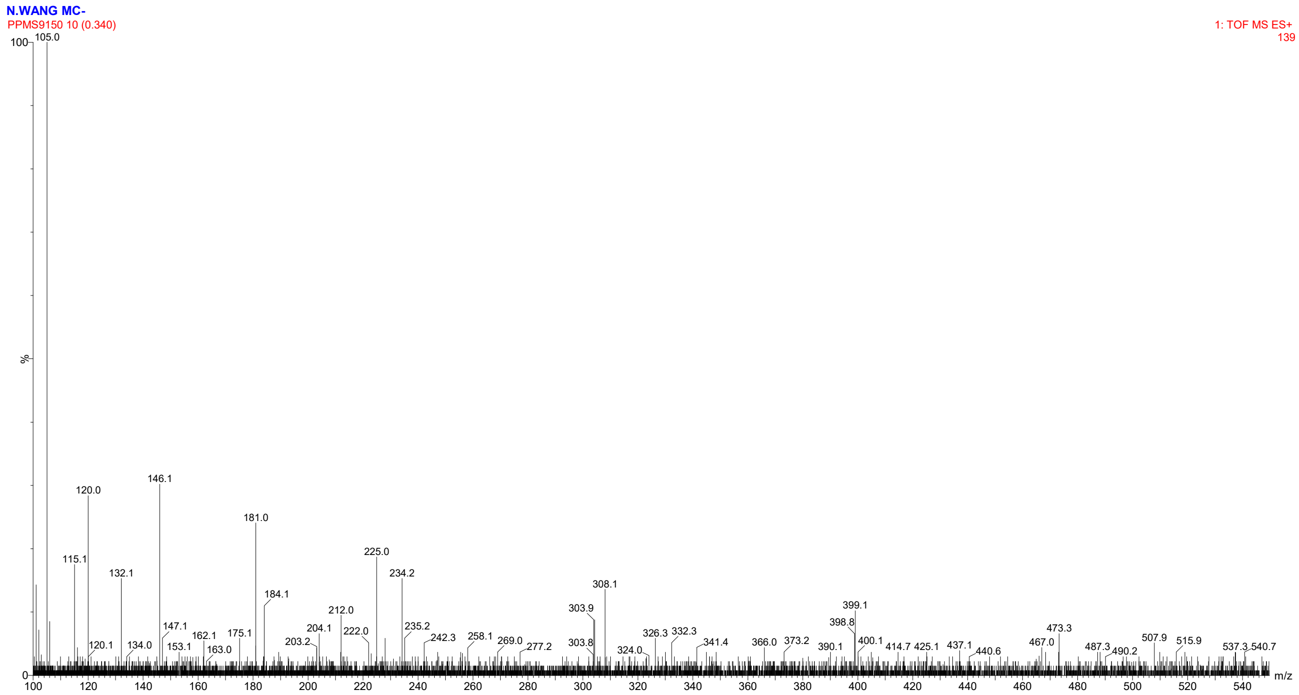

Figure S36. MS of the FCS-free medium without drug treatment.

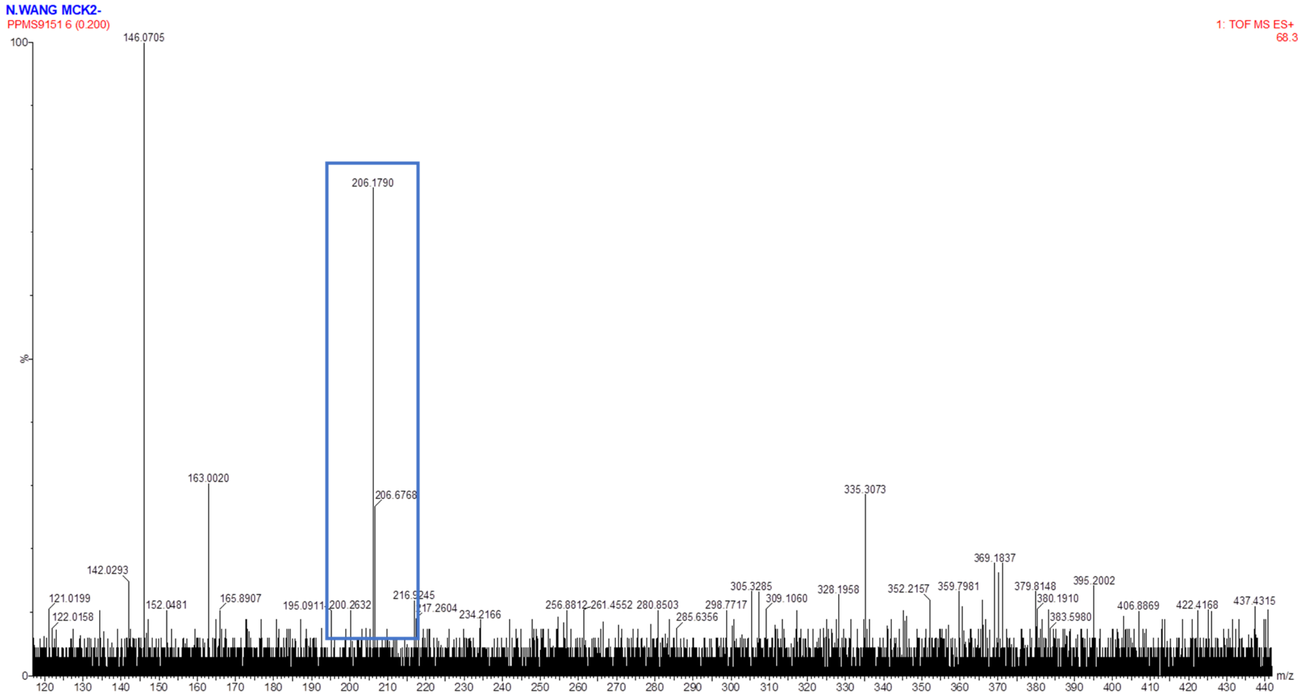

Figure S37. MS of the FCS-free medium incubated with ICL-CCIC-0019 for 4 h. The mass of ICL-CCIC-0019 was highlighted. No metabolites from ICL-CCIC-0019 were detected.

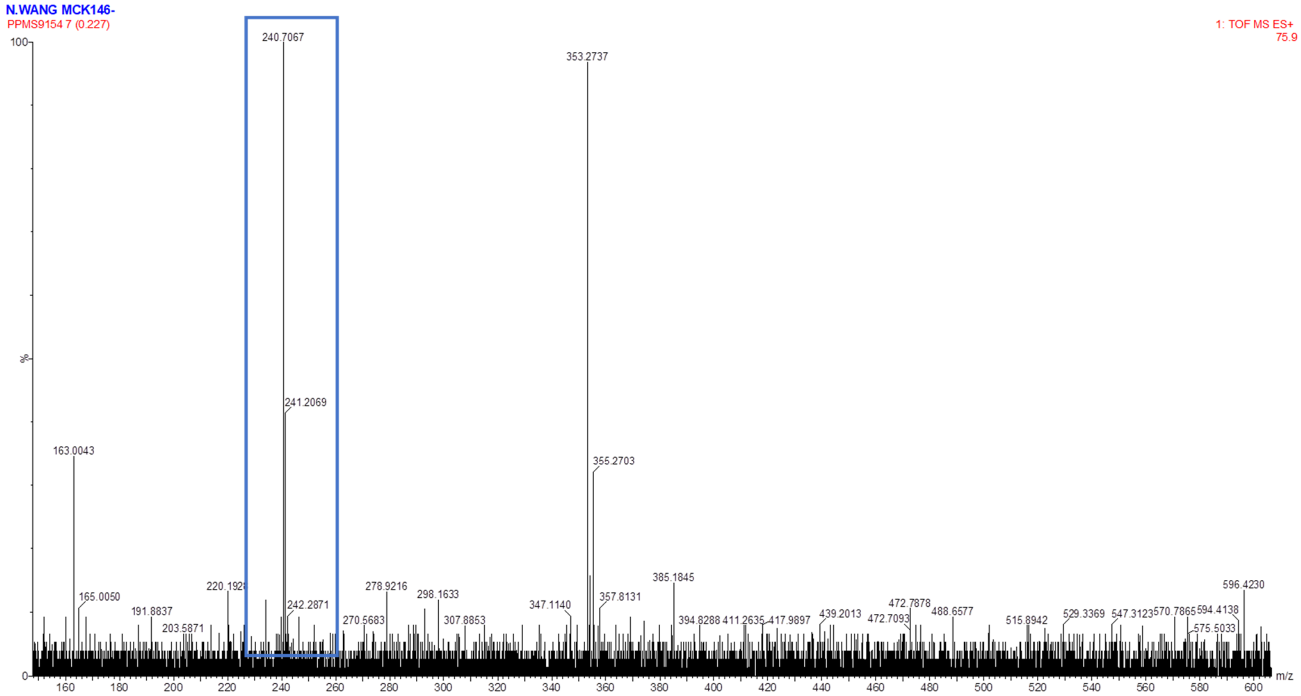

Figure S38. MS of the FCS-free medium incubated with CK146 for 4 h. The mass of CK146 was highlighted. No metabolites from CK146 were detected.

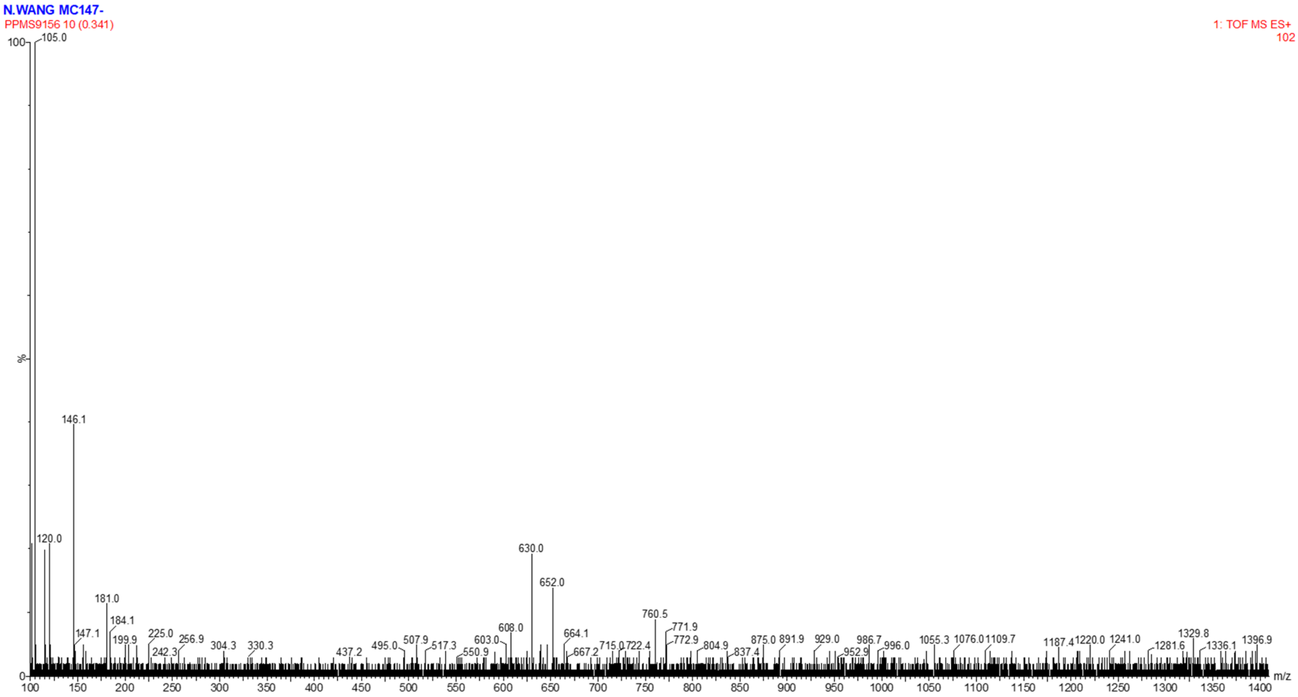

Figure S39. MS of the FCS-free medium incubated with CK147 for 4 h. CK147 and any metabolites from CK147 were not detected.

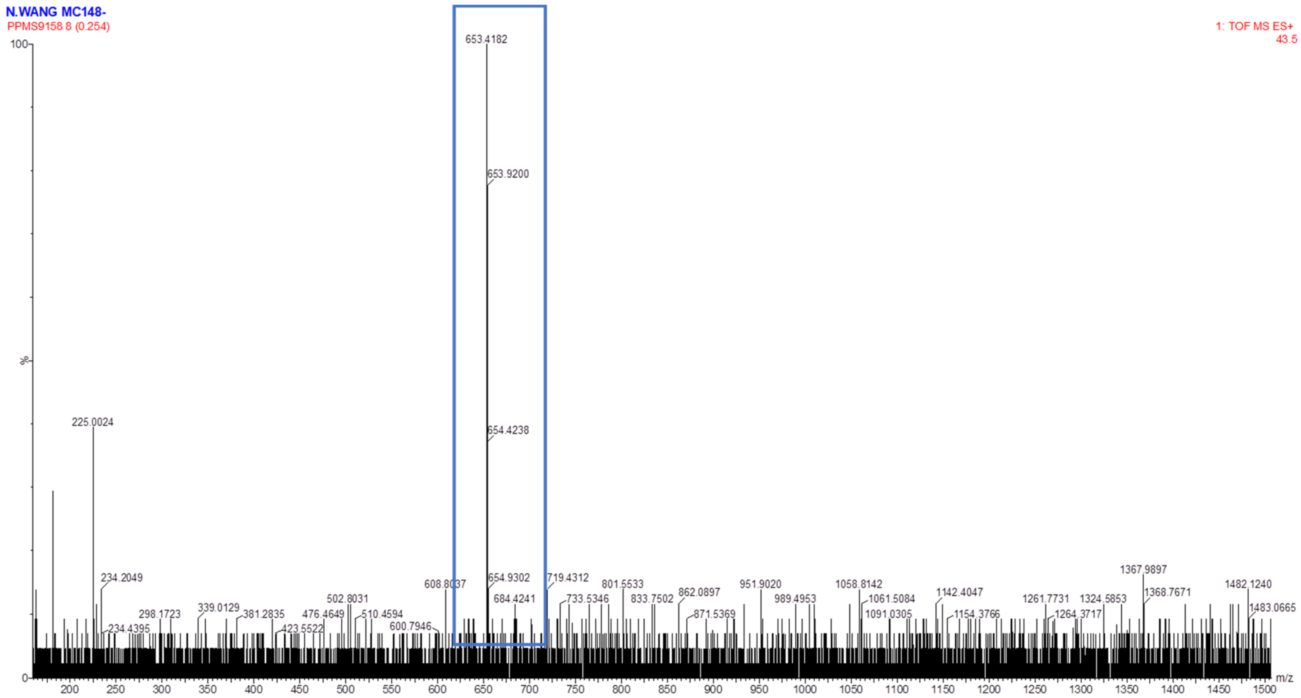

Figure S40. MS of the FCS-free medium incubated with CK148 for 4 h. The mass of CK148 was highlighted. No metabolites from CK148 were detected.

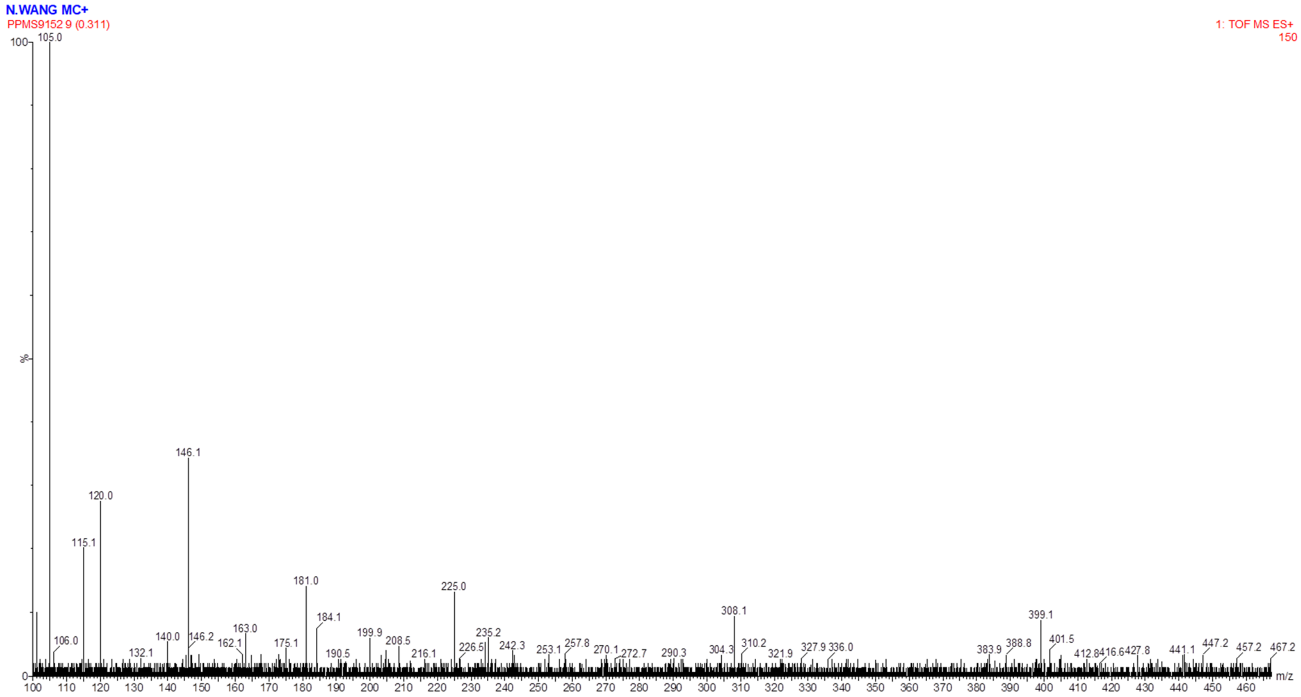

Figure S41. MS of the FCS supplemented medium without drug treatment.

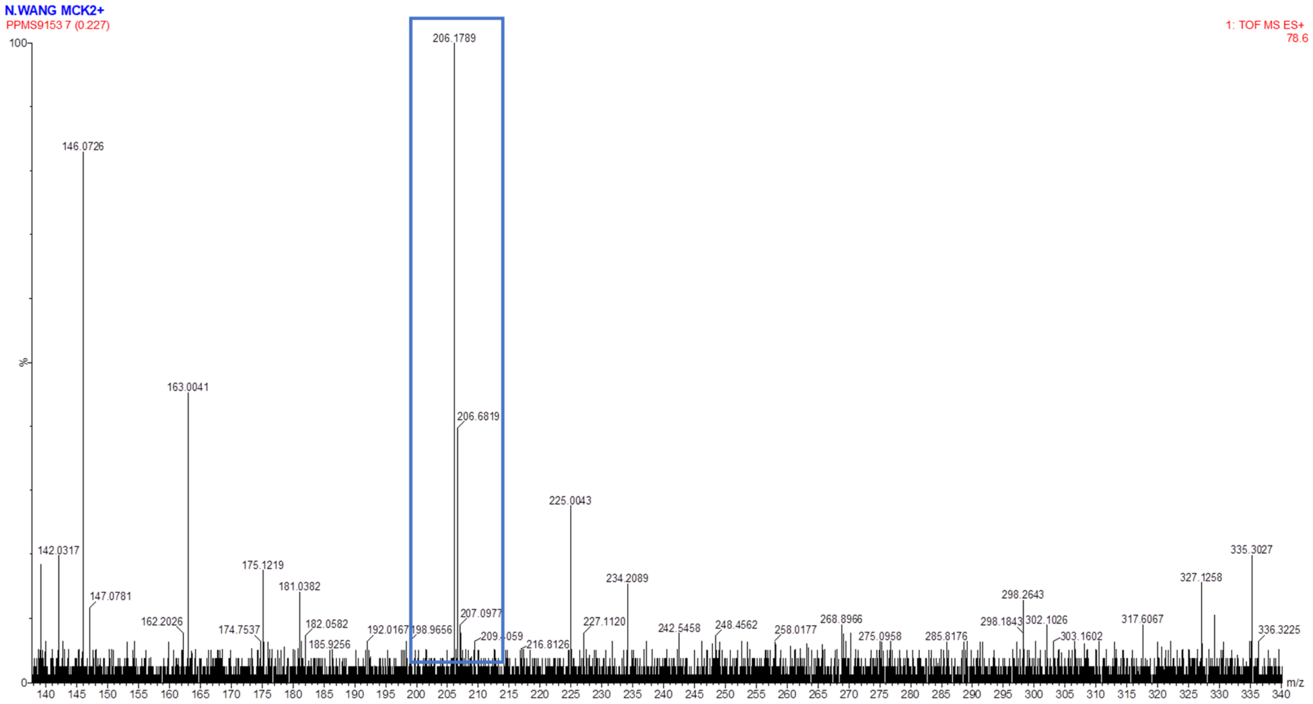

Figure S42. MS of the FCS supplemented medium incubated with ICL-CCIC-0019 for 4 h. The mass of ICL-CCIC-0019 was highlighted. No metabolites from ICL-CCIC-0019 were detected.

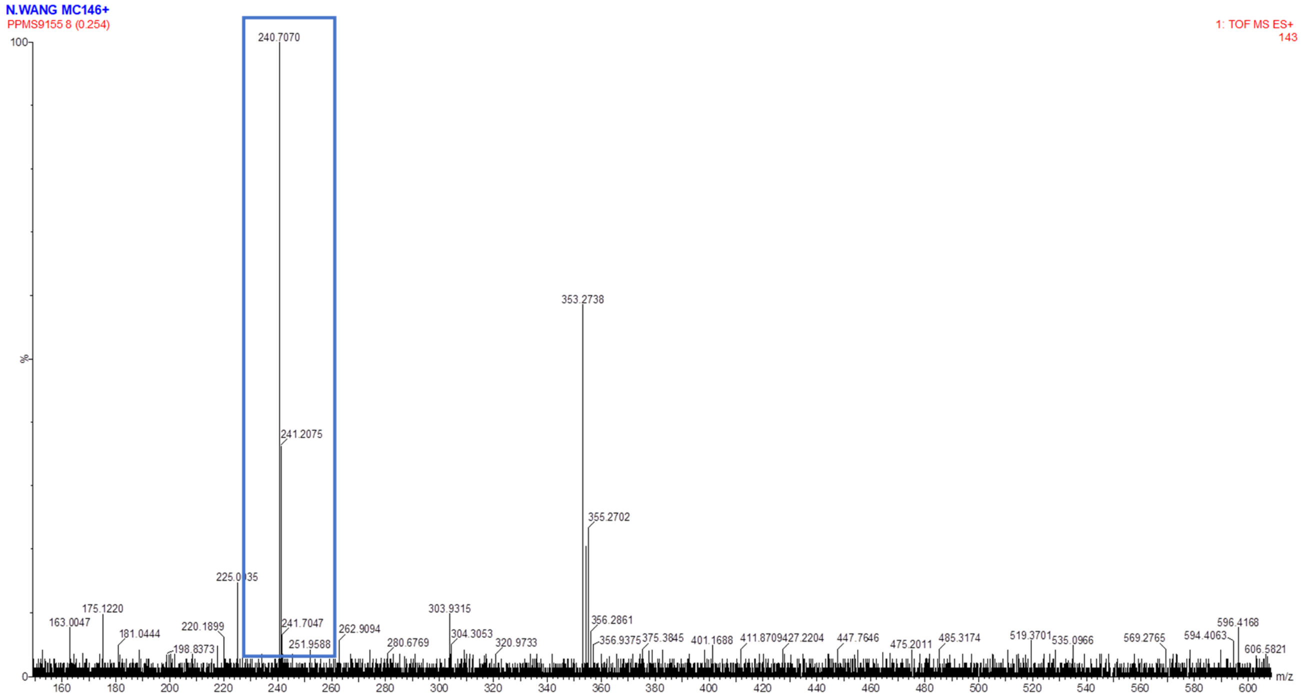

Figure S43. MS of the FCS supplemented medium incubated with CK146 for 4 h. The mass of CK146 was highlighted. No metabolites from CK146 were detected.

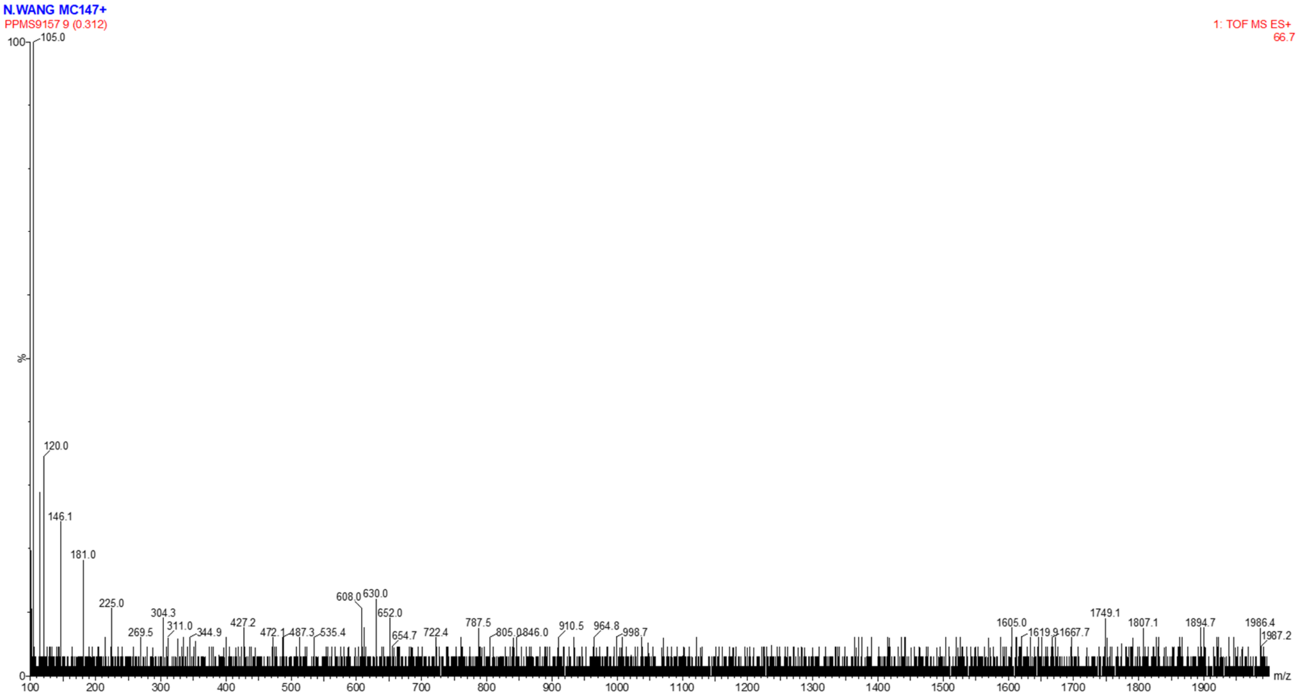

Figure S44. MS of the FCS supplemented medium incubated with CK147 for 4 h. CK147 and any metabolites from CK147 were not detected.

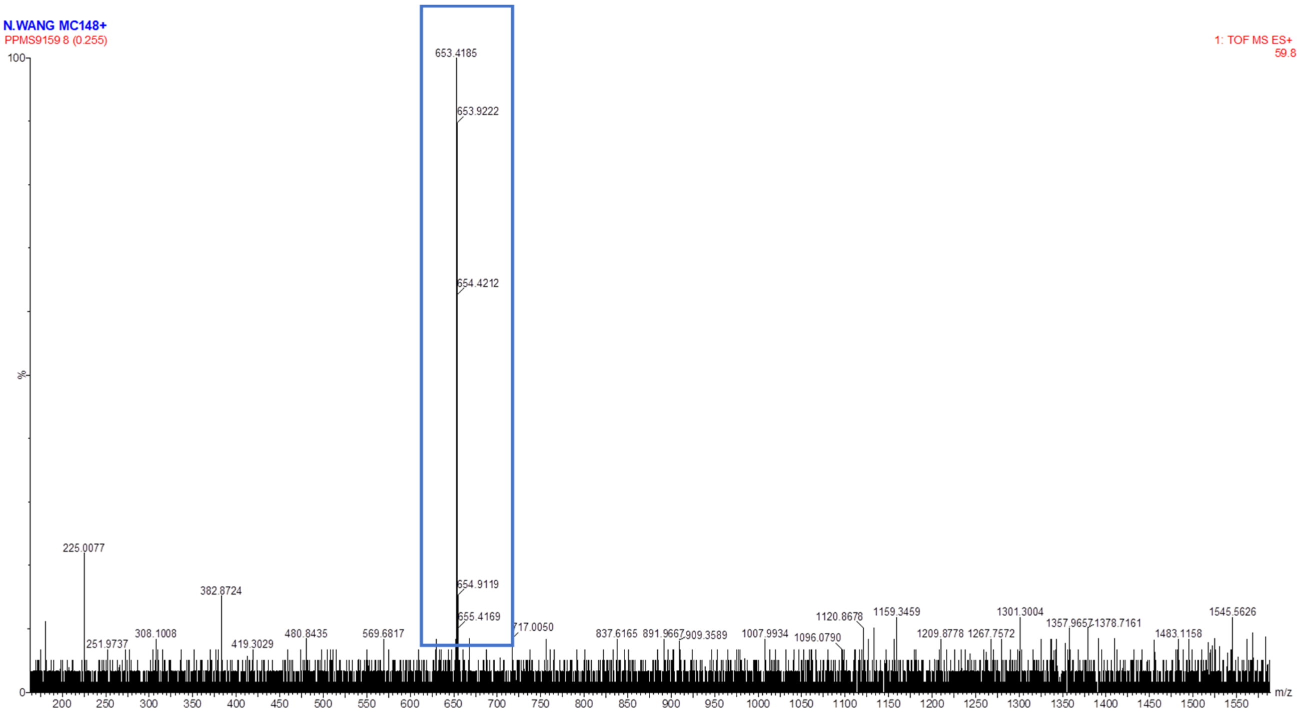

Figure S45. MS of the FCS supplemented medium incubated with CK148 for 4 h. The mass of CK148 was highlighted. No metabolites from CK148 were detected.
